# Accurate prediction of the kinetic sequence of physicochemical states using generative artificial intelligence†

**DOI:** 10.1039/d5sc00108k

**Published:** 2025-04-10

**Authors:** Palash Bera, Jagannath Mondal

**Affiliations:** a Tata Institute of Fundamental Research Hyderabad Telangana 500046 India palashb@tifrh.res.in jmondal@tifrh.res.in

## Abstract

Capturing the time evolution and predicting kinetic sequences of states of physicochemical systems present significant challenges due to the precision and computational effort required. In this study, we demonstrate that ‘Generative Pre-trained Transformer (GPT)’, an artificial intelligence model renowned for machine translation and natural language processing, can be effectively adapted to predict the dynamical state-to-state transition kinetics of biologically relevant physicochemical systems. Specifically, by using sequences of time-discretized states from Molecular Dynamics (MD) simulation trajectories akin to the vocabulary corpus of a language, we show that a GPT-based model can learn the complex syntactic and semantic relationships within the trajectory. This enables GPT to predict kinetically accurate sequences of states for a diverse set of biomolecules of varying complexity, at a much quicker pace than traditional MD simulations and with a better efficiency than other baseline time-series prediction approaches. More significantly, the approach is found to be equally adept at forecasting the time evolution of out-of-equilibrium active systems that do not maintain detailed balance. An analysis of the mechanism inherent in GPT reveals the crucial role of the ‘self-attention mechanism’ in capturing the long-range correlations necessary for accurate state-to-state transition predictions. Together, our results highlight generative artificial intelligence's ability to generate kinetic sequences of states of physicochemical systems with statistical precision.

## Abstract 2

GPT-based generative modeling of MD trajectories enables efficient prediction of state transitions by capturing long-range correlations, offering accurate kinetic and thermodynamic forecasts for diverse physicochemical systems.

## Graphical abstract

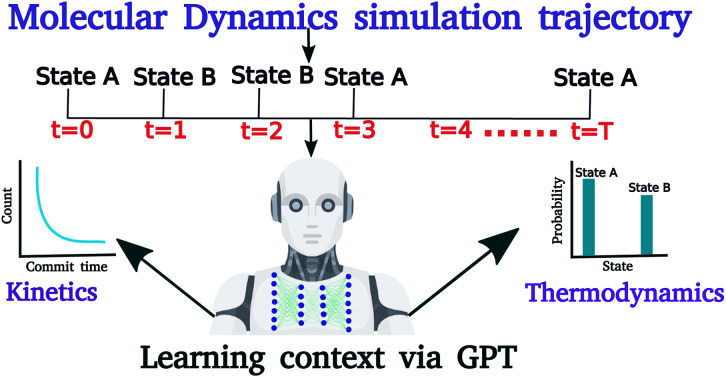

## Introduction

The time evolution of any physical system undergoes various state-to-state transitions. Understanding the dynamics of these systems particularly at the molecular level poses a significant challenge due to the complexity of their transitions between various states. Traditional approaches, such as molecular dynamics simulations (MDs), offer valuable insights into these transitions. However, MDs are computationally very expensive, limiting their applicability to large-scale systems or long-term predictions. Moreover, describing the actual phase space of a physical system typically involves handling data of very high dimensions. The lower dimensional representation of this data along some order parameters can provide information about various states and transitions between them. Nonetheless, achieving a comprehensive understanding of these transitions over a very long time requires performing highly resource-intensive MD simulations.

To understand the long-timescale behavior from experimental and simulated trajectories, various kinetic models such as the Markov state model (MSM)^1–3^ and Hidden Markov model (HMM)^4–6^ were employed to predict state transitions and identify metastable states, thereby providing insights into the underlying mechanisms of molecular processes. The initial step in constructing these models involves discretizing trajectories into a specified number of states along some collective variables (CVs). To identify effective CVs for state decomposition, a range of techniques were employed, including linear methods like Principal Component Analysis (PCA)^7–9^ and time-lagged independent component analysis (tICA),^10–12^ as well as non-linear approaches, particularly machine learning (ML) techniques such as Autoencoders and time-lagged Autoencoders.^13–18^ Recently, an ML approach known as VAMPnets^19^ has been proposed, which combines the principles of Autoencoders and tICA to learn molecular kinetics. Notably, VAMPnets offer the potential to streamline the entire, lengthy process of constructing the MSM by substituting it with a single deep neural network. Another approach, known as dynamic graphical models (DGMs),^20^ offers an efficient alternative for predicting molecular kinetics and unobserved configurations, utilizing fewer parameters than the traditional MSM. As the field advances, various deep generative state-less molecular simulation surrogates, including Boltzmann Generators,^21^ Normalizing Flows,^22–24^ Implicit Transfer Operators (ITO),^25^ and Timewarp,^26^ have emerged as powerful tools for sampling and predicting molecular dynamics. These approaches aim to provide efficient and effective alternatives to conventional MD simulations by leveraging advanced computational techniques.

In recent years, state-of-the-art recurrent neural networks (RNNs) and large language models (LLMs) have become promising tools in addressing various challenges.^27–33^ However, recurrent neural networks (RNNs) and their advanced variant, long short-term memory (LSTM) networks,^34^ excel at capturing sequential patterns in time-series data and sequence-to-sequence tasks, overcoming the vanishing gradient problem. However, these precedent methods face limitations in modeling complex syntactic and semantic relationships in large language models (LLMs). To overcome these issues, the pioneering work by Vaswani *et al.*^33^ introduced the attention-based model known as Transformer. The concept of self-attention mechanisms can encode contextual information from the input text and generate coherent and contextually relevant responses. Although the original work of the Transformer was mainly designed for machine translation, the various parts of the Transformer can be used for different purposes. For instance, the encoder component can be applied to classification problems, while the decoder component can be used for sequence-to-sequence generation.

In our study, we have utilized the decoder component of the Transformer architecture to predict the kinetic sequence of states of diverse physicochemical as well as biologically relevant systems. Our investigations primarily focus on a set of systems of hierarchical complexity, ranging from hand-crafted three-state and four-state model potentials to a globular folded protein namely Trp-cage and an intrinsically disordered protein (IDP) α-synuclein. We demonstrate that our protocol can effectively learn the time evolution of different states in MD or other kinetic simulations along certain low-dimensional order parameters. As a result, the model can generate a sequence of states that are kinetically and thermodynamically accurate. Interestingly, the model is remarkably powerful, as it can accurately generate the sequence of states even for an active system that is out of equilibrium, as would be demonstrated for an active worm-like polymer chain and its passive counterpart. Moreover, for more complex systems, we have found that the attention mechanism plays a crucial role in maintaining the relationships between the states, enabling the model to generate the sequence of states correctly. Our results show that the GPT model outperforms traditional MSM and LSTM networks in predicting the kinetic sequence of states.

## Results

### Learning molecular dynamics trajectory using the transformer model

**Fig. 1:**
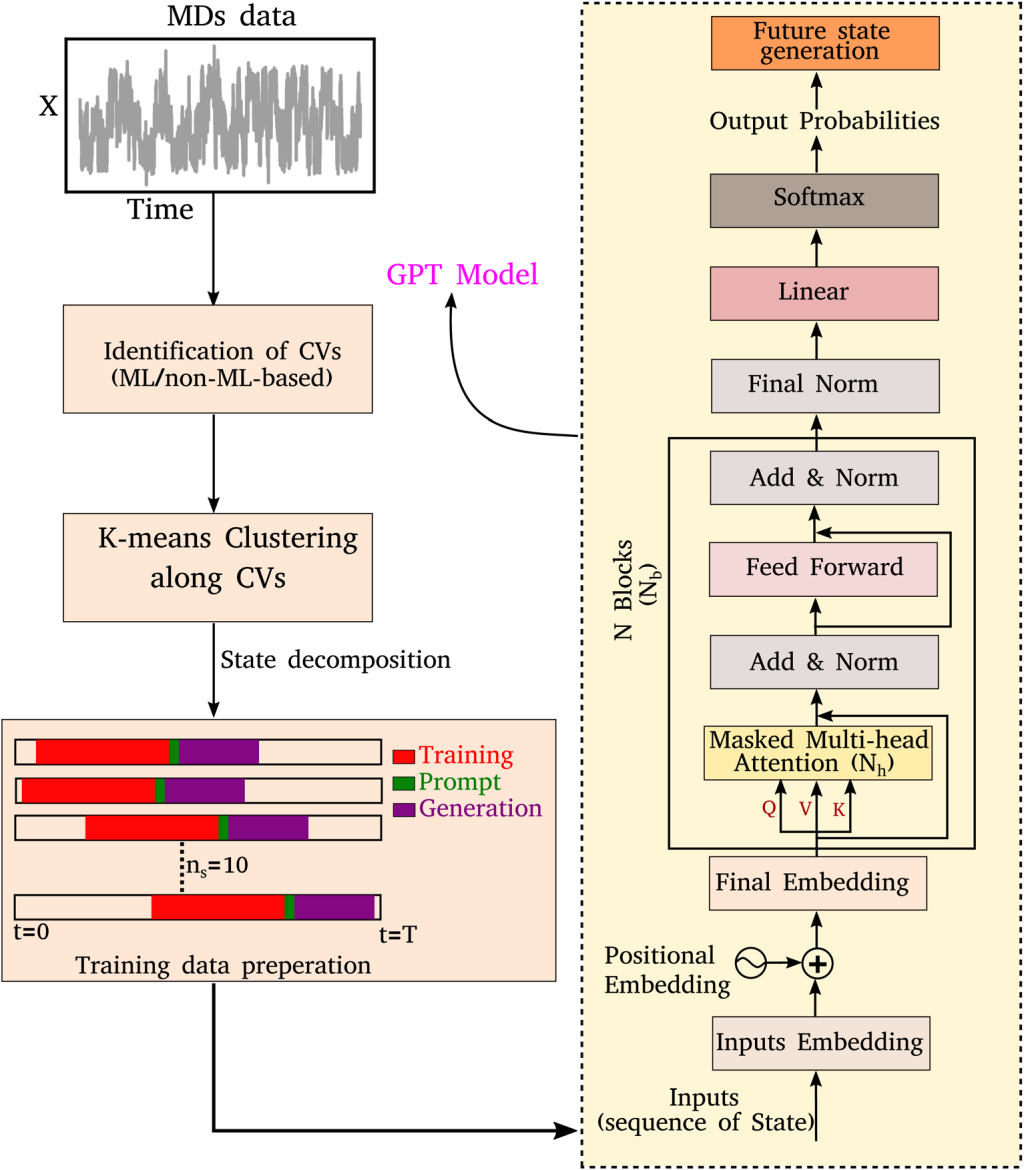
A schematic representation of training data preparation and the architecture of the decoder-only transformer. The left-hand side figure represents the schematic representation of the discretization of molecular dynamics simulation (MDs) trajectory achieved through the identification of collective variables (CVs) and K-means clustering. A total of *n*_s_ = 10 segments are randomly selected from the discretized trajectory to train an equal number of independent generative pre-trained transformer (GPT) models. Each trained model generates subsequent sequences starting from where the respective segment ended, using a few sequences as prompts. The right-hand side figure depicts the various layers of a decoder-only transformer. The model architecture consists of input embedding, positional embeddings, and multiple blocks of masked multi-head attention, normalization, and feed-forward layers. The model is optimized using cross-entropy as the loss function.

In any language model, the input is a series of characters or words. During the training process, the weights and biases of the various layers are optimized, enabling the model to understand the context and relationships between different parts of the input sequence. Once the model is trained, it can generate the subsequent sequence for a given input sequence in an auto-regressive manner. As illustrated in Fig. 1, here we implement a comprehensive scheme to learn the kinetic trajectory *via* the transformer and then to generate a sequence of states that could be segmented into three stages:

• (A) Segmentation of MD trajectories into discrete states.

• (B) Training a decoder-only transformer using MD-derived states as input.

• (C) Generating a kinetic sequence of states from the pre-trained transformer.

Below we describe the different stages of our scheme.

#### A. Discretization of molecular dynamics trajectory into meaningful state space

The time evolution of a physical system involves various conformational or state changes and transitions between them. Our study aims to predict the kinetic sequence of states of physicochemical systems using a large language model, specifically a decoder-only transformer. Interestingly, the state prediction problem can be mapped to the sequence generation of any language model where each state corresponds to a distinct vocabulary item within a corpus. For all of the systems, we have trained the model with molecular dynamics (MD) simulation trajectories to learn the occurrence of kinetic states. However, MD trajectories are continuous, requiring discretization of the trajectory into grid points or states before inputting it into a language model. For simple systems, the particle position can be used for discretization. However, complex systems with high dimensionality or degrees of freedom require defining some order parameters or collective variables (CVs) to discretize the trajectory into a certain number of states. To identify the various states of a physical system, we performed K-means clustering along CVs. Consequently, the trajectory is segmented into distinct sequences of states. These sequences can then be used as inputs for a decoder-only transformer model. Hereafter, these sequences of states will be referred to as sequences of tokens. For training the decoder-only transformer model, we randomly chose *n*_s_ segments from the discretized trajectory and trained *n*_s_ independent models with the same architecture. From each independently trained model, we generated the next sequences from where the corresponding segment ended by providing a few sequences as a prompt. All the results presented here are averaged over these independent runs. The left side of Fig. 1 illustrates a schematic overview of this process. The specific details regarding the amounts of data used for training, validation, and testing across the various systems are provided in Table S1.† Our approach and model are different from the methodology employed by Tsai *et al.*^35,36^ In their study, they utilized an LSTM-based architecture to learn the MD simulation trajectory, and the states were generated from the training data itself.

#### B. Training a decoder-only transformer with MD-derived states as input

Now we will delve into the architecture of the decoder-only transformer model. The neural network-based decoder-only transformer^33^ consists of several layers as depicted in the right side of Fig. 1. The first layer is an input embedding or token embedding layer that transforms each token of the input sequence into a dense vector. The dimension of the vector is a hyperparameter, which is called the embedding dimension. For a sequence of length *l* and embedding dimension *d*, this layer transforms each token into a d-dimensional vector. Consequently, the embedding layer generates a (*l* × *d*)-dimensional matrix, often referred to as the embedding or weight matrix. Throughout the training process, the elements of this weight matrix undergo optimization and the model will learn the semantic relationship between different tokens of the time sequence data.

To enable the GPT model to learn the sequence order of time series data, the positional information for each token is required, which can be achieved through positional embeddings. For an input sequence of length *l*, the position of the *k*^th^ token can be represented as follows:^33^

1 PE(*k*, 2*i*) = sin(*k*/10 000^2*i*/*d*^)

2 PE(*k*, 2*i* + 1) = cos(*k*/10 000^2*i*/*d*^)

where *d* is the dimension of the output embedding and, for each *k*(0 ≤ *k* < *l*), *i* can take a value from 0 to *d*/2. Hence, one can achieve the final embedding layer by adding these two embeddings.

The final embedding layer is followed by multiple blocks of layers (*N*_b_). Each block typically comprises various components, including masked multi-head attention with a specified number of heads, *N*_h_, normalization layers, and feed-forward layers. Among these, the masked multi-head attention layer is particularly significant, serving as a communication mechanism between tokens in the sequence. To calculate the key (*K*), query (*Q*), and value (*V*) tensors, the final embedded vector, denoted as *X*_f_, is multiplied with three trainable weight matrices (*W*_k_, *W*_q_, and *W*_v_) as *K* = *X*_f_·*W*_k_, *Q* = *X*_f_·*W*_q_, and *V* = *X*_f_·*W*_v_. The attention score *A*_s_ is then calculated as 
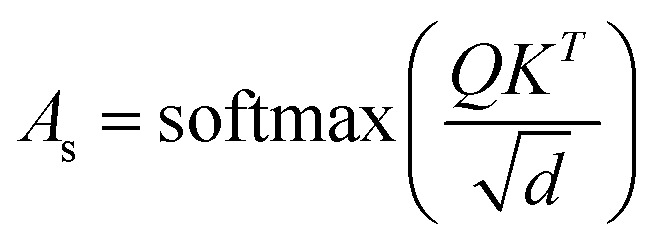
, and the output of the attention layer is given by^33^3
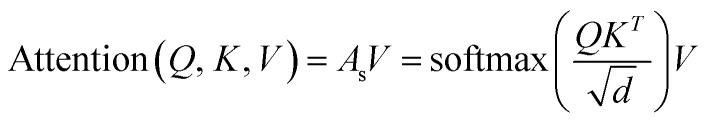
where 
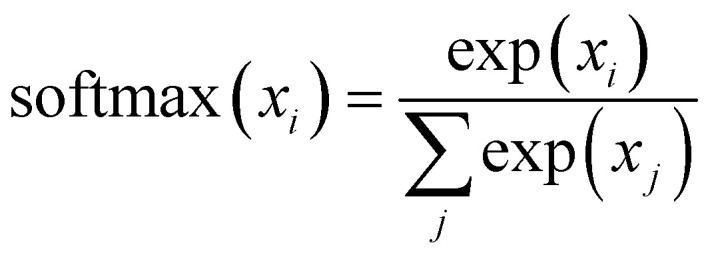
. This mechanism enables the model to discern the relative importance of tokens to each other, providing a clear sense of context and relationships between them.

Finally, the normalized outputs of the *N*_b_ layer are used as inputs of a fully connected dense linear layer. Given that the transformer model functions as a probabilistic model, the output of this dense layer is passed through a softmax function to yield categorical probabilities. We have used cross-entropy as our loss function to minimize the loss between the final output of the model *Ô*^(*t*)^ and the actual target output *O*^(*t*)^, which is defined as4
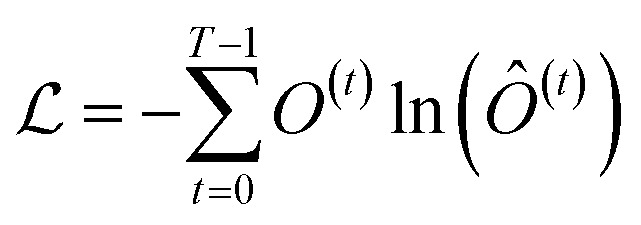
where *T* represents the total time of the trajectory, equivalent to the sequence length (*l*). The model has been trained over 10 000 epochs, with all hyperparameters across all of the systems provided in Table S2.† Fig. S1(a–f)† show the training and validation loss as a function of epochs for six distinct systems that would be elaborated in the upcoming sections of the present article. The plots indicate that the loss curves saturate or fluctuate slightly around the mean after a certain number of epochs, suggesting robust training of the transformer model without overfitting.

#### C. Generating kinetic sequence of states from the pre-trained transformer

After training the transformer model, it can generate any desired number of time series states by inputting an initial sequence of tokens as a prompt. For a given sequence, the model will generate a probability distribution over the entire vocabulary/states. From this probability distribution, the next element of the sequence can be sampled using a multinomial distribution (see supplemental results SR1 for details). As we have generated the kinetic sequence of states across all systems using our trained model, hereafter we will refer to it as the Generative Pre-Trained Transformer (GPT) model. The GPT model was built using PyTorch^37^ and our implementation is available on GitHub at the following URL: https://github.com/palash892/gpt_state_generation.

### GPT precisely captures inter-state transition kinetics in model multi-state systems

**Fig. 2:**
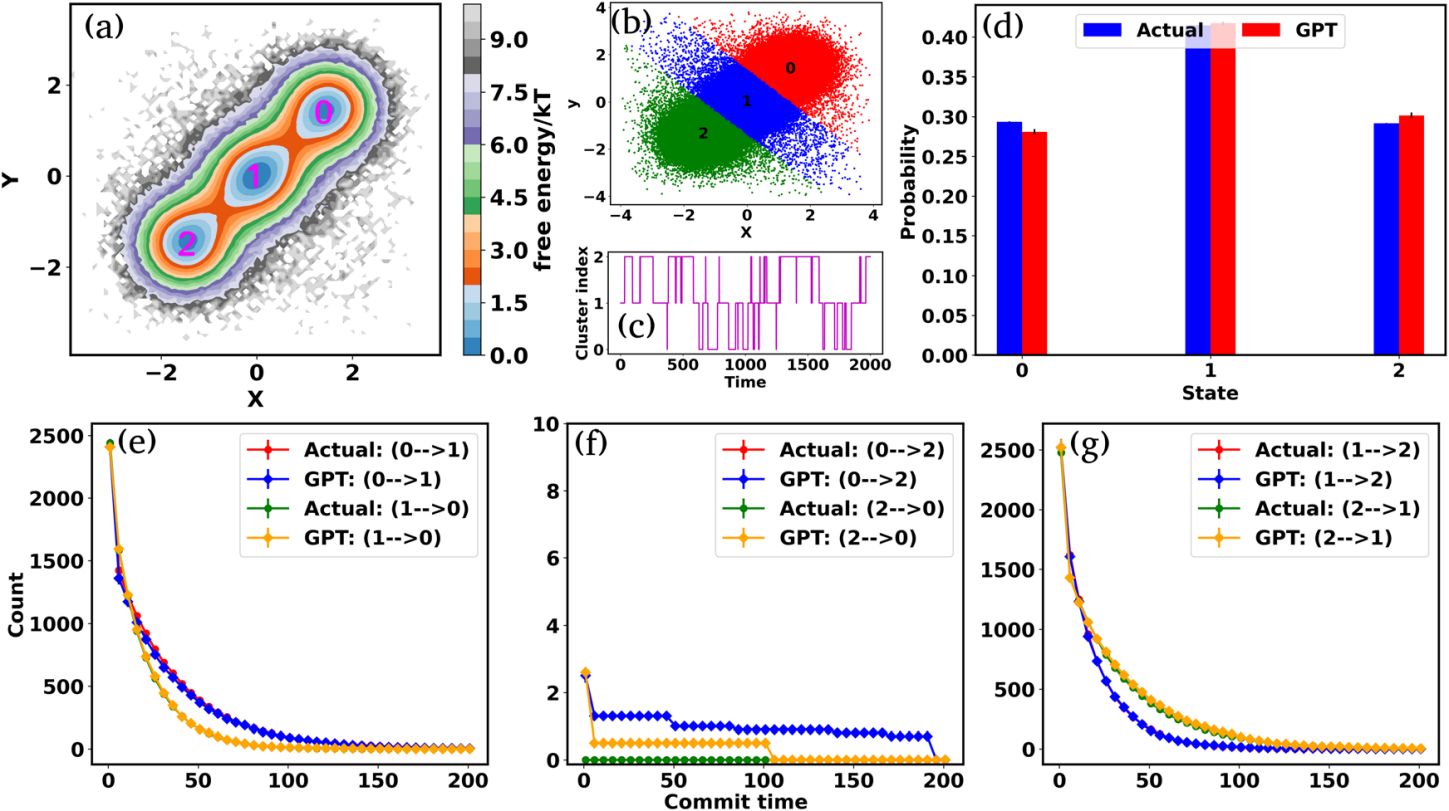
Kinetics and thermodynamics for the toy model system. (a) Free energy surface (FES) plot for the 3-state toy model in its *X* and *Y* coordinate space. The particle can transition from one minimum to the other. (b) Scatter plots of the *X* and *Y* coordinates, with distinct clusters representing metastable states identified through K-means clustering. (c) The trajectory of the particle in the 3-state potential after state decomposition. (d) The comparison of state probabilities between the actual and GPT-generated time series data for the 3-state toy model. The plot highlights the accuracy of the GPT model in predicting the state probabilities. (e-g) Transition counts as a function of commit time for a 3-state toy model. These plots indicate the ability of the GPT model to learn contextual relationships among the states and generate a sequence of states that are kinetically and thermodynamically significant. Here the error bar represents the standard error and the commit time is in units of *τ*_BD_ (see the “Methods”).

To begin, we will delve into two hand-crafted model systems: 2D Brownian dynamics (BD) simulations of a single particle in 3-state and 4-state potentials. The mathematical representations of the potentials and simulation details are provided in the “Methods” section. We employ the BD trajectories to compute the 2D free energy of the two model systems within their *X* and *Y* coordinate space, defined as 
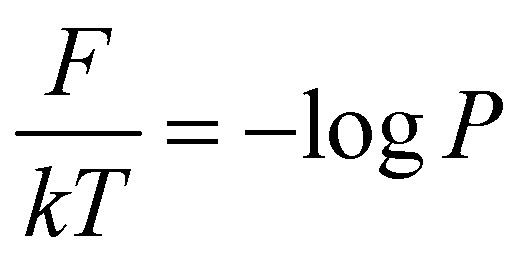
, where *P* is the probability, calculated by using a 2D histogram of the coordinates. Fig. 2(a) represents the free energy surface (FES) plot for the 3-state toy models. The plot exhibits three minima in the FES and the particle can hop from one minimum to another. The states are marked in the plots using magenta color, identified through K-means clustering in coordinate space (Fig. 2(b)). After clustering the data, the entire trajectory is discretized into a specific number of states. Fig. 2(c) shows the trajectory after spatial discretization, where each cluster index corresponds to a metastable state. The trajectory demonstrates that the particle can stay in a particular state for some time and also exhibits transitions between various states. Now, from both the actual (*i.e.* BD-simulated) and GPT-generated time series data, we can compute the probability of each state by counting the occurrences of that particular state and dividing by the total count. For instance, if the count of state 0 is *C*_0_ and the total count of all states is *C*_tot_, then the probability of state 0 is 
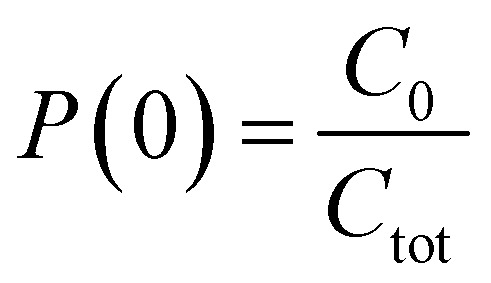
. Fig. 2(d) depicts a comparison between the actual and GPT-generated state probabilities for the 3-state model. The plot suggests that there is a close match between the actual and GPT-generated state probabilities.

To effectively compare the kinetics between actual and GPT-generated time series data, one must analyze the transitions between different states over time. To facilitate this analysis, we utilized the concept of “commitment time or commit time”. This metric represents the duration a particle remains in a given state before transitioning to another.^35,36^ We calculated the total transition count for different commit times, considering all possible state pairs (^*n*^*C*_2_, *n* is the total number of states) and both forward and backward directions. Fig. 2(e–g) represent the transition count as a function of commit time for a 3-state toy model. These plots reveal that the decay of transition counts as a function of commit time is very similar between actual and GPT-generated data in both directions. Furthermore, the 2D FES plot (Fig. 2(a)) indicates that states 0 and 2 are spatially distant. The actual data corroborate this by showing no direct transitions between these states. On the other hand, the GPT model predicts a finite number of transitions between them, although the overall frequency of such transitions remains very small (approximately three), indicating the rare nature of such transitions. This might lead one to believe that the GPT model has perhaps missed to fully capture the spatial disconnection between these states. Alternatively, it could also reflect the model's attempt to account for the statistical possibility—however small and rare—of transitions between distant states within the learned context of the trajectory data.

We assessed the accuracy of this approach for a 4-state toy model system, as depicted in Fig. S2(a–j).† Barring slight deviations in probabilities and a few transitions (Fig. S2(f) and (i)†), the results are very similar for both actual and GPT-generated states. The free energy surface (FES) plot in Fig. S2(a)† indicates that states 1 and 0, as well as states 2 and 3, are spatially distant, with no direct transitions between them in the actual data. Remarkably, the GPT-generated states capture these trends very nicely. However, upon closer examination, the FES (Fig. S2(a)†) suggests that although states 0 and 2, as well as states 1 and 3, are spatially proximate, the trajectory is not sampled properly. This discrepancy may contribute to the slight deviations in transition counting between actual and GPT-generated data for these pairs of states (0–2, 2–0, 3–1, and 1–3). Nevertheless, the results collectively indicate that the GPT model effectively learns the context and relationships between states, enabling it to generate a sequence of states that are both kinetically and thermodynamically significant.

### Predicting the ensemble probabilities and state-to-state transition kinetics in Trp-cage mini protein

**Fig. 3:**
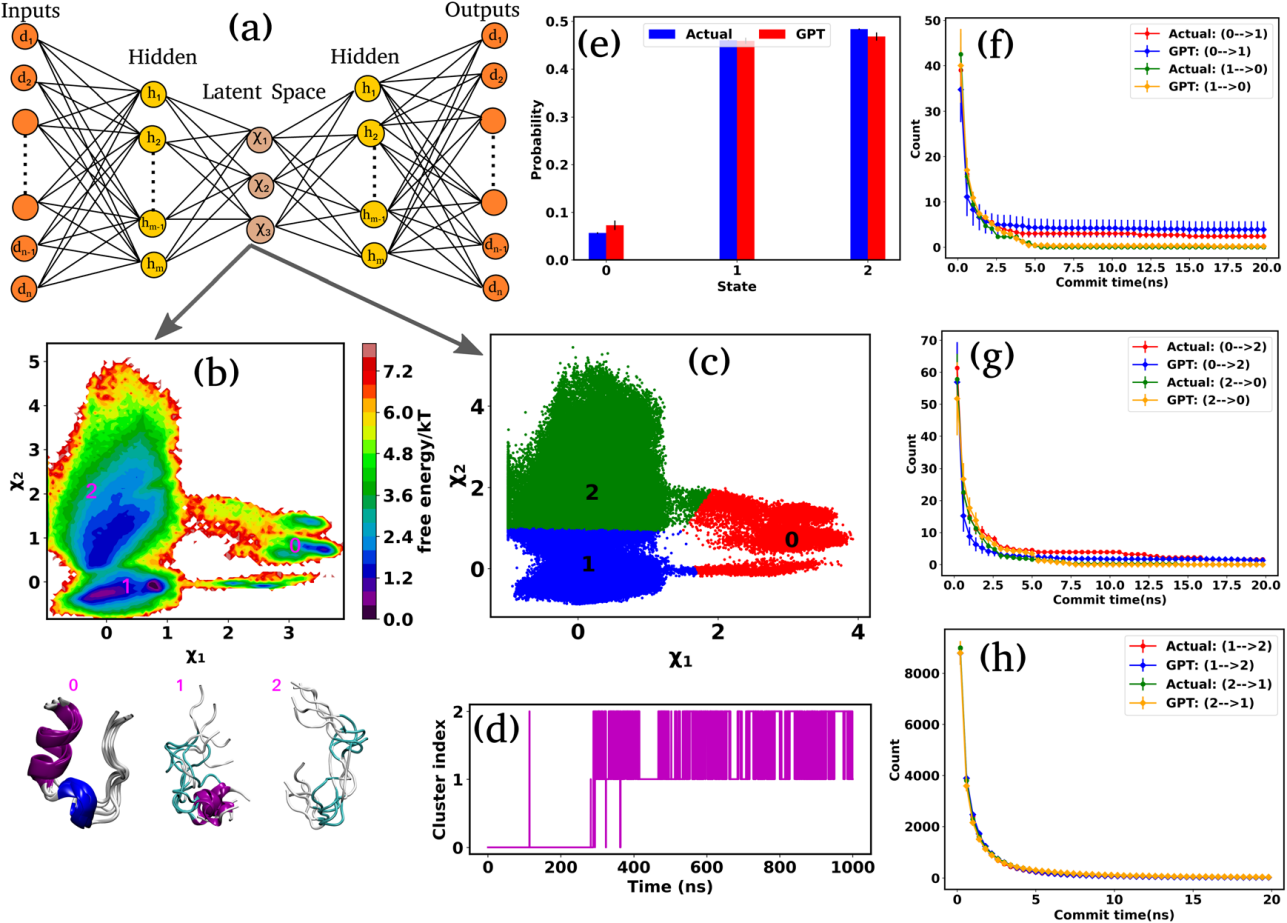
Kinetics and thermodynamics for Trp-cage mini protein. (a) A schematic representation of the Autoencoder. In this setup, *d*_1_, *d*_2_,…, *d*_*n*_ denote the input and output nodes, delineating the dimensions of the input and output data, while *h*_1_, *h*_2_,…, *h*_*n*_ represent the hidden nodes. Furthermore, *χ*_1_,…, *χ*_3_ represent the latent nodes. (b) 2D FES plot along latent space *χ*_1_ and *χ*_2_ for Trp-cage with three distinct minima and extracted conformations. (c) The state decomposition of the MD trajectory is achieved through K-means clustering on the latent space, which divides the entire trajectory into distinct sequences of states. (d) The trajectory of Trp-cage after state decomposition. (e) The comparison of state probabilities between actual and GPT-generated time series data. These plots suggest that the GPT model effectively captures the probabilities with minor deviations. (f–h) Comparison of transition counts between actual and GPT-generated states, showcasing the GPT model's ability to accurately capture state transitions. Here, the error bar represents the standard error.

Encouraged by the promising results in model potential, we extended our approach to a globular 20-residue mini-protein Trp-cage. This biomolecule is known for complex and multi-state conformational ensembles, despite its small size and remains an ideal candidate for experimental and computational studies of protein folding. Towards this end, we utilized a long (100 μs) unbiased simulation trajectory of Trp-cage provided by D. E. Shaw Research.^38,39^ For this system, due to higher degrees of freedom, it is necessary to define suitable order parameters or collective variables (CVs) to discretize the time series data into a certain number of states. Nonetheless, the identification of precise CVs is a challenging problem. To address these challenges, we employ an encoder-decoder-based unsupervised deep neural network called Autoencoder.^16,40,41^ An Autoencoder is a powerful non-linear dimension reduction technique that is used to transform the high-dimensional input data in a lower-dimensional space known as the latent space. The encoder component of the Autoencoder maps the input data to the latent space, while the decoder reverses this process, reconstructing the original input from the latent space. During this process, the model optimizes its weights and biases to preserve the most important information from the input data in the lower-dimensional representation. Fig. 3(a) represents a schematic of Autoencoder architecture, where the distances between *C*_α_ atoms serve as input features. During the training of the Autoencoder, we monitor the fraction of variation explained (FVE) score to determine the optimal dimension of the latent space. A detailed description of the Autoencoder architecture and the various hyperparameters is provided in the “Methods” section and Table S3.

Fig. 3(b) represents the 2D FES plot along the latent space *χ*_1_ and *χ*_2_, obtained from the Autoencoder. The figure clearly shows three distinct minima in the FES plot, indicating distinct conformations. To visualize different conformations, we extracted a few conformations near each minimum in the FES and overlaid them. The superimposed conformations reveal mainly three metastable states: folded α-helix (state-0), partially folded α-helix (state-1), and unfolded random coils (state-2). After clustering in the latent space, the entire trajectory comprises metastable states and their transitions, as depicted in Fig. 3(c). Fig. 3(d) illustrates the discretized trajectory, with the majority of transitions occurring between states 1 and 2. Fig. 3(e) represents a comparison of the state probabilities between the actual and GPT-generated time series data for the Trp-cage protein. These figures demonstrate that the GPT model has effectively captured the probabilities, with minor deviations observed for a few states. Importantly, these deviations are within the error bars.

Next, to probe the kinetics between various states of the Trp-cage protein, we calculated the transition counts as a function of commit time, akin to methodologies employed for 3-state and 4-state toy models. Fig. 3(f–h) compare the transition counts between actual and GPT-generated states for the Trp-cage protein. These figures encompass all possible pairs and transitions in both forward and reverse directions. The results indicate that the GPT model accurately captures the transitions between states. Quite interestingly, the GPT model can also predict the highest number of transitions between states 1 and 2 accurately, which aligns with our observations from the trajectory itself (Fig. 3(e)). In summary, together these results suggest that the GPT model effectively captures the probability and the transition counts between various states of the Trp-cage protein, providing valuable insights into the kinetics and thermodynamics of these systems.

### GPT flourishes in kinetic prediction of state sequences of complex intrinsically disordered protein

Next, we turn our attention to an intricately complex system: α-synuclein. It is a prototypical intrinsically disordered protein (IDP) that predominantly resides in the human brain, especially nerve cells.^42^ It plays a crucial role in the communication between these cells by releasing neurotransmitters, which are messenger chemicals.^43^ However, the excessive accumulation of these proteins can lead to several neurodegenerative disorders such as Parkinson's disease and other synucleinopathies.^44–46^ For α-synuclein, we utilize the 73 μs trajectory provided by D. E. Shaw Research (DESRES).^38,39^

**Fig. 4:**
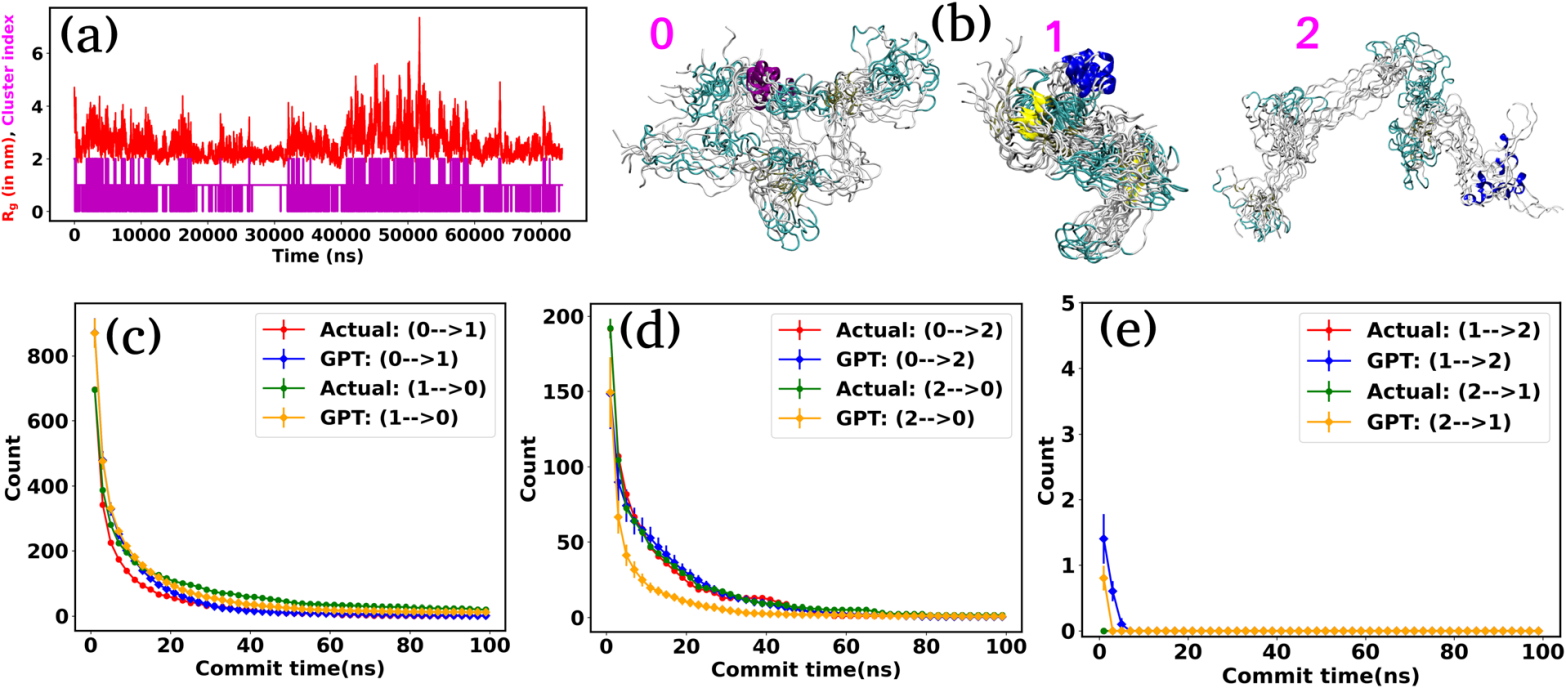
The radius of gyration (*R*_g_) and transition count comparison for α-synuclein. (a) The (*R*_g_) as a function of time for α-synuclein (red plot). The magenta color plot represents the trajectory after state decomposition *via* K-means clustering. (b) The diverse conformational states are identified as intermediated compact (state-0), collapsed (state-1), and extended (state-2). (c–e) Transition count comparison between the actual and GPT-generated time series data as a function of commit time for α-synuclein. The transition dynamics fairly match with actual and GPT-generated data, except at a smaller commit time. Here the error bar represents the standard error.

We used the radius of gyration (*R*_g_) as CVs to decompose the entire trajectory into a specific number of states. Fig. 4(a) represents the (*R*_g_) (red color) of α-synuclein as a function of time, showcasing the protein's diverse conformational possibilities. Through K-means clustering in the *R*_g_ space,^47^ we discretize the total trajectories into distinct states (Fig. 4(a) magenta color plot). Specifically, three states have been identified: intermediated compact (state-0), collapsed (state-1), and extended (state-2), with a superimposition of snapshots revealing significant conformational heterogeneity within each state (Fig. 4(b)).

Fig. S3† compares the state probabilities between actual and GPT-generated time series data for α-synuclein. The figure clearly shows the deviation in the state probability values. Next, to analyze the transition dynamics, we have calculated the transition count of each state. Fig. 4(d–f) depict the comparison of transition counts as a function of commit time between actual and GPT-generated time series data for α-synuclein. While there are some deviations in probability values, the transition dynamics align fairly well with actual and GPT-generated data, except for a smaller commit time. Specifically, the GPT model generates a lower count for the transition (2 → 0) compared to actual data. The deviations in state probability and transition dynamics indicate the intricacies involved in accurately predicting the dynamical behavior of such a complex system.

### Assessing the transformer's predictive ability in a far-from-equilibrium system

In the preceding sections, we have mainly focused on capturing the kinetics and thermodynamics of various models and real systems that are in thermodynamic equilibrium. Finally, in this section, we shift our attention to an active system. Most living organisms are active and their movement is powered by energy consumption from internal or external sources. The continuous consumption and dissipation of energy drive these systems far from equilibrium. Notably, the activity or self-propulsion force plays a crucial role in the formation of many self-organized collective phenomena such as pattern formation,^48–51^ motility-induced phase separation,^52–55^ swarming motion,^56–59^*etc.* In this study, we employ a model system, an active worm-like polymer chain,^60^ where the activity or self-propulsion force acts tangentially along all bonds. We have utilized our in-house BD simulation trajectory to study the active polymer chain (see the Methods for details).

**Fig. 5:**
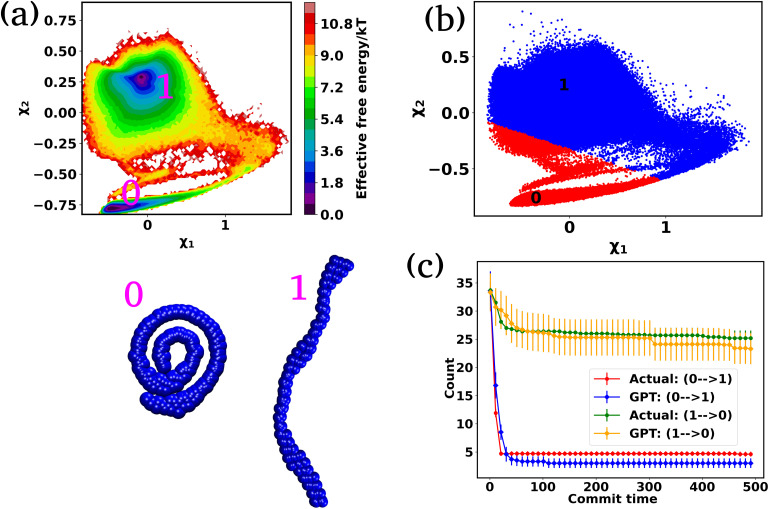
The effective free energy surface and transition count comparison for the active worm-like polymer chain. (a) The effective FES plot of the active polymer chain across the latent space *χ*_1_ and *χ*_2_. This plot highlights two metastable states: a bending state (state-1) and a spiral state (state-0). (b) The latent space has been clustered *via* K-means clustering to discretize the trajectory. (c) The comparison of transition counts between actual and GPT-generated data for the active polymer chain, reflecting the system's long stay at particular states before transition and the violation of detailed balance. The GPT model accurately generates a kinetic sequence of states, maintaining saturation and detailed balance violation, albeit with some small deviation from actual data. Here the error bar represents the standard error and the commit time is in units of *τ*_BD_ (see the “Methods”).

After training the Autoencoder by using inter-bead distances as features, we have chosen two-dimensional latent space as CVs. For active systems, the quantity 
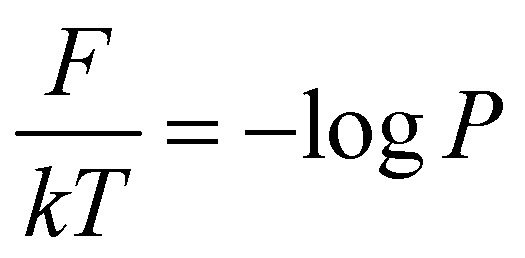
 may not correspond to the free energy in the same way as in a passive description. To avoid confusion, we refer to it as the effective free energy. Fig. 5(a) shows the effective FES plot across the latent space *χ*_1_ and *χ*_2_ for the active polymer chain and the corresponding metastable states are highlighted in magenta color. The overlaid plots suggest that there are mainly two metastable states: a bending state (state-1) and a spiral state (state-0). Notably, despite the apparent simplicity of the two states, the visualization of the trajectory (Fig. S4(a)†) suggests that the system spends a very long time within each state along with spontaneous spiral formation and breakup occurrences. Subsequently, we employ K-means clustering on the latent space derived from the Autoencoder to discretize the trajectory (Fig. 5(b)).

Fig. S4(b)† compares the state probabilities between actual and GPT-generated time series data for the active polymer chain. The comparison reveals the deviations in the state probability. Fig. 5(c) depicts the comparison of transition counts as a function of commit time between actual and GPT-generated time series data. However, interestingly, the GPT model accurately generates the transition for the active system. As mentioned earlier, for the active polymer chain, the system stays at a particular state for a long time before transitioning to the other state. This behaviour is reflected in the plots, where both curves saturate after a certain commit time. Moreover, the forward and backward transition curves suggest a violation of detailed balance, which is an inherent feature of active systems that are far from thermodynamic equilibrium. Remarkably, the GPT model successfully generates a sequence of states that maintain the saturation nature as well as the violation of detailed balance, albeit with some deviation from actual data within the error bar. These findings indicate that the GPT model is very powerful for future state predictions, even in complex active systems. In a similar spirit, for comparison purposes, we have also computed similar metrics for a ‘passive’ polymer chain for enhanced clarity (Fig. S5†). Here too, we observed a strong alignment between the kinetics and thermodynamics captured by the GPT model and the actual BD-generated data.

### Deciphering the inner workings of GPT's prediction accuracy of a kinetic sequence of states

In the previous sections, our focus has been on exploring the thermodynamics and kinetics of diverse model and real systems, whether in thermodynamic equilibrium or out of equilibrium. We have observed that despite variations in state probabilities, the GPT model generates a sequence of states that accurately maintain transition dynamics in a statistical sense. Now, in this section, we delve into identifying the pivotal factors that maintain these precise transition dynamics.

**Fig. 6:**
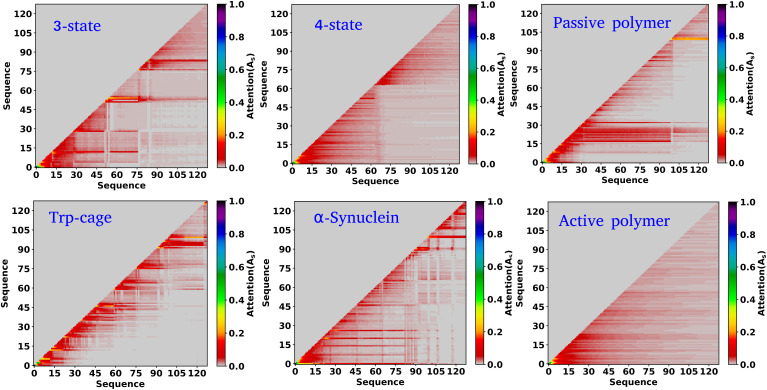
Attention score for all the systems analyzed. The heat map of the attention score computed from the multi-head attention layer of a trained GPT model, for six distinct systems under consideration. These scores reveal that there is finite attention among various tokens and highlight the evidence of long-range attention.

In the field of natural language processing (NLP), the seminal work by Vaswani *et al.* titled “Attention Is All You Need”^33^ introduces the paradigm-shifting concept of self-attention. The state-of-the-art transformer model, based on attention mechanisms, has demonstrated superior performance compared to traditional recurrent neural networks (RNNs). To understand the role of attention, we computed the attention score from the multi-head attention layer, which is defined as 
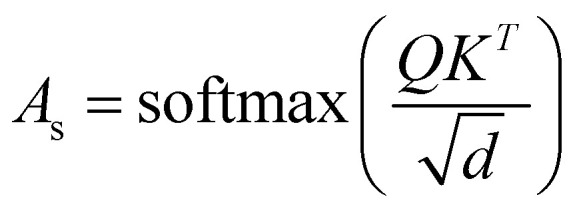
 (see eqn (3) for details), where *Q*, *K*, and *d* are the query, the key, and the dimension of the embedding layer, respectively. In our study, we randomly selected 20 chunks of sequence length 128 from GPT-generated time series data and fed them as inputs for the trained GPT model. Subsequently, we computed the attention scores from each head, averaging them across all heads and the 20 random chunks. Physically, these attention scores unveil correlations among different tokens within a sequence of time series data. Fig. 6 show the heat map of the masked attention score for all the systems. These plots highlight the presence of significant finite, non-zero attention among various tokens within sequences. Notably, some tokens exhibit clear evidence of long-range attention, underscoring the model's ability to understand the relationships between events that are far apart.

**Fig. 7:**
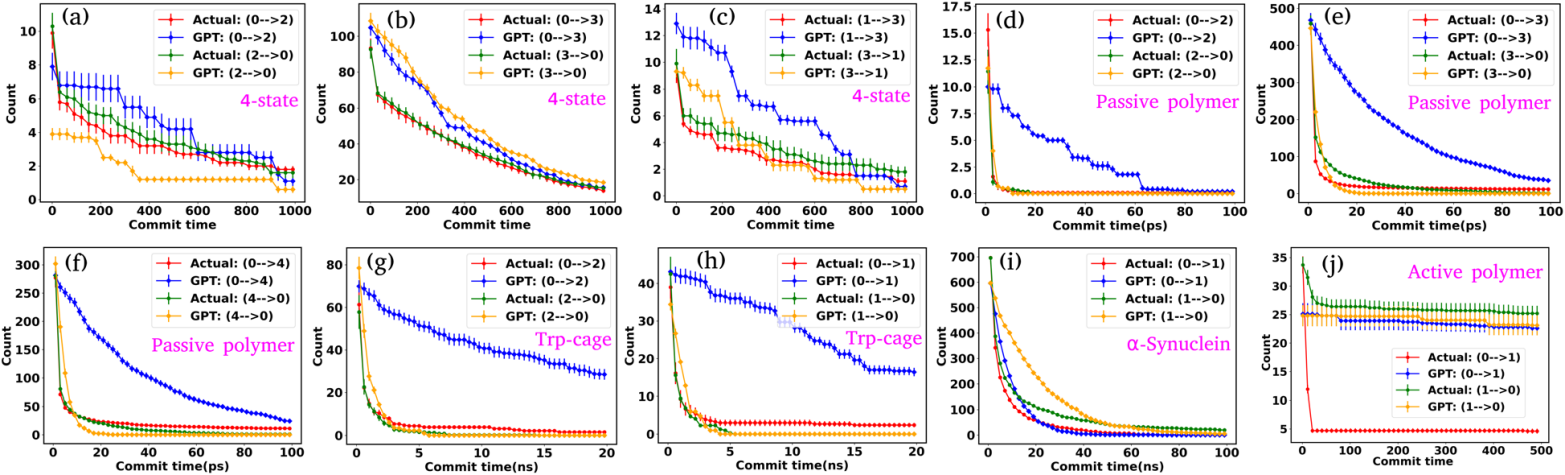
Impact of attention on transition dynamics in the GPT model. (a–j) The comparison of transition counts over commit time between the actual and GPT-generated time series data for all of the systems in the absence of attention mechanisms. There are significant deviations in GPT-generated transition dynamics compared to the actual data. While the GPT model can sometimes accurately predict transitions in one direction, it frequently mispredicts transitions in another direction.

Based on our examination of attention scores, we now pose the following question: how does attention impact the transition dynamics between different states? To address this, we trained the GPT model on all six systems by removing the multi-head attention layer, while maintaining other hyperparameters consistent with our previous model architecture. This approach ensures that the model can no longer capture any short- or long-range correlations between tokens. Our findings indicate that for simple systems, such as a 3-state toy model, the transition dynamics captured by the GPT model remain similar regardless of the presence or absence of the attention layer (see Fig. S6†). This suggests that attention may not play a significant role in these simple systems. However, for other systems, there are substantial deviations in GPT-generated transition dynamics compared to actual data. Fig. 7(a–j) show the comparison of transition counts as a function of commit time between the actual and GPT-generated time series data for all of the systems under consideration as highlighted in the text of each plot. These plots suggest that while in many cases the GPT model can recover the transition counts in one direction, it completely predicts the wrong transitions in other directions in the absence of attention. Here, we have only shown the plots where the deviations are prominent. The comprehensive plots for all the systems with all possible transitions are given in the ESI (Fig. S7–S10†). Together these analyses identify the power of the attention layer. Even in physicochemical systems, attention is crucial for learning the context and relationships or correlations among various tokens of time series data, enabling the model to generate a physically meaningful kinetic sequence of states.

### Performance comparison: MD simulation *vs.* GPT state generation

After training the GPT model, the generation of the sequence of states is extremely fast compared to conventional MD simulations of equivalent systems. For example, the model can generate 20 000 subsequent sequences for α-synuclein within ∼60 minutes, corresponding to a 20 μs simulation of this system with a data saving frequency of 1 ns. Similarly, the model can generate 100 000 sequences for the Trp-cage mini protein within ∼32 minutes, equivalent to 20 μs simulation with a data dumping frequency of 200 ps. As mentioned earlier, we have analyzed six systems; among them the Trp-cage mini protein and α-synuclein are particularly relevant in biophysical contexts, and their simulations were conducted in real-time units. Thus, our primary focus here is to compare the performance of these two systems. To compare this generation's efficiency against actual MD simulation times, we conducted 10 ns simulations for these systems, maintaining all parameters such as box size, salt concentration, temperature, and time step as per the study by Robustelli *et al.*^38^ As the data saving frequency for these two systems is different, we define a quantity 
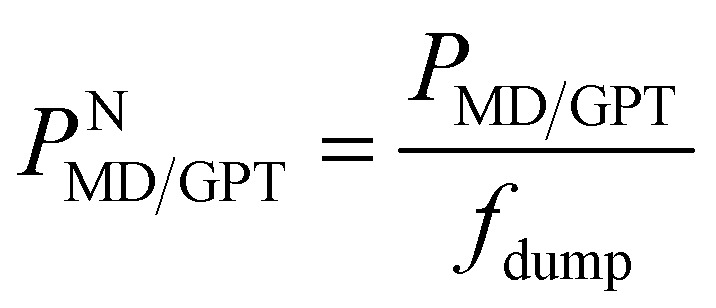
, where *P*_MD/GPT_ is the performance of the MD or GPT model and *f*_dump_ is the saving frequency of the data. This metric can normalize the performance of each system by its respective data saving frequency. All the MD simulations and training of the GPT models were performed on an Intel(R) Xeon(R) Platinum 8352Y CPU at 2.20 GHz, along with an NVIDIA RTX A6000 GPU. Tables 1 and S4† show all of the details of the performance and memory usage of the MD simulations as well as GPT state generation. Table 1 suggests that the performance and normalized performance of the GPT model surpass those of traditional MD simulations, demonstrating its efficiency in generating the kinetic sequence of states of the systems.

**Table 1:** The comparison of the performance between MD simulation and GPT state generation

System	*f*_dump_	*P*_MD_ (ns per day)	*P* ^N^_MD_ (per day)	Training time (minutes)	Gen. sequence	Gen. time (minutes)	*P*_GPT_ (μs per day)	*P* ^N^_GPT_ (10^3^ per day)
α-Synuclein	1.0 ns	∼35.0	∼35.0	∼127.0	20 000	∼6.0	∼4800.0	∼4800.0
Trp-cage	200 ps	∼832.0	∼4160.0	∼35.0	100 000	∼32.0	∼900.0	∼4500.0

What should be the typical size of training data? To evaluate the impact of training data size on our model's ability to predict a kinetic sequence of states in a biophysical system, we trained our GPT model using varying amounts of data. We selected the Trp-cage mini protein, which provides 500 000 frames, in contrast to the 73 124 frames available for α-synuclein (see Table S1†). We generated the same number of states as depicted in Fig. 3 but varied the training data size. Initially, the model was trained with 60% of the total data. We have now conducted additional training with 10%, 40%, and 50% of the data. Fig. S11(a–i) and (j–l)† show the transition counts over time and state probabilities for these different training data percentages. These figures indicate a significant alignment between the actual and GPT-generated data after utilizing 40% of the training data. This suggests that a sufficient amount of training data is crucial for the model to predict a kinetic sequence of states accurately. However, the transition count plots demonstrate that even with just 10% of the training data, the model can still capture complex relationships between various states, generating a kinetic sequence of states that are not entirely erroneous.

In many machine learning tasks, performance improves with training data size up to a saturation point. A similar approach can be applied to a new system by incrementally increasing the dataset size and monitoring the convergence of performance metrics. More complex systems, particularly those with numerous metastable states or intricate free energy landscapes, may require larger datasets to capture transition dynamics effectively. One practical approach is to test whether kinetic properties, such as state transition probabilities, are stable across different dataset sizes.

### GPT outperforms precedent baseline approaches in kinetic sequence generation

In the previous sections, we explored the capabilities of the GPT model in capturing the state-to-state transition dynamics of various systems. In this section, we focus on a detailed comparison between the GPT model and two established approaches: Long Short-Term Memory (LSTM)^34^ networks and MSM.^1–3^ Both models were trained using the same clustered trajectories as the GPT model. The MSM was originally developed for equilibrium systems, relying on the assumption of the Markovian properties of the system. This means that the transition probabilities in the MSM are calculated under the conditions that the system satisfies detailed balance, which is a key requirement for equilibrium systems. However, for active systems, this assumption is not valid, as such systems inherently violate detailed balance due to their non-equilibrium nature. As a result, using the MSM to analyze an active system will lead to incorrect kinetic information. In our study, we used the MSM to compare its results with those of the GPT model. We also observed that the MSM provides incorrect results for the active system, as expected.

**Fig. 8:**
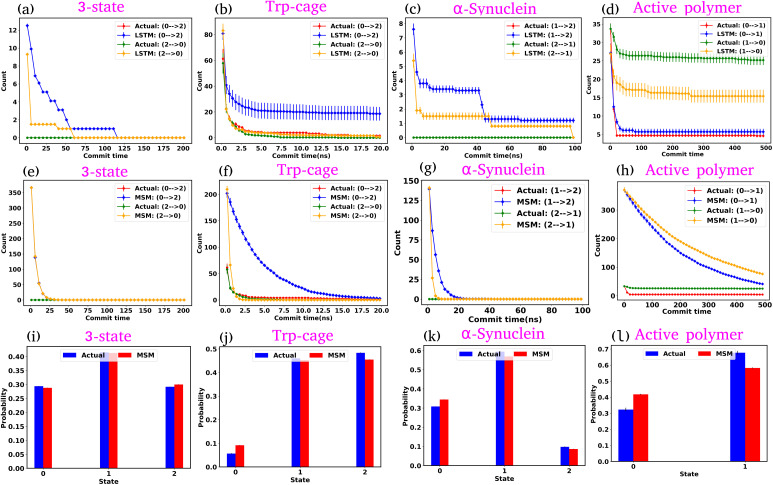
Comparison of state-to-state transition dynamics in LSTM and MSM models. (a–d) State-to-state transition counts as a function of commit time between actual data and LSTM-generated time series data for various systems. (e–h) Transition counts over commit time between actual data and MSM-generated time series data for various systems. The MSM shows consistent deviations across all systems, failing to capture the correct sequence of state transitions. (i–l) Comparison of state probabilities between actual and MSM-generated data for various systems. Although the MSM struggles with dynamic predictions, it aligns well with actual data in terms of state probabilities.

We first examine the LSTM networks, which are known for their ability to model sequential data by capturing long-range dependencies. To ensure consistency, we used the same embedding dimension for the input data as with the GPT model, while all other hyperparameters are detailed in Table S5. We observed that in most of the cases, the LSTM captures the state-to-state transition accurately for various systems under consideration. However, in a few cases, there are significant deviations in the state-to-state transition from the actual data. Fig. 8(a–d) show the transition count as a function of commit time for various systems as highlighted in the title of each plot. Here, we have highlighted only the plots where the deviations are most significant. For a comprehensive overview, all other plots for each system, including all possible transitions, can be found in the ESI (Fig. S12–S14†). Since the GPT model uses the self-attention mechanism, it likely enhances its ability to capture long-range dependencies more effectively, enabling it to generate a more accurate kinetic sequence of states compared to the LSTM model.

Next, we constructed Markov State Models (MSMs), a powerful framework for analyzing the kinetics of molecular systems by discretizing the state space into metastable states. The first step in building an MSM is selecting an appropriate lag time for calculating the transition probability matrix, ensuring that the model behaves in a Markovian manner. For all systems, the lag time was chosen based on the implied time scales (ITSs) or relaxation time scale plots as a function of lag time (Fig. S15 (a–d)†). We selected the lag time where the ITS plots approximately level off, denoted by a vertical magenta line. Subsequently, a Chapman–Kolmogorov test was performed to further verify the Markovianity of the model (Fig. S16–S19†). After constructing the MSM at the chosen lag time, we generated a kinetic sequence of states using the transition probabilities of the previous states. Fig. 8(e–h) represent the state-to-state transition counts as a function of commit time across various systems. These plots suggest significant deviations between the actual data and the MSM-generated kinetic sequence of states for all systems. While we have highlighted a few transitions here, all other possible transitions are presented in the ESI (Fig. S20–S22†), where similar deviations are observed. However, quite interestingly, the MSM-generated state probabilities align well with the actual data (Fig. 8(i–l)). Together, these observations suggest that while the MSM accurately predicts the thermodynamics of the systems, it fails to correctly capture the temporal sequence of states (dynamics). To gain deeper insights, we built the MSM with a lag time of one step (the data dumping frequency), regardless of whether the ITS curve had plateaued for all systems. Fig. S23–S26† depict the transition counts as a function of commit time for the systems under consideration. While the MSM accurately generates a kinetic sequence of states for simpler systems, such as a 3-state model, it fails to match the state-to-state transition counts with the actual data for more complex systems, particularly the Trp-cage mini protein and active systems.

Based on these observations, we conclude that our GPT model outperforms traditional methods like the MSM in generating future states in the correct sequential order. The MSM requires the assumption of detailed balance for transition matrix calculation and the selection of an appropriate lag time to ensure Markovian behavior. In contrast, the GPT model does not rely on the Markov properties of the system and can generate a kinetic sequence of states with the same temporal precision as its training data, regardless of any intrinsic time scale (such as the lag time required by the MSM) it learns from the system.

In our previous analyses, we discretized the trajectory into a few states based on the minima in the free energy surface (FES). Now, we have discretized the trajectory into a larger set of states, effectively fine-graining the FES. Our focus is on two systems: the Trp-cage mini protein and α-synuclein. We clustered the data into 20 clusters along their collective variables (CVs). Using the same protocol as before, we trained both the GPT model and MSM on this clustered data. After training, we generated a kinetic sequence of states using both models and compared the results. The lag time selection for the MSM was determined based on the approximate plateau observed in the ITS plots (Fig. S27†).

**Fig. 9:**
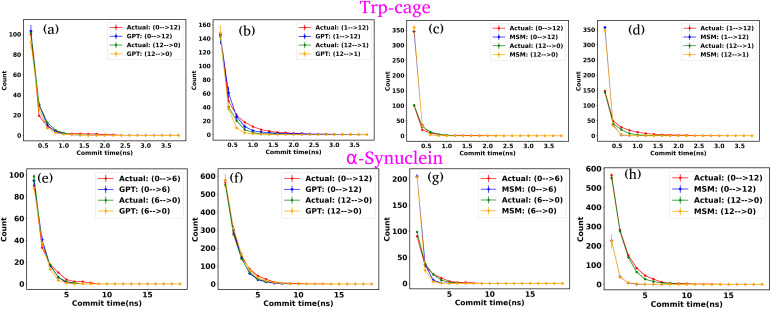
Comparison of state-to-state transition dynamics for Trp-cage mini protein and α-synuclein using GPT and MSM models. (a)–(d) State-to-state transition counts as a function of commit time for the Trp-cage mini protein, derived from the GPT model and MSM, respectively. (e)–(h) State-to-state transition counts as a function of commit time for α-synuclein, obtained from the GPT model and MSM, respectively. These results demonstrate that the GPT model more accurately predicts the kinetic sequence of states compared to the traditional MSM for both systems.

Fig. 9(a)–(d) depict the state-to-state transition count as a function of commit time for the Trp-cage mini protein, derived from the GPT model and MSM, respectively. Similarly, Fig. 9(e)–(h) represent the state-to-state transition count as a function of commit time for α-synuclein, obtained from the GPT model and MSM, respectively. These plots suggest that, even with a larger number of clusters, the GPT model does a better job than the traditional MSM in accurately predicting the kinetic sequence of states for both systems. It is important to note that with 20 states, there are now 190 (^20^*C*_2_ = 190) possible transitions. In this representation, we have only shown the two transitions with the highest transition counts. The complete set of all possible transitions is provided in the ESI (Fig. S28–S47†).

We now aim to reconstruct the one-dimensional free energy plots using the GPT model. For α-synuclein, we used the radius of gyration (*R*_g_) as the one-dimensional CV to discretize the trajectory into specific states. For this free energy calculation, we focused solely on α-synuclein, discretizing the trajectory by binning along the CV as proposed by Tsai *et al.*^35^ Fig. S48† compares the GPT-generated one-dimensional free energy plot with the actual data along *R*_g_. The comparison reveals a strong agreement between the actual and GPT-generated free energy plots.

## Discussions

The time evolution of biophysical systems undergoes various conformation changes, depending on environmental conditions. Understanding these dynamics typically requires experiments or Molecular Dynamics (MD) simulations. However, comprehending the long-term behaviour of these systems requires running computationally expensive simulations. In this study, we present a comprehensive approach, employing state-of-the-art machine learning models, particularly decoder-only transformers, to predict the kinetic sequence of states of physicochemical systems. Through an extensive analysis of the MD trajectory of various models and real systems, we have demonstrated the efficacy of the GPT model in capturing both the kinetics and thermodynamics of these systems.

Our study began with simplified model systems, namely 3-state and 4-state toy models. We employed K-means clustering on coordinate space for this simple system to discretize the trajectory. Subsequently, we delved into more complex systems such as the Trp-cage mini protein, 32-bead passive polymer chain, and intrinsically disordered protein α-synuclein. However, for these systems, we utilized another ML-based technique, Autoencoder, to identify the relevant collective variables for discretization. Our results highlight the ability of the GPT model to accurately predict the probabilities of different states and capture the transition dynamics between them. Furthermore, we extended our analysis to include an active system, a 32-bead active worm-like polymer chain, where the system is far from thermodynamic equilibrium. Remarkably, the GPT model successfully predicted the kinetics and thermodynamics of the active system.

A key aspect of our study is the ability of the GPT model to capture and reconstruct complex transition dynamics in molecular systems, offering valuable chemical and physical insights beyond traditional kinetic modeling approaches. One of the most striking findings is the role of the attention mechanism in preserving long-range dependencies within kinetic sequences. Through attention score analysis, we observed significant correlations between distant states, highlighting the model's ability to recognize intricate transition patterns that may be obscured in traditional MSMs. Furthermore, by removing the attention layer from the model, we observed substantial deviations in transition dynamics for complex systems. Therefore, these findings strongly suggest that the attention mechanism plays a pivotal role in maintaining accurate predictions.

While our model does not predict entirely new states beyond the training data, its ability to generate statistically precise kinetic sequences provides valuable insight into transition dynamics, helping to reconstruct long-timescale behavior from limited MD trajectories. By leveraging learned transition patterns through the attention mechanism, the model can rapidly generate statistically robust kinetic pathways, enabling accurate estimations of state-to-state transition probabilities, especially for complex systems and active systems, where the traditional MSM-based model failed to accurately predict the kinetic sequence of future states. The attention maps highlight how the model internally focuses on long-range temporal relationships in the trajectory. This “mechanistic introspection” offers a data-driven window into how conformational history affects future evolution—a level of mechanistic interpretability not directly accessible from traditional MD or even MSMs.

Although the transformer-based large language models (LLMs) were specially developed for tasks like machine translation and natural language processing, our study demonstrates their effectiveness in predicting the kinetics and thermodynamics of a diverse array of biophysical systems. One notable limitation of our GPT model is that it never generates new states beyond its training data. In terms of language, the transformer-based model is always unable to generate new vocabulary. Nonetheless, the model can learn the complex syntactic and semantic relationships present in the sequence of tokens, which help them to generate a kinetic sequence of states of the system very correctly. If some system shows a completely new state at a very long time that is not present in the training sequence, the model cannot generate that particular state. This suggests that one needs very good MD sampling data for a physical system to predict the kinetic sequence of states of the system. Another concern is the large amount of MD simulation data required to effectively train the GPT model. While we have demonstrated fairly accurate state probabilities using only 10% of the simulation data for Trp-Cage, this training data included multiple back-and-forth transitions between metastable states. However, recent advances in ML have introduced innovative techniques such as variational autoencoders (VAEs),^61–63^ generative adversarial networks (GANs),^64,65^ diffusion models,^66–68^*etc.*, which can be utilized for generating new conformations and improving sampling in biophysical systems.^69–71^ While these techniques enhance sampling quality, they may lack the temporal information crucial for understanding the dynamics of the system. In conclusion, our findings highlight the potential of LLMs as powerful tools for advancing our understanding of biophysical systems, offering new avenues for further exploration and improvement in this field. In all our analyses, we have relied on unbiased MD data to predict the kinetic sequence of states of various physicochemical systems. In the future, it would be logical and interesting to extend our model to incorporate biased enhanced sampling simulations for learning the long-timescale behavior in molecular dynamics.^72^

## Methods

### 3-state and 4-state toy models

To simulate a particle in a 2D 3-state and 4-state potential well, we adopted the same functional form for the potential as Tsai *et al.*^35^ The potential for the 3-state model is given by

5 *V*_3_(*x*, *y*) = *W*_3_(*x*^6^ + *y*^6^) − *G*(*x*, *x*_1_)*G*(*y*, *y*_1_) − *G*(*x*, *x*_2_)*G*(*y*, *y*_2_) − *G*(*x*, *x*_3_)*G*(*y*, *y*_3_)

Similarly, the potential for the 4-state model is given by

6 *V*_4_(*x*, *y*) = *W*_4_(*x*^4^ + *y*^4^) − *G*(*x*, *x*_1_)*G*(*y*, *y*_1_) − *G*(*x*, *x*_2_)*G*(*y*, *y*_2_) − *G*(*x*, *x*_3_)*G*(*y*, *y*_3_) − *G*(*x*, *x*_4_)*G*(*y*, *y*_4_) − *G*(*x*, *x*_5_)*G*(*y*, *y*_5_)

where *G* is a Gaussian function as 
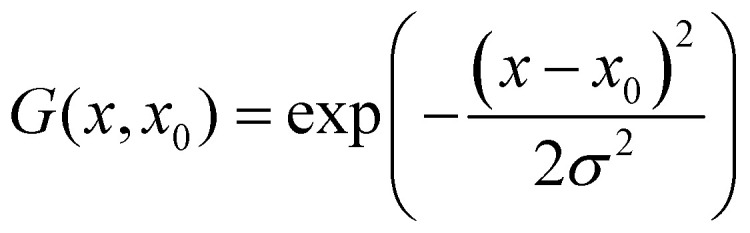
. Here, *x*_0_ and *σ* are the mean and standard deviation of the distribution. In our simulation, we kept *W*_3_ = *W*_4_ = 0.0001 and *σ* = 0.8. The mean values of Gaussian distribution for the 3-state model are given by *x*_1_ = 0.0, *y*_1_ = 0.0, *x*_2_ = −1.5, *y*_2_ = −1.5, and *x*_3_ = 1.5, *y*_3_ = 1.5. Similarly, for the 4-state model, these values are *x*_1_ = 0.0, *y*_1_ = 0.0, *x*_2_ = 2.0, *y*_2_ = −1.0, *x*_3_ = 0.5, *y*_3_ = 2.0, *x*_4_ = −0.5, *y*_4_ = −2.0, and *x*_5_ = −2.0, *y*_5_ = 1.0. We performed Brownian dynamics simulations for these two systems by integrating the equation of motion:7
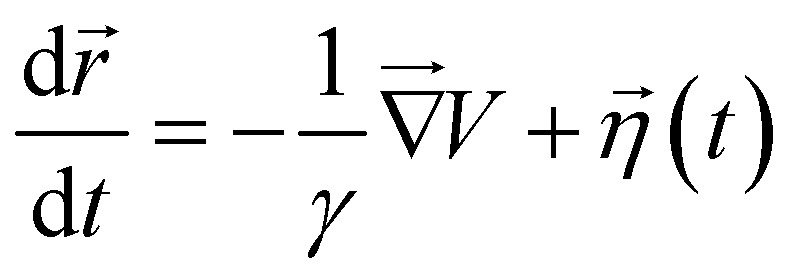
where *γ* is the friction coefficient, *V* is the potential energy, and *η⃗*(*t*) is a random noise, satisfying the fluctuation–dissipation theorem, *i.e.* 〈*η⃗*(*t*)·*η⃗*(*t′*)〉 = 4*kTγ*^−1^*δ*(*t* − *t′*). Here, all simulation times are in units of 
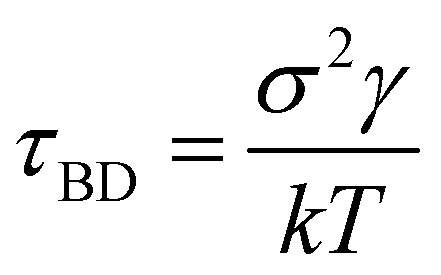
, where *σ* = 1 is the diameter of the particle. Integration of eqn (7) was performed using the Euler method with a time step δ*t* = 0.01*τ*_BD_ by setting 
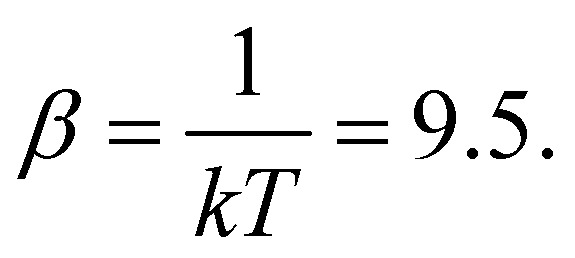


### Passive polymer chain

We performed a very long molecular dynamics simulation (5.74 μs) for a passive polymer chain, with the model and simulation parameters of the polymer system detailed in a previous study by our group.^73^

### Trp-cage mini protein and α-synuclein

For Trp-cage and α-synuclein, we utilized very long molecular dynamics simulation trajectories from D. E. Shaw Research.^38^ The Trp-cage trajectory spanned 100 μs, while the α-synuclein trajectory was 73 μs long. These simulations were performed using the a99SB-disp force field on Anton specialized hardware.^39^ The detailed simulation protocols can be found in the original paper by Robustelli *et al.*^38^ However, in the original paper, the trajectory of α-synuclein was 30 μs long. Due to some periodic image issues in the original trajectory, the authors provided the extended 73 μs trajectory for α-synuclein, maintaining the same simulation setup as before but increasing the box size.

### Active worm-like polymer chain

The two-dimensional worm-like polymer chain consists of *N* = 32 beads connected by a stiff spring with spring constant *k*_0_. Each bead has a diameter of *σ* and an equilibrium bond length of *d*_0_. The dynamics of the polymer chain are governed by overdamped motion, described by the equation8
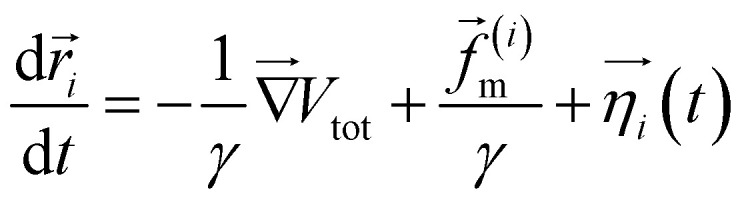
where *γ* is the friction coefficient and *V*_tot_ is the total potential energy of the system, which includes bonding potential *V*_bond_, bending potential *V*_bend_, and non-bonded potential *V*_nb_. The bonding potential *V*_bond_ is given by9
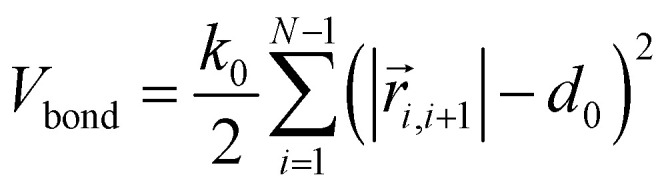
The bending potential *V*_bend_ is given by10
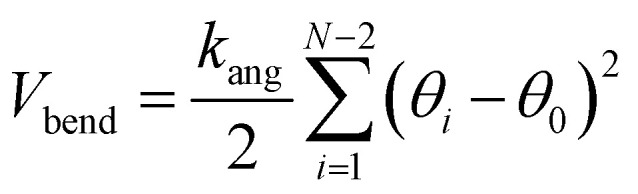
where *k*_ang_ is the bending rigidity and *θ*_0_ is the equilibrium bond angle. The non-bonded potential *V*_nb_ is taken as the Hertzian potential:11
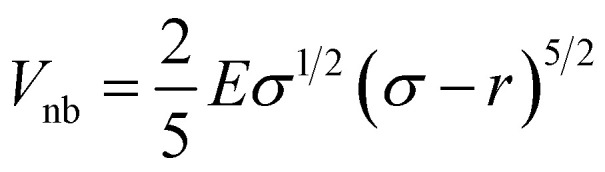
These potentials are very generic for the simulation of any passive polymer chain. However, the most important force for an active polymer chain is the motility or self-propulsion force. Here, *f⃑*_m_ represents the self-propulsion force, which acts tangentially along all bonds. The random noise *η⃗*_*i*_(*t′*) satisfies the fluctuation–dissipation theorem, *i.e.* 〈*η⃗*_*i*_(*t*)·*η⃗*_*j*_(*t′*)〉 = 4*kTγ*^−1^*δ*_*i*,*j*_*δ*(*t* − *t′*). All lengths and energies are in units of *σ* and *kT*, respectively, and the simulation time is in units of 
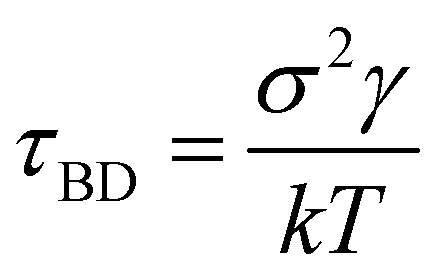
. The Brownian dynamics simulation for an active polymer chain was performed using a time step of d*t* = 0.001*τ*_BD_. The other simulation parameters are *kT* = 1.0, *σ* = 1.0, *d*_0_ = 0.5*σ*, 
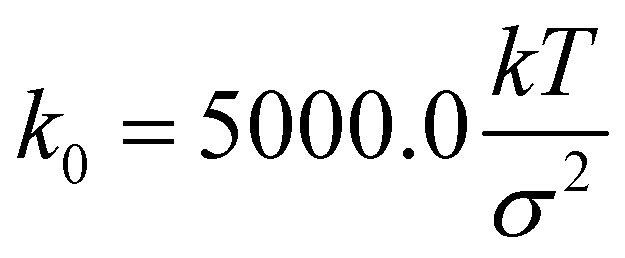
, *k*_ang_ = 45.0 *kT*, *γ* = 200 *kTτ*_BD_/*σ*^2^, *f*_m_ = 5.0 *kT*/*σ*, *θ*_0_ = *π*, and *E* = 10 000.0 *kT*/*σ*^3^.

### Training details of the Autoencoder

The Autoencoder architecture and training hyperparameters are presented in Table S3.† For the Trp-cage mini protein, we utilized the distance between all *C*_α_ atoms as input features, resulting in 190 (^20^*C*_2_ = 190) features for its 20 residues. Conversely, for active and passive polymer chains, we employed inter-bead distances, selecting 8 effective beads in an arithmetic progression with a step of 4, providing 28 (^8^*C*_2_ = 28) input features.^13^ Throughout the Autoencoder training process, we monitored two metrics: training loss and the fraction of variation explained (FVE) score. The FVE is defined by the equation12
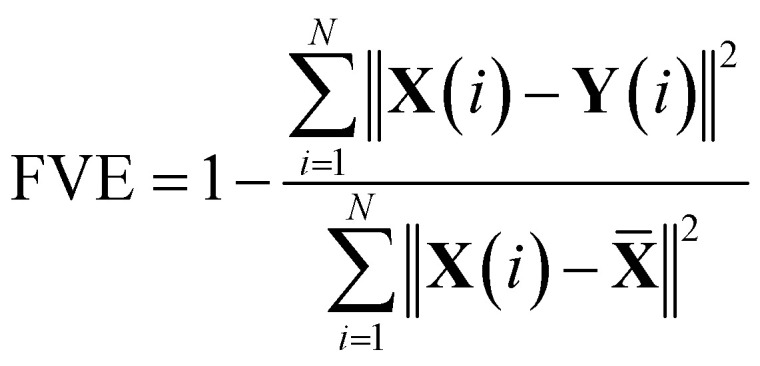
where **X**(*i*), **Y**(*i*), and **X̄** represent the input, output, and mean input, respectively, and *N* corresponds to the total number of features. The FVE score indicates the proportion of input data variance explained by the Autoencoder's reconstruction. Fig. S49(a–f)† depict the plots of these two metrics for all systems as described within the figure text. For active and passive polymer chains, a 2D latent dimension was chosen, whereas for the Trp-cage mini protein, a 4D latent dimension was utilized. Across all these latent dimensions, the FVE scores exceed 0.80, indicating that the Autoencoder's reconstruction explains at least 80% of the original data variance. Furthermore, the gradual decrease followed by saturation of the training loss curve suggests no overfitting during the Autoencoder's training process. However, in the case of the IDP α-synuclein, even with a high latent dimension of *L*_d_ = 7, we have observed a relatively low FVE score (∼0.60) (Fig. S50(a)†). Additionally, we have plotted the FES using the first two components of the latent space (Fig. S50(b)†). This plot indicates a lack of distinct minima in the free energy surface. Consequently, clustering the data for a proper state decomposition within this latent space is not feasible. Therefore, for α-synuclein, we have opted to use the radius of gyration (*R*_g_) as the reaction coordinate to discretize the trajectory into a specific number of states.

We conducted simulations for 3-state, 4-state, and active worm-like polymer chains using our in-house scripts written in C++. To simulate passive polymer chains, we utilized the open-source Software GROMACS-2022.^74,75^ Our Autoencoder model was trained using Python implementation of Tensorflow^76^ and Keras^77^ and the GPT model was built using PyTorch.^37^ The Markov State Model (MSM) analyses were performed using PyEMMA^78^ (v2.5.12), a Python library designed for efficient estimation and analysis of MSMs from molecular dynamics simulations.

## Data and code availability

The manuscript contains all the data. The code and detailed documentation for training the Autoencoder and GPT model are available on GitHub at the following URL: https://github.com/palash892/gpt_state_generation.

## Author contributions

Palash Bera: conceptualization, methodology, software, validation, formal analysis, investigation, data curation, writing – original draft, and writing – review & editing; Jagannath Mondal: conceptualization, methodology, validation, resources, writing – original draft, writing – review & editing, supervision, project administration, and funding acquisition.

## Conflicts of interest

There are no conflicts to declare.

## Supplementary Material

SC-OLF-D5SC00108K-s001
