# Supplementary material for: Accurate prediction of the kinetic sequence of physicochemical states using generative artificial intelligence
Source: Chem Sci. 2025 Apr 10;16(20):8735–51. doi: 10.1039/d5sc00108k (PMC12012632; doi:10.1039/d5sc00108k)
Supplement: SC-016-D5SC00108K-s001 [file SC-016-D5SC00108K-s001.pdf]

Supplementary Information (SI) for Chemical Science.
This journal is © The Royal Society of Chemistry 2025

Supporting Information for 'Accurate Prediction of the Kinetic Sequence of Physicochemical States Using Generative Artificial Intelligence '

Palash Bera[1,*] and Jagannath Mondal[1,†]

[1] *Tata Institute of Fundamental Research Hyderabad, Telangana 500046, India*

* palashb@tifrh.res.in

† jmondal@tifrh.res.in

SR1: Generation of the next state of a sequence using multinomial distribution:
Let's assume for an event, we have k classes (k possible outcomes) and n number of trials. For example, tossing a coin has two possible outcomes, and throwing a die has six possible outcomes. Now $x_1, x_2, ...x_k$ are the frequencies of each outcome, such that $\sum_{i=1}^{k} x_i = n$ and $p_1, p_2, ...p_k$ are the probabilities of each outcome occurring in a single trial, such that $\sum_{i=1}^{k} p_i = 1$. Then the probability distribution function is given by:

$$P(x_1, x_2, ...x_k) = \frac{n!}{x_1!x_2! \cdots x_k!} \cdot p_1^{x_1} p_2^{x_2} ... p_k^{x_k} \tag{1}$$

Binomial distribution is the special case of multinomial distribution where $x_1 + x_2 = n$, and $p_1 + p_2 = 1$, then the distribution will be

$$P(x_1) = \frac{n!}{x_1!(n - x_1)!} \cdot p_1^{x_1} p_2^{n-x_1} \tag{2}$$

As discussed in the main text for a given sequence, the GPT model will generate a probability distribution over the entire vocabulary/states. From this probability distribution, the next element of the sequence can be sampled using a multinomial distribution. Let's consider a four-state model, where the probability distribution of the next sequence is gen-prob = $[0.3286, 0.2406, 0.1770, 0.2539]$. These four values represent the probabilities of the four states, which are 0, 1, 2, and 3, respectively. The generation of the next state in the sequence is achieved by passing these probability values through a multinomial distribution. The multinomial distribution maintains these probability values and uses them to determine the next state in the sequence.

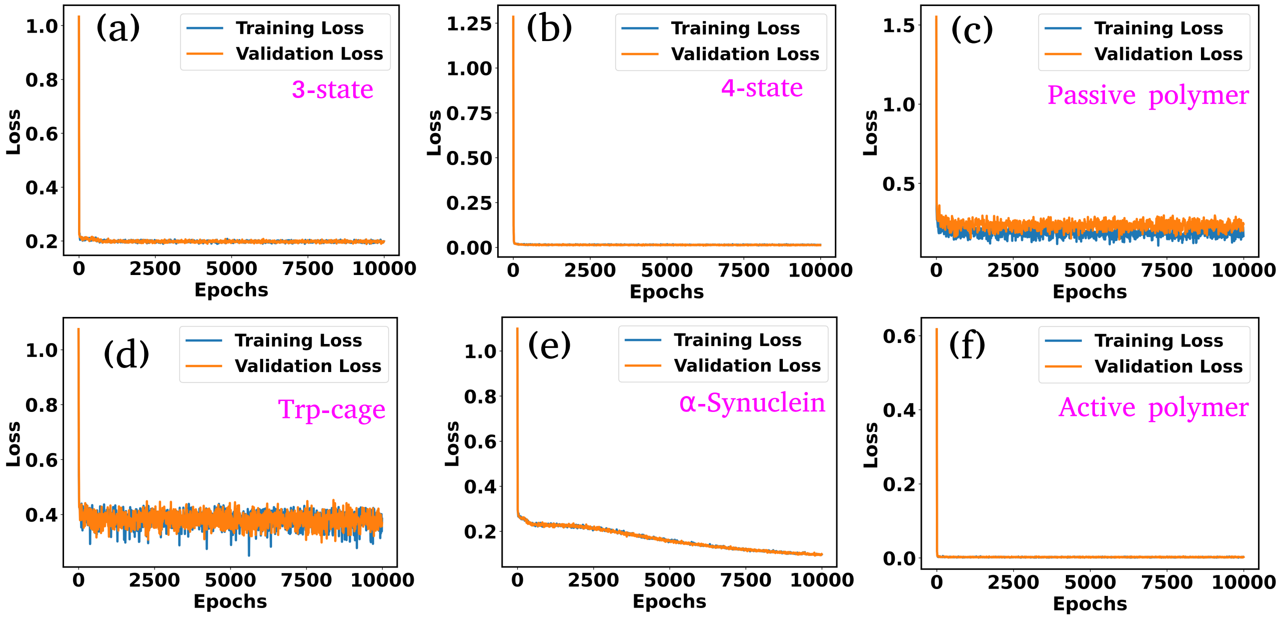

FIG. S1. Training and validation loss as a function of epochs for all of the system under investigation

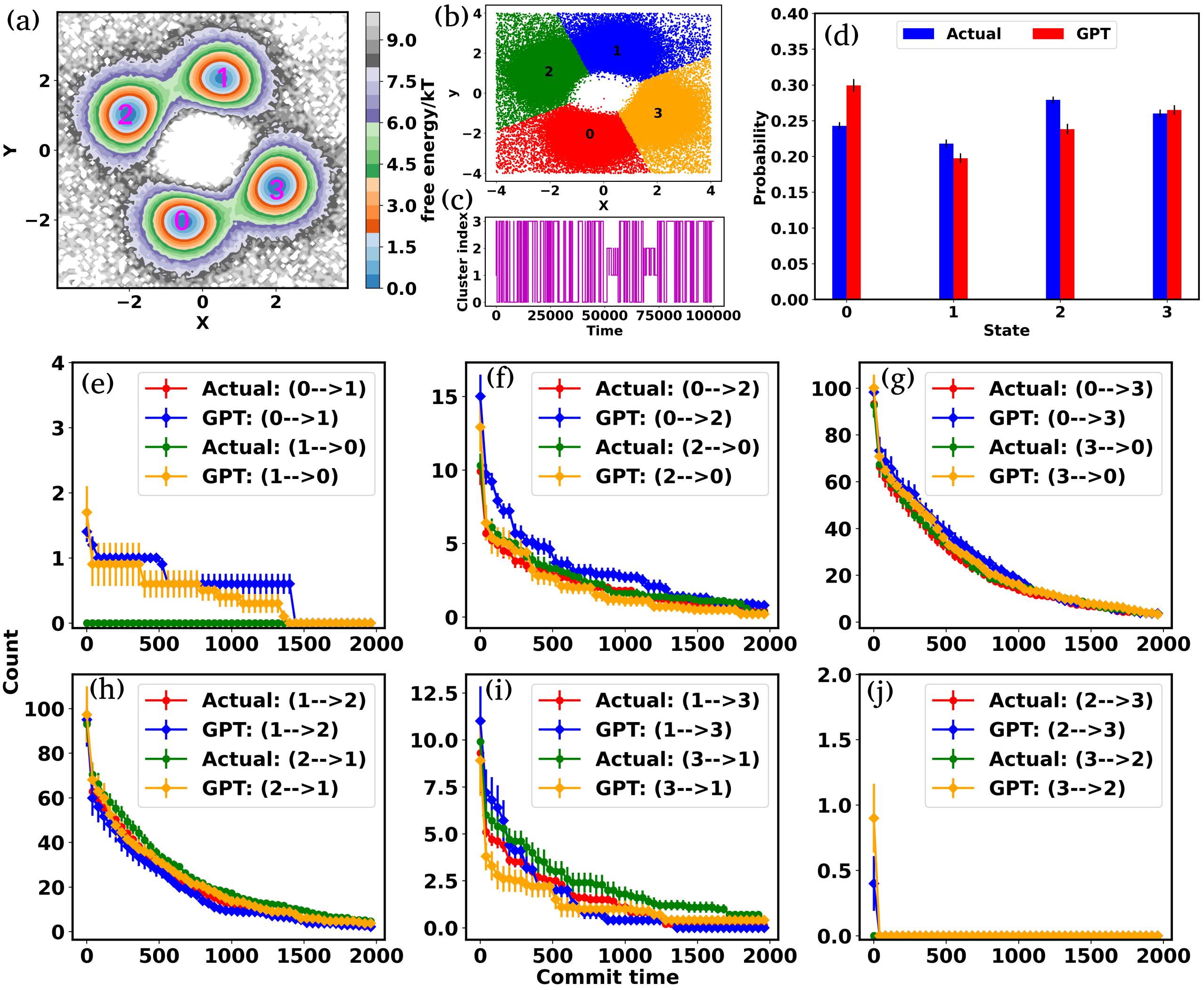

FIG. S2. (a) Free energy surface (FES) plot for 4-state toy model in their X and Y coordinate space. The particle can transition from one minimum to the other. (b) Scatter plots of the X and Y coordinate, with distinct clusters representing metastable states identified through K-means clustering. (c) The trajectory of the particle in 4-state potential after state decomposition. (d) The comparison of state probabilities between the actual and GPT-generated time series data for the 4-state toy model. (e-j) Transition counts as a function of commit time for a 4-state toy model. Here the error bar represents the standard error

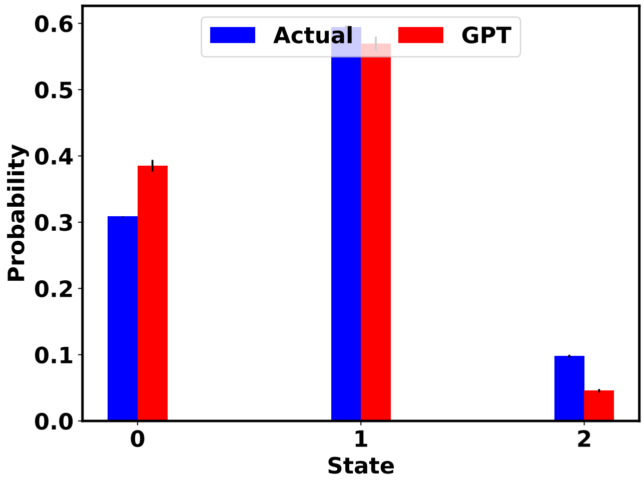

FIG. S3. The comparison of state probabilities between the actual and GPT-generated time series data for IDP α-Synuclein.

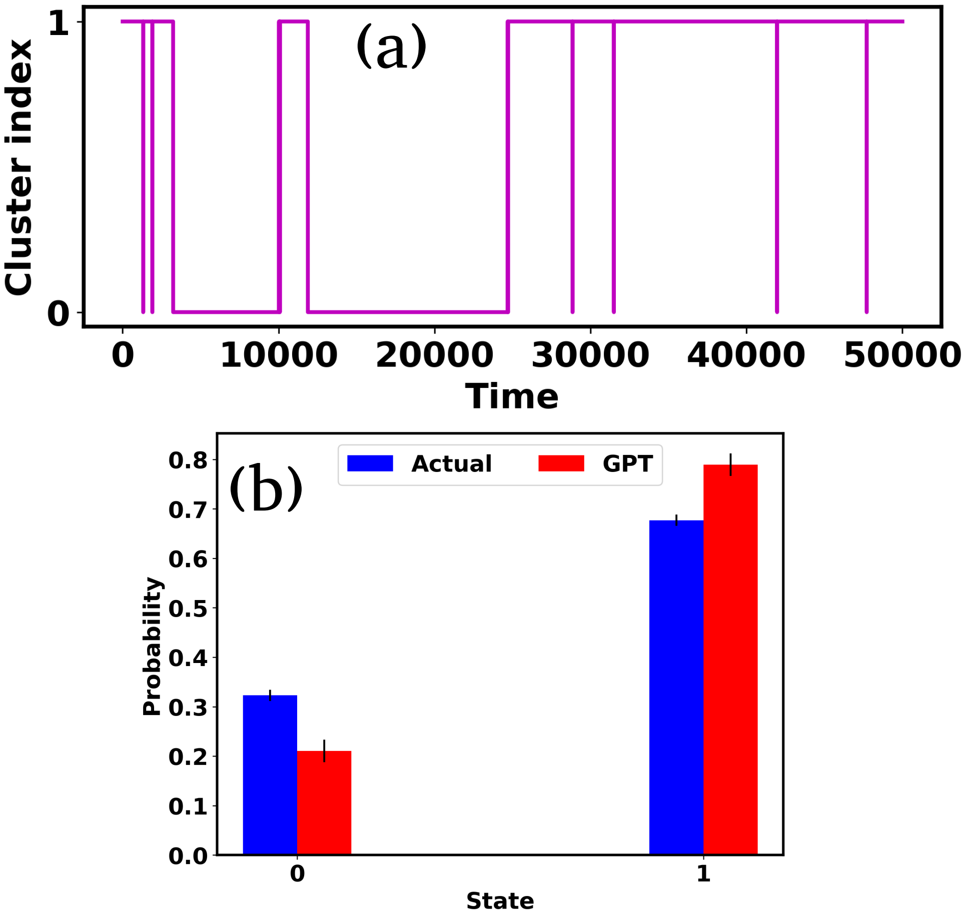

FIG. S4. (a) The trajectory of active worm like polymer chain after state decomposition. (b) The comparison of state probabilities between the actual and GPT-generated time series data for active worm like polymer chain.

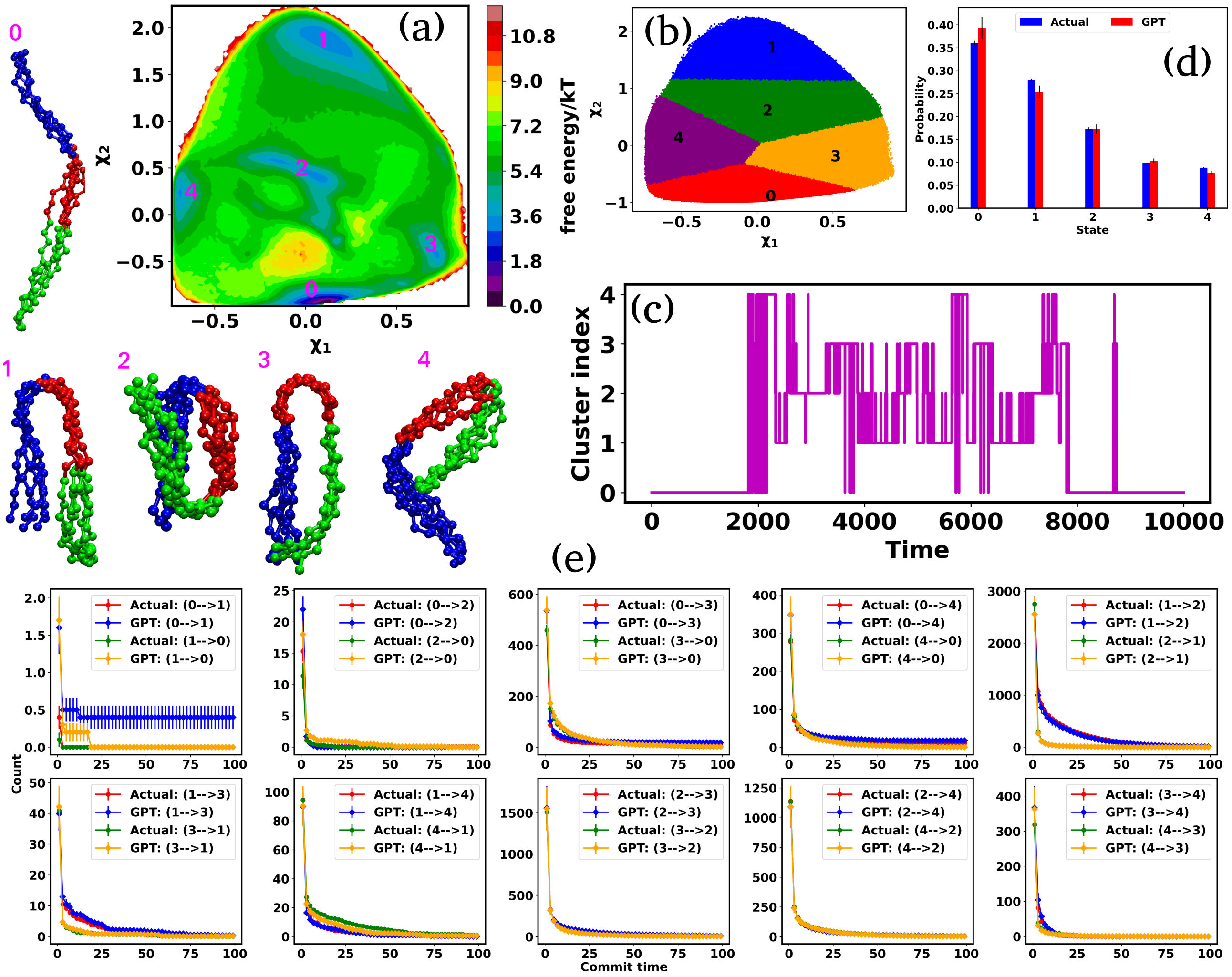

FIG. S5. (a) (a) 2D FES plot along latent space χ_1 and χ_2 for passive polymer chain, showing five distinct minima and extracted conformations. (b) The state decomposition of the MD trajectory is achieved through k-means clustering on the latent space. (c) The trajectory of the passive polymer chain after state decomposition. (d) The comparison of state probabilities between the actual and GPT-generated time series data for passive polymer chain. (e) Comparison of transition counts between actual and GPT-generated states for the passive polymer chain, showcasing the GPT model's ability to accurately capture state transitions. Here error bar represents the standard error.

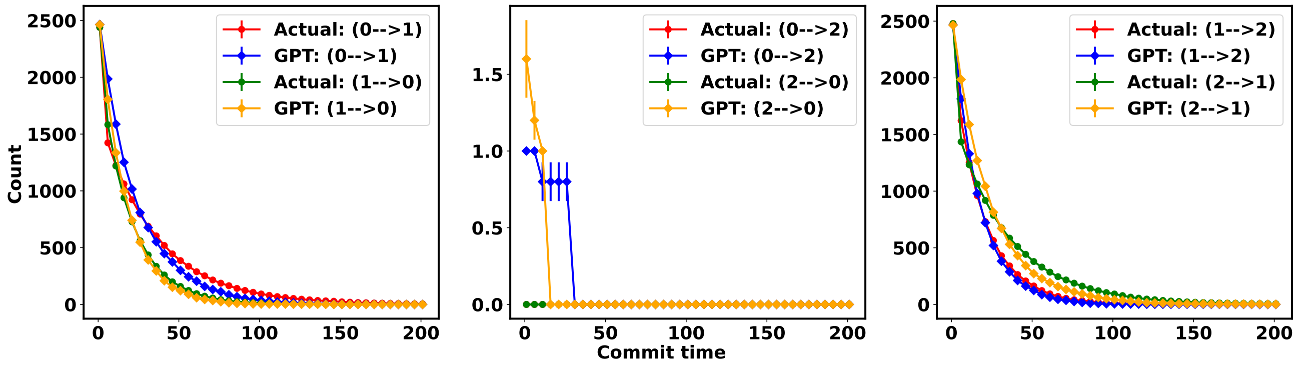

FIG. S6. The comparison of transition counts over commit time between the actual and GPT-generated time series data for 3-state toy model potential in absence of attention mechanism.

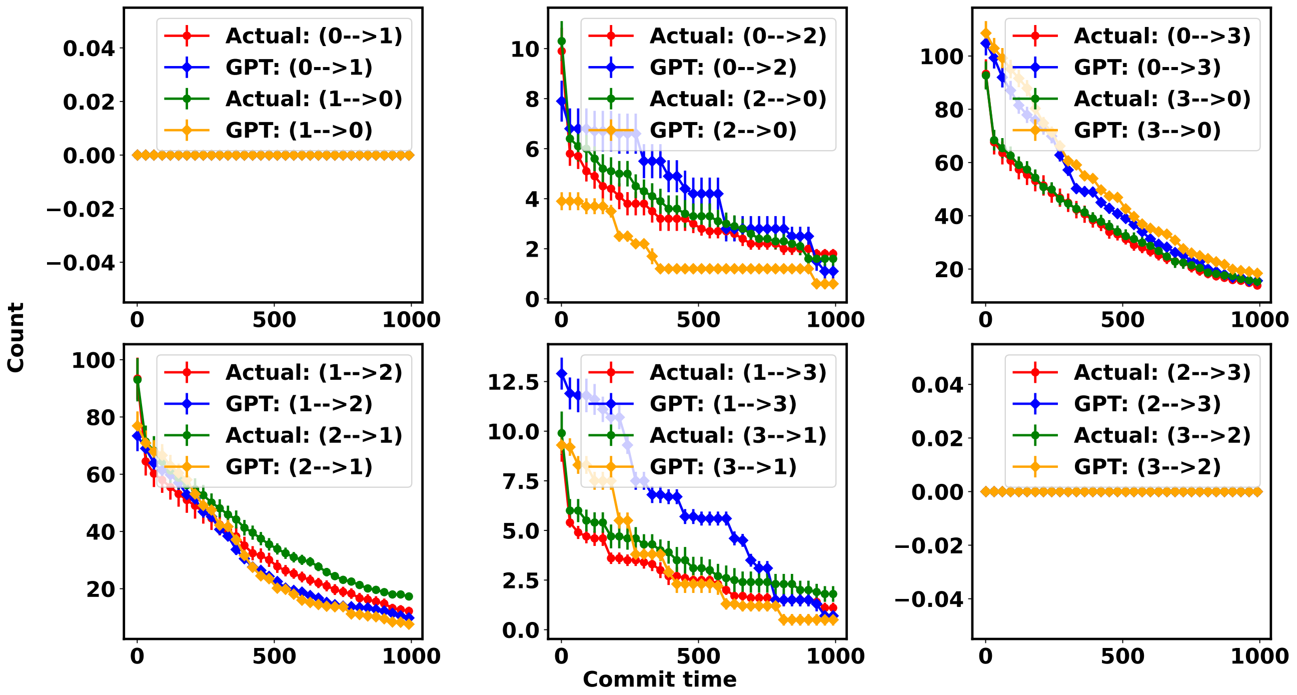

FIG. S7. The comparison of transition counts over commit time between the actual and GPT-generated time series data for 4-state toy model potential in absence of attention mechanism.

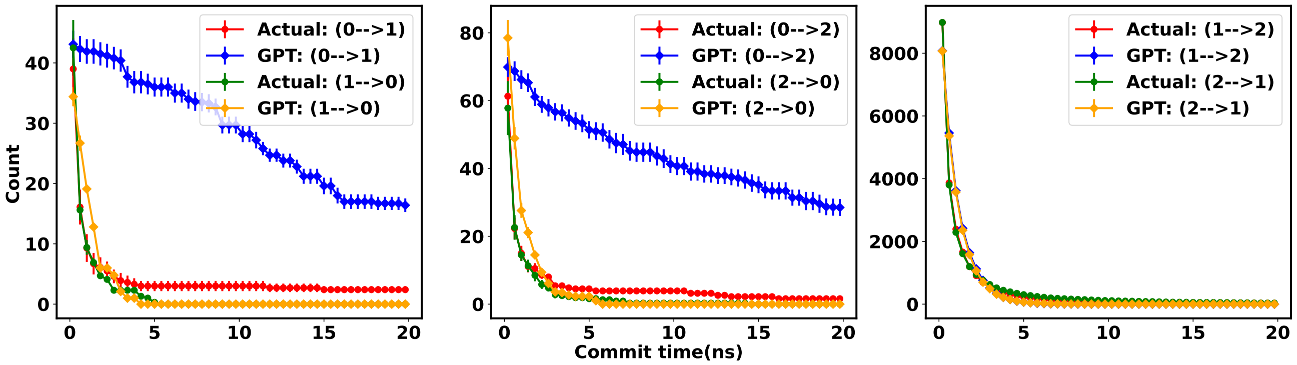

FIG. S8. The comparison of transition counts over commit time between the actual and GPT-generated time series data for Trp-cage mini protein in absence of attention mechanism.

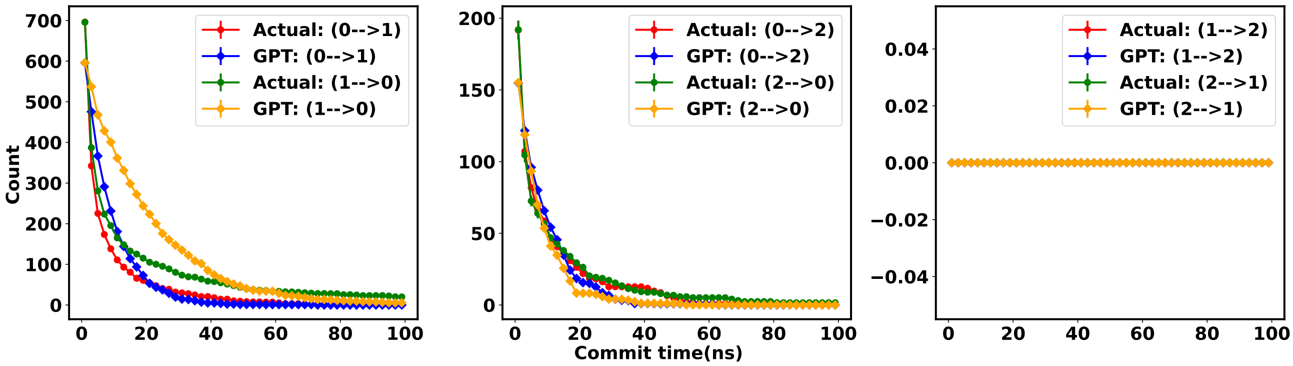

FIG. S9. The comparison of transition counts over commit time between the actual and GPT-generated time series data for α-Synuclein in absence of attention mechanism.

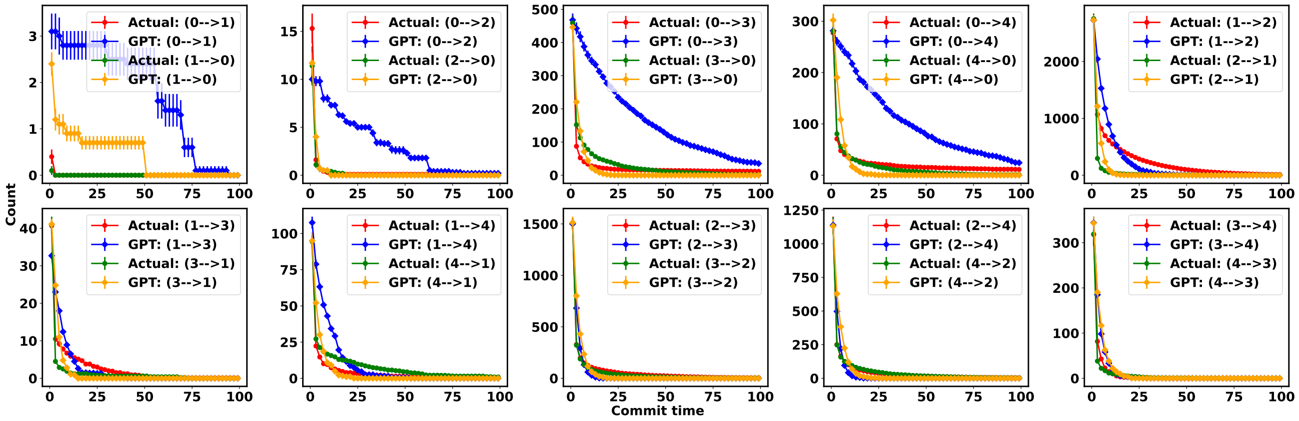

FIG. S10. The comparison of transition counts over commit time between the actual and GPT-generated time series data for passive polymer chain in absence of attention mechanism.

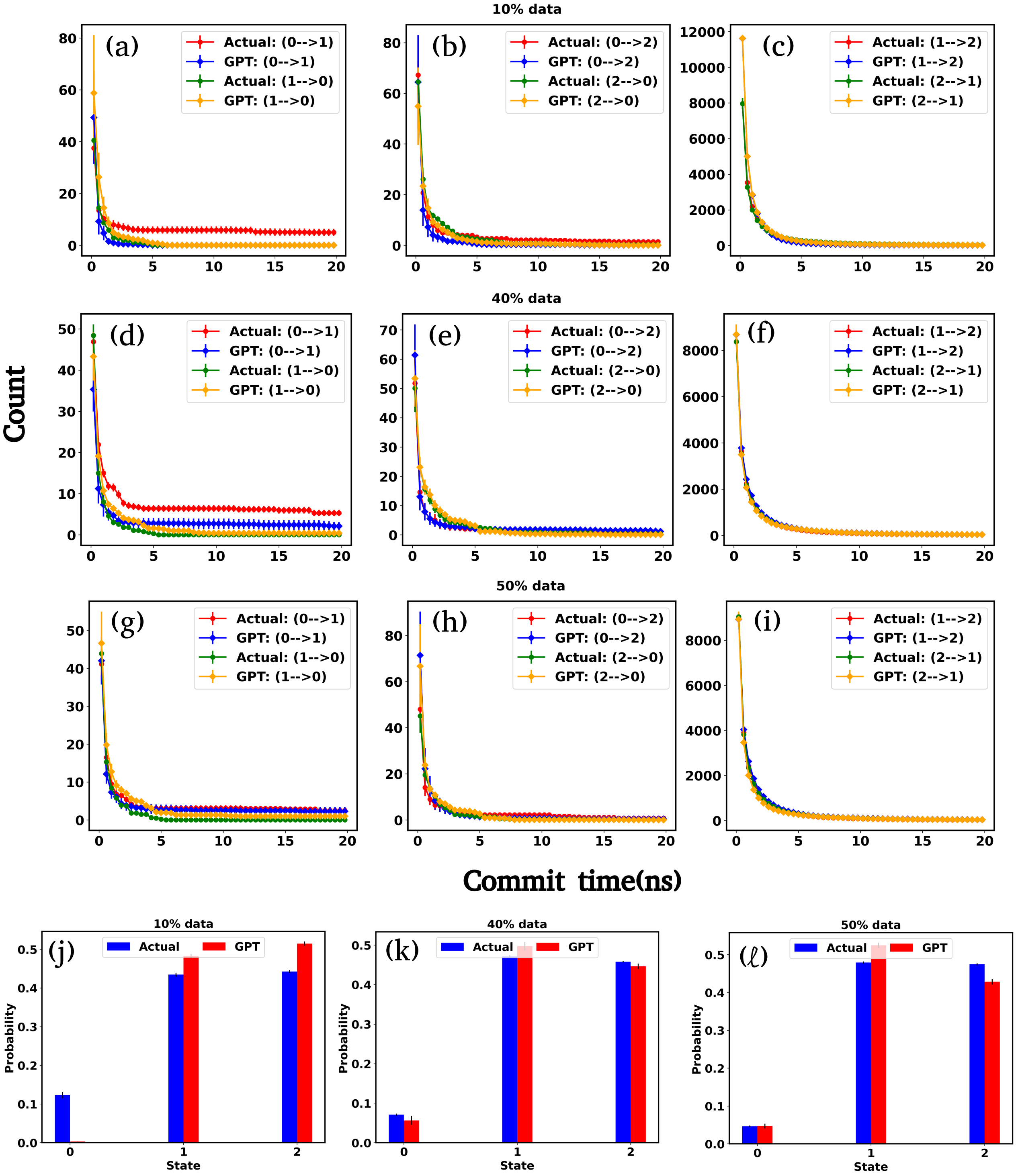

FIG. S11. (a-i) The comparison of transition counts between actual and GPT-generated states for various percentage of training data of Trp-cage. (j-l) The comparison of state probabilities between the actual and GPT-generated time series data for various percentage of training data of Trp-cage.

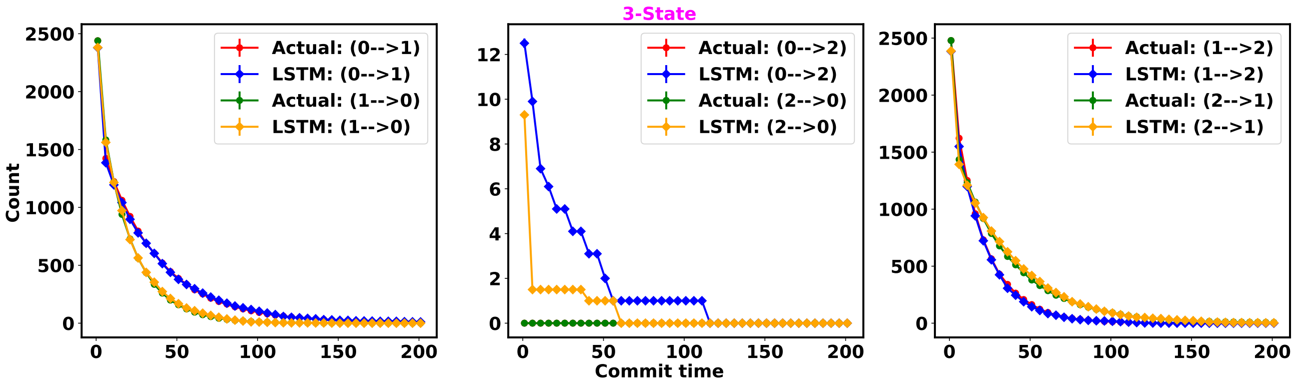

FIG. S12. Transition counts as a function of commit time for a 3-state toy model generated using LSTM.

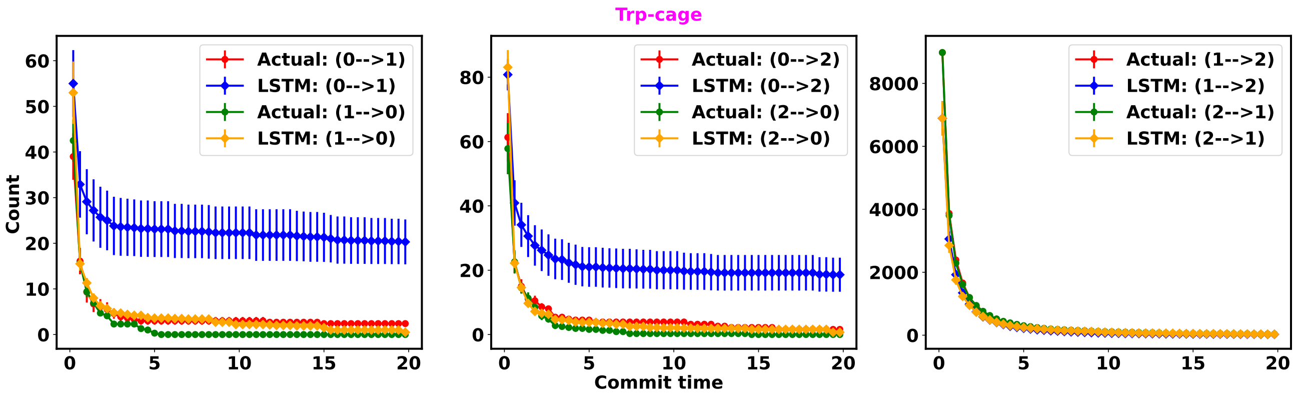

FIG. S13. Transition counts as a function of commit time for Trp-cage mini protein generated using LSTM.

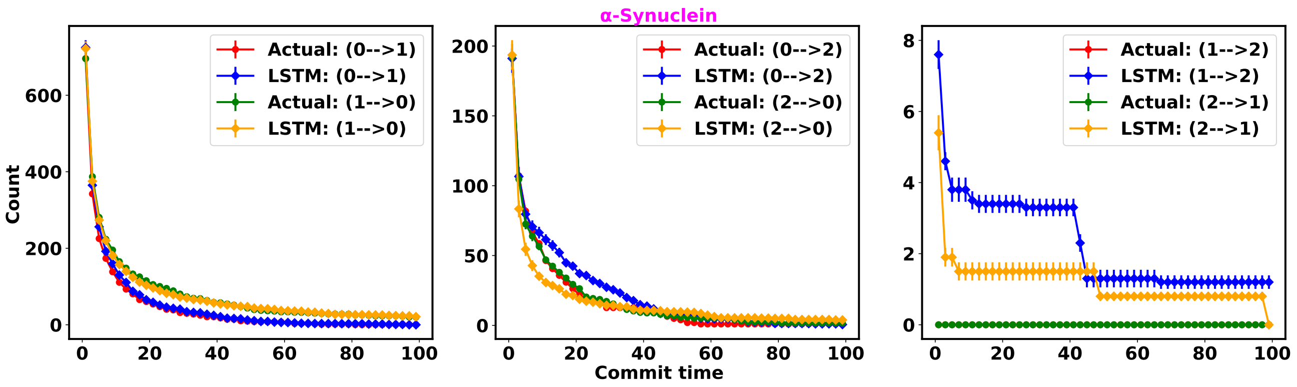

FIG. S14. Transition counts as a function of commit time for α-Synuclein generated using LSTM.

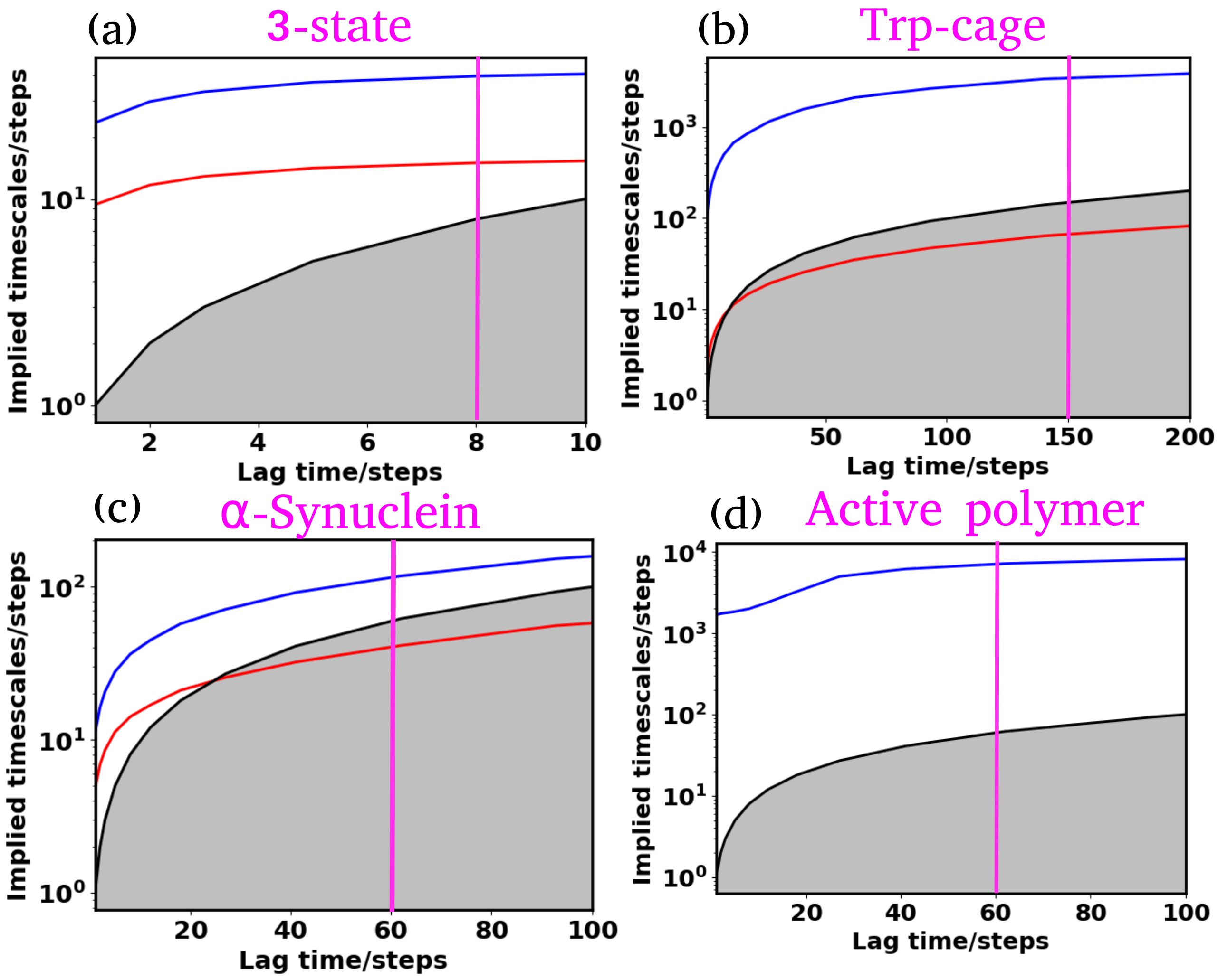

FIG. S15. Implied timescales (ITS) as a function of lag time for various system with the dotted line at the chosen lag time.

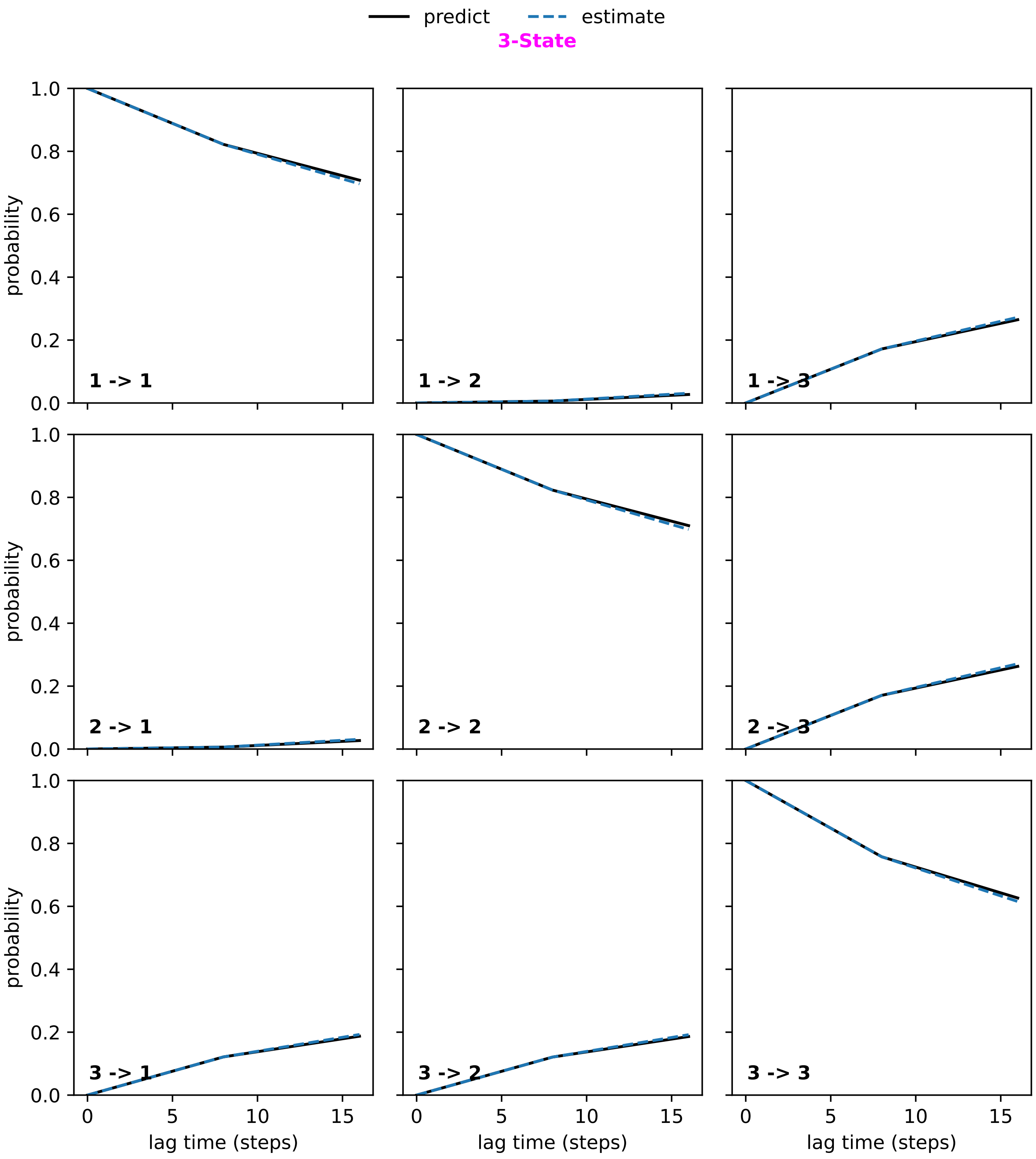

FIG. S16. The Chapman-Kolmogorov test performed for the 3-state toy model.

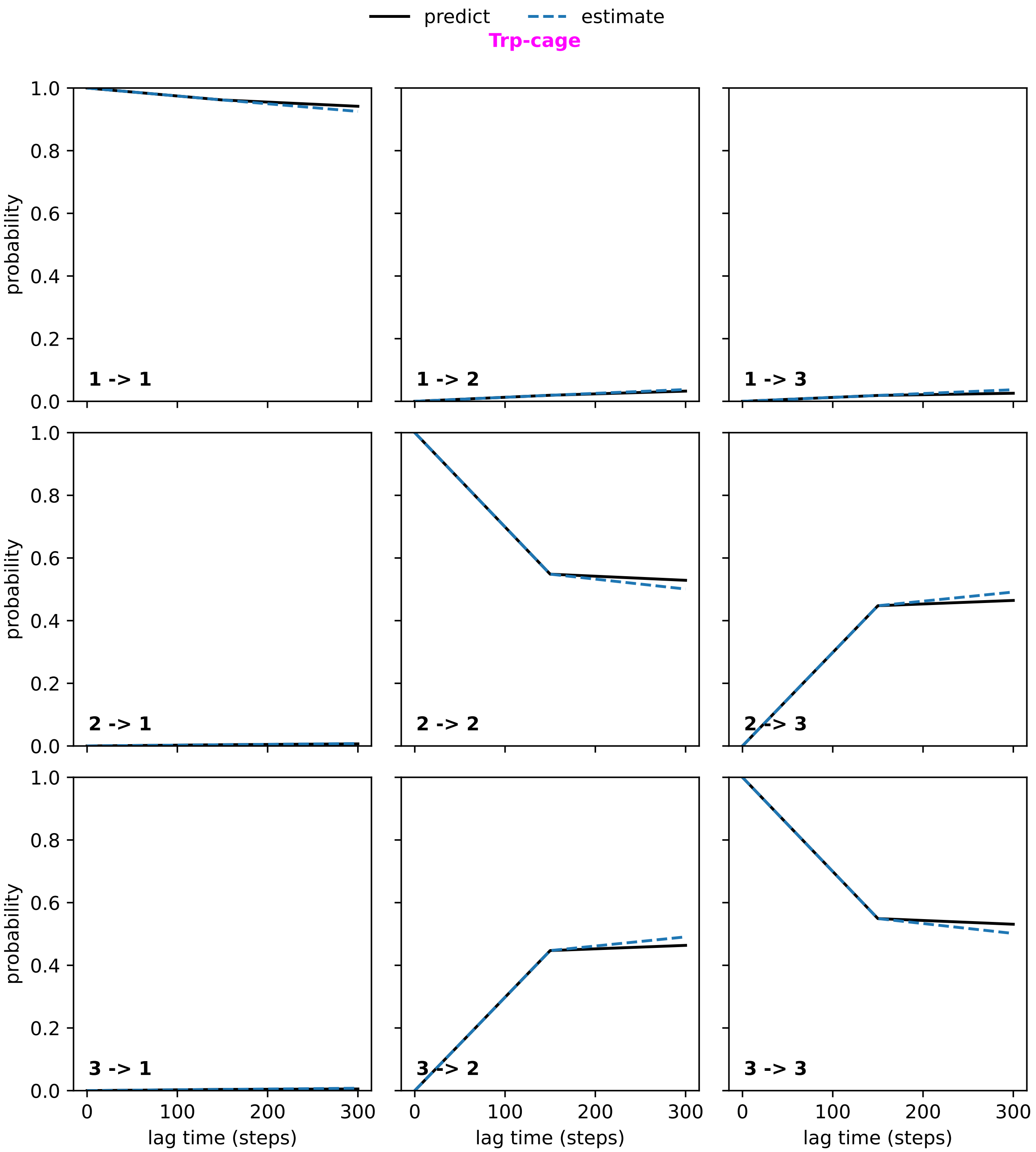

FIG. S17. The Chapman-Kolmogorov test performed for Trp-cage mini protein.

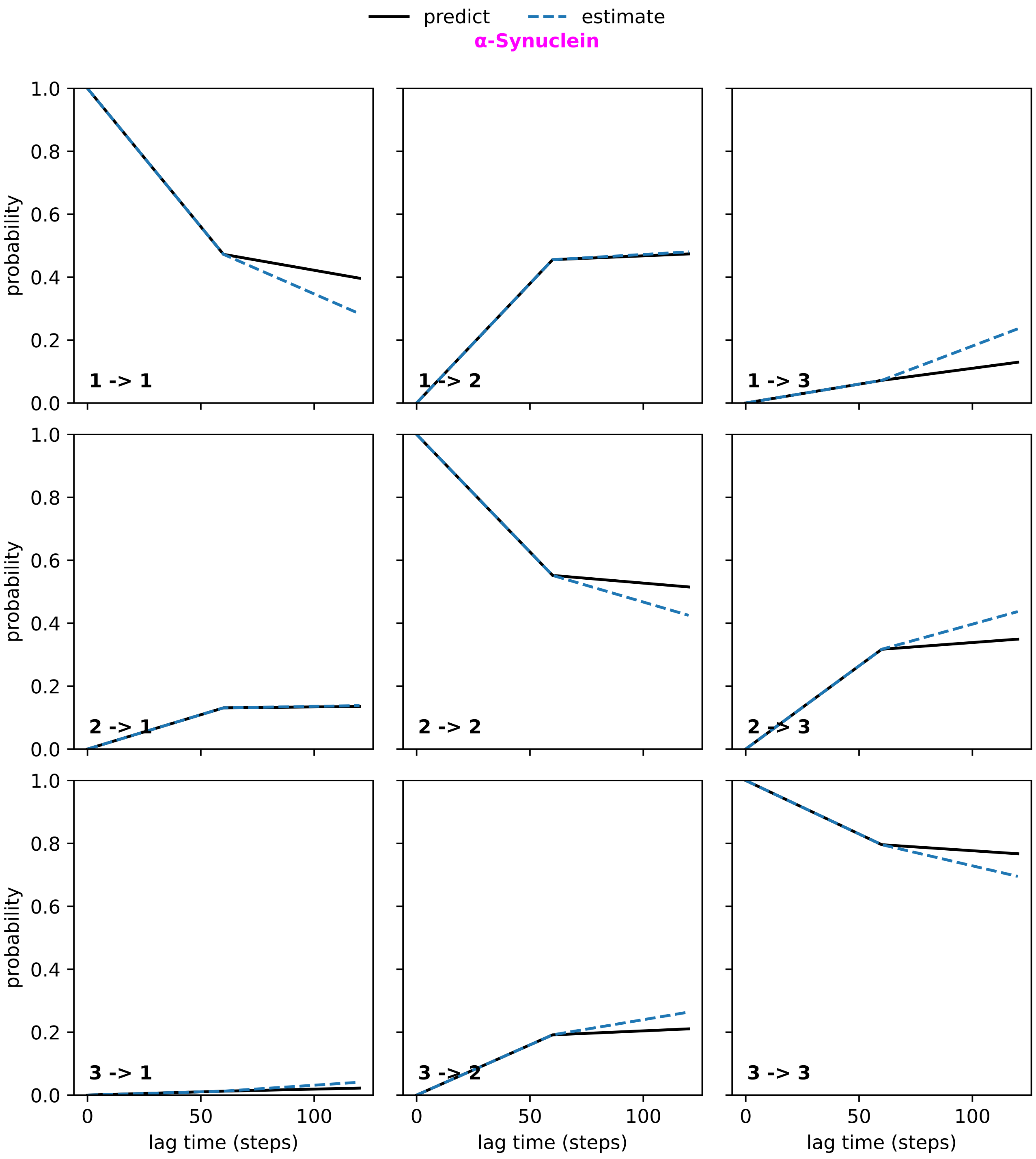

FIG. S18. The Chapman-Kolmogorov test performed for α-Synuclein.

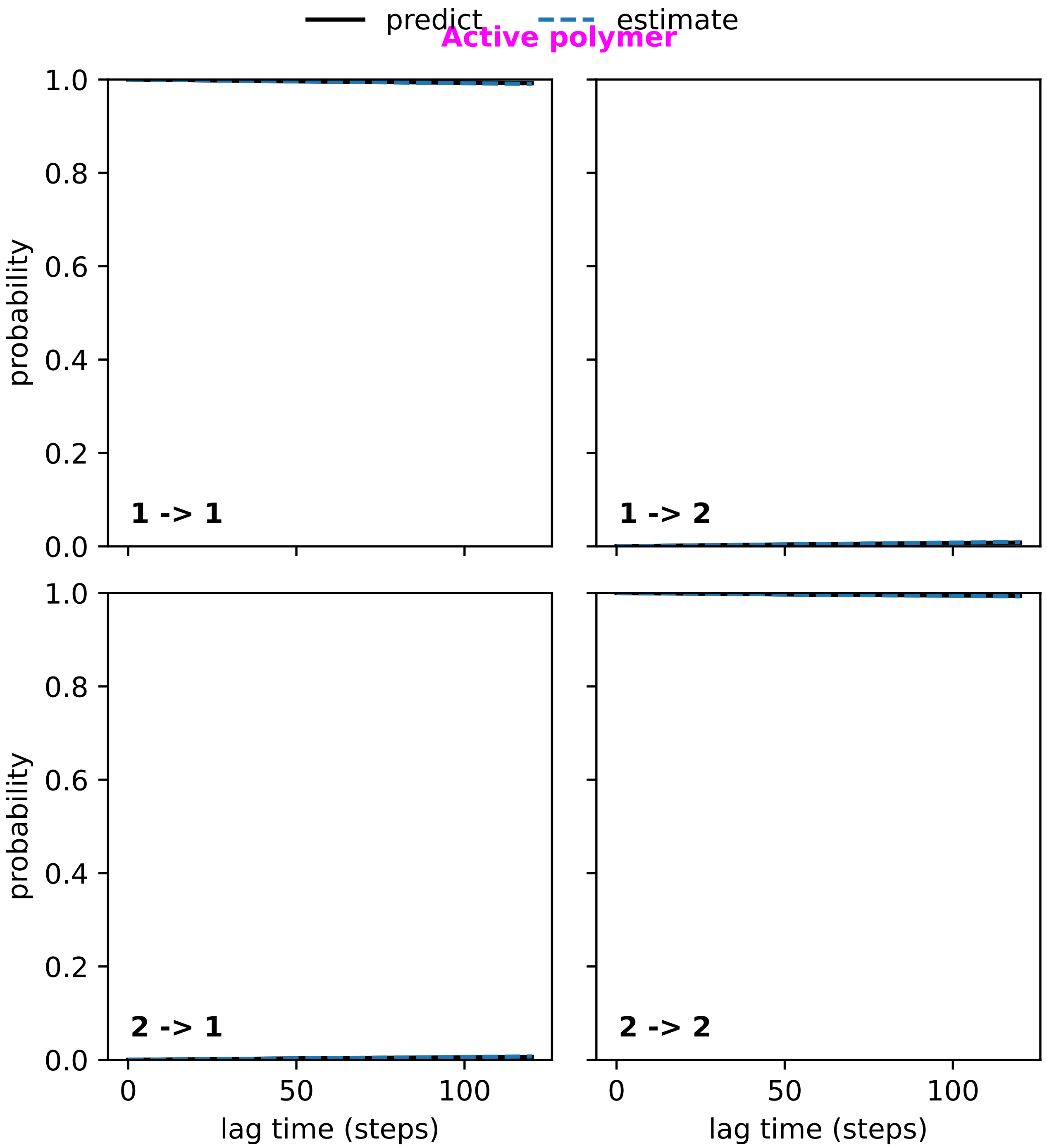

FIG. S19. The Chapman-Kolmogorov test performed for active polymer chain.

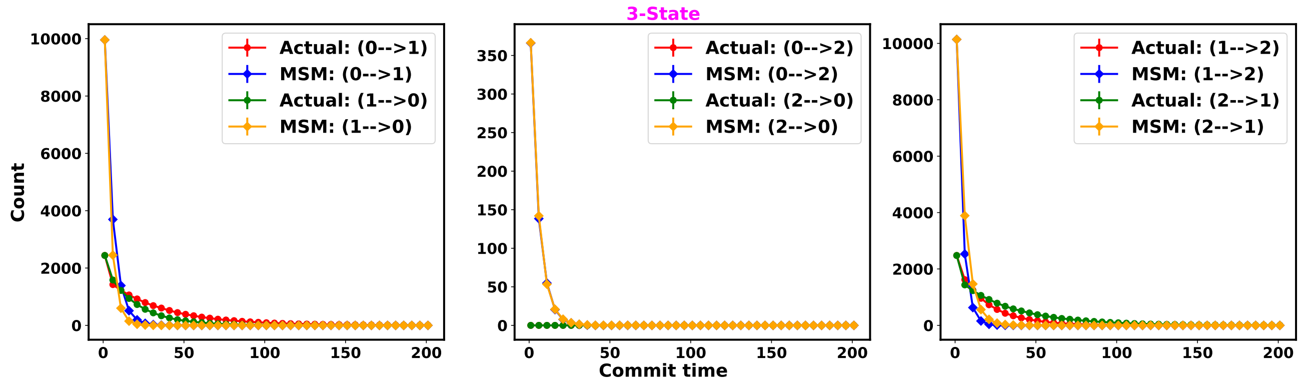

FIG. S20. Transition counts as a function of commit time for a 3-state toy model generated using MSM.

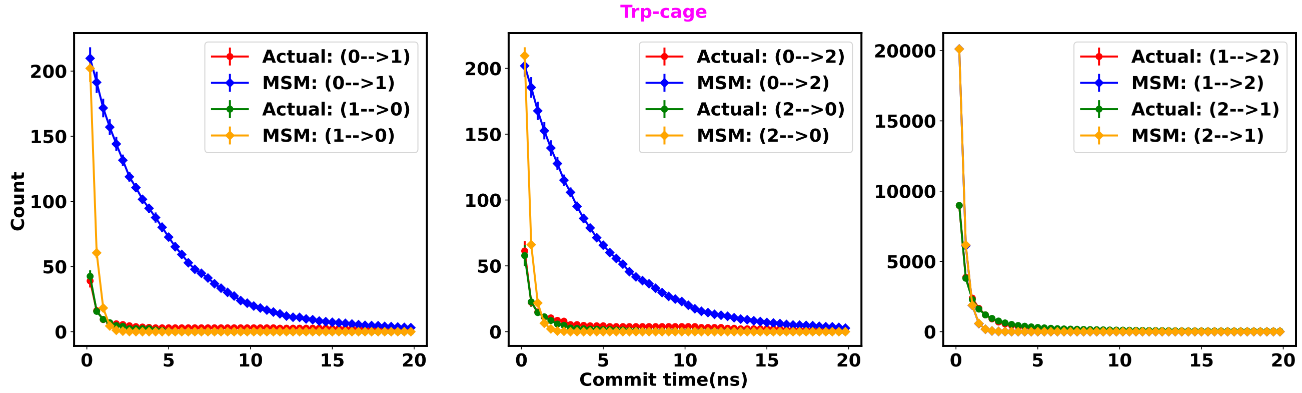

FIG. S21. Transition counts as a function of commit time for Trp-cage mini protein generated using MSM.

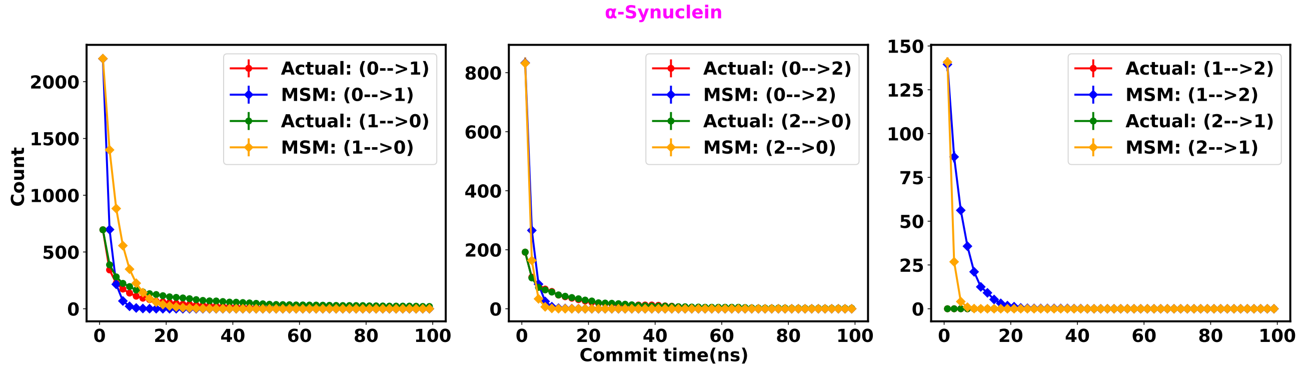

FIG. S22. Transition counts as a function of commit time α-Synuclein generated using MSM.

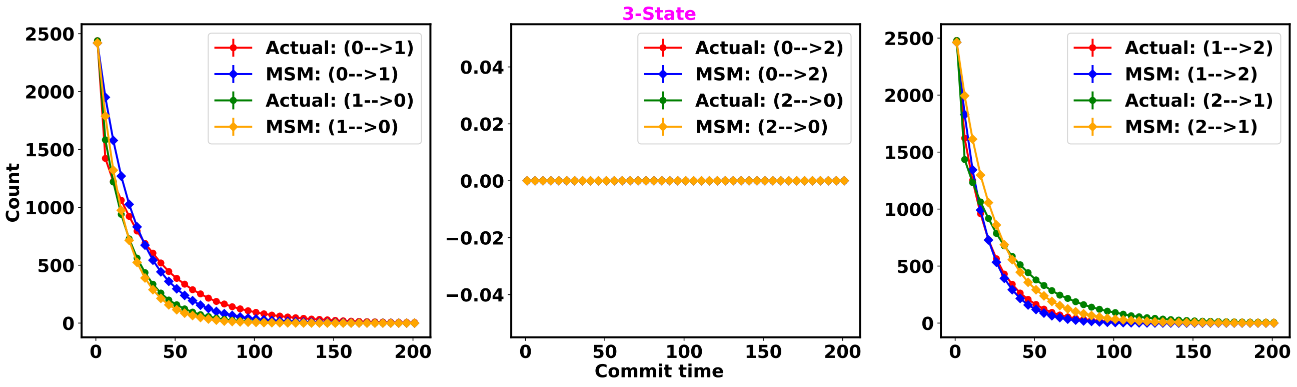

FIG. S23. Transition counts as a function of commit time for a 3-state toy model generated using MSM with lag time 1 steps.

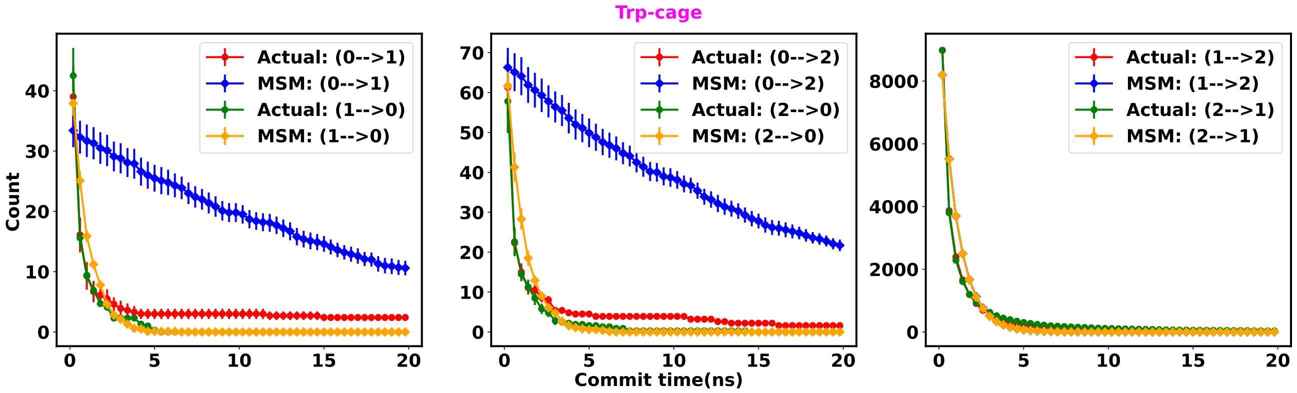

FIG. S24. Transition counts as a function of commit time Trp-cage mini protein generated using MSM with lag time 1 steps.

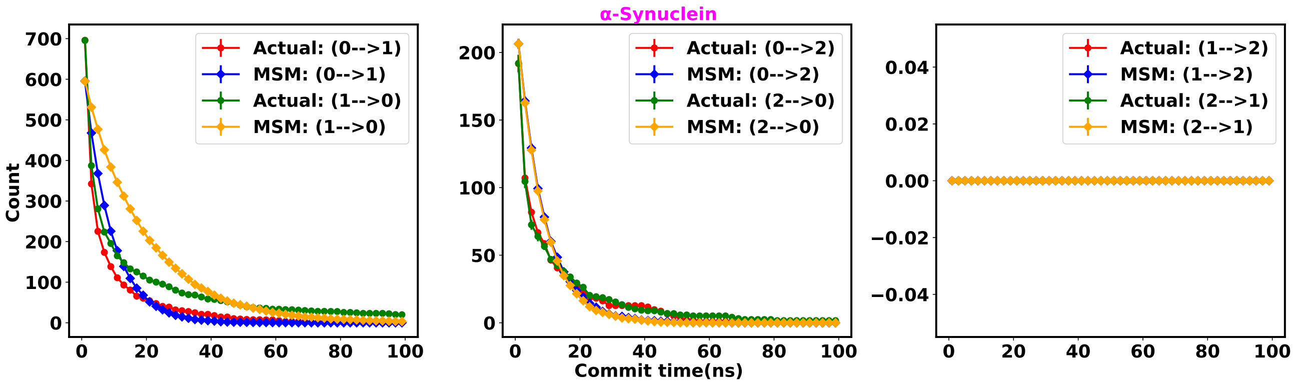

FIG. S25. Transition counts as a function of commit time α-Synuclein generated using MSM with lag time 1 steps.

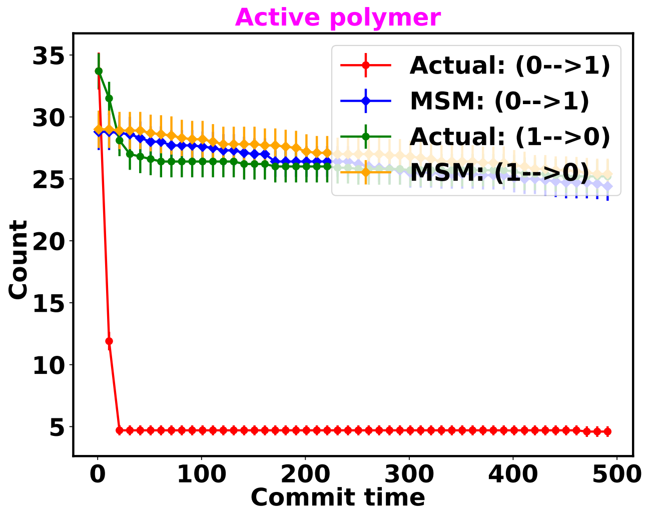

FIG. S26. Transition counts as a function of commit time active polymer chain generated using MSM with lag time 1 steps.

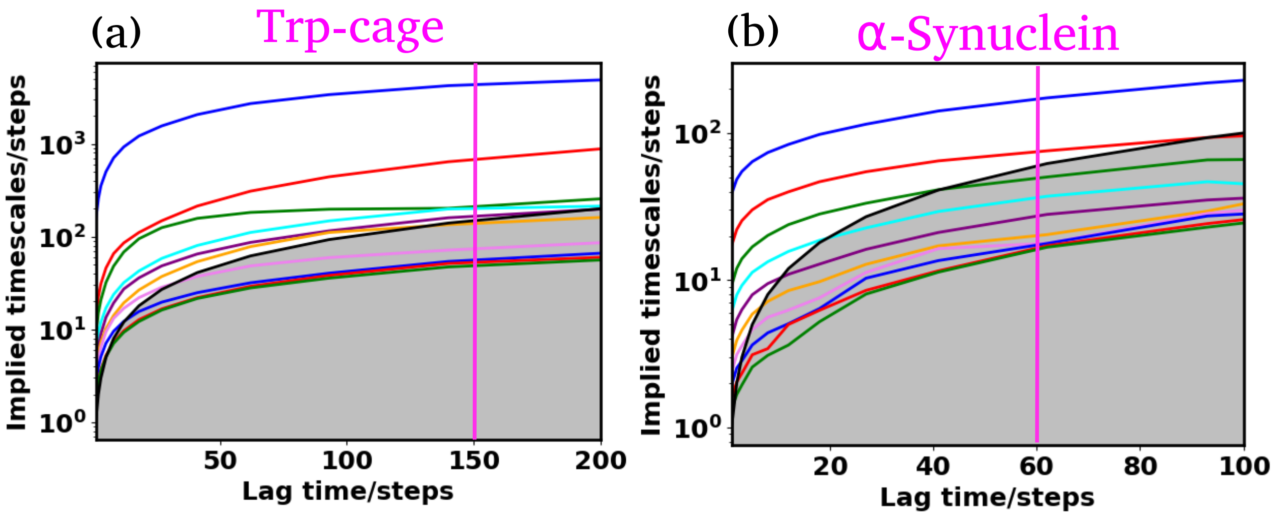

FIG. S27. Implied timescales (ITS) as a function of lag time for various system using many cluster with the dotted line at the chosen lag time.

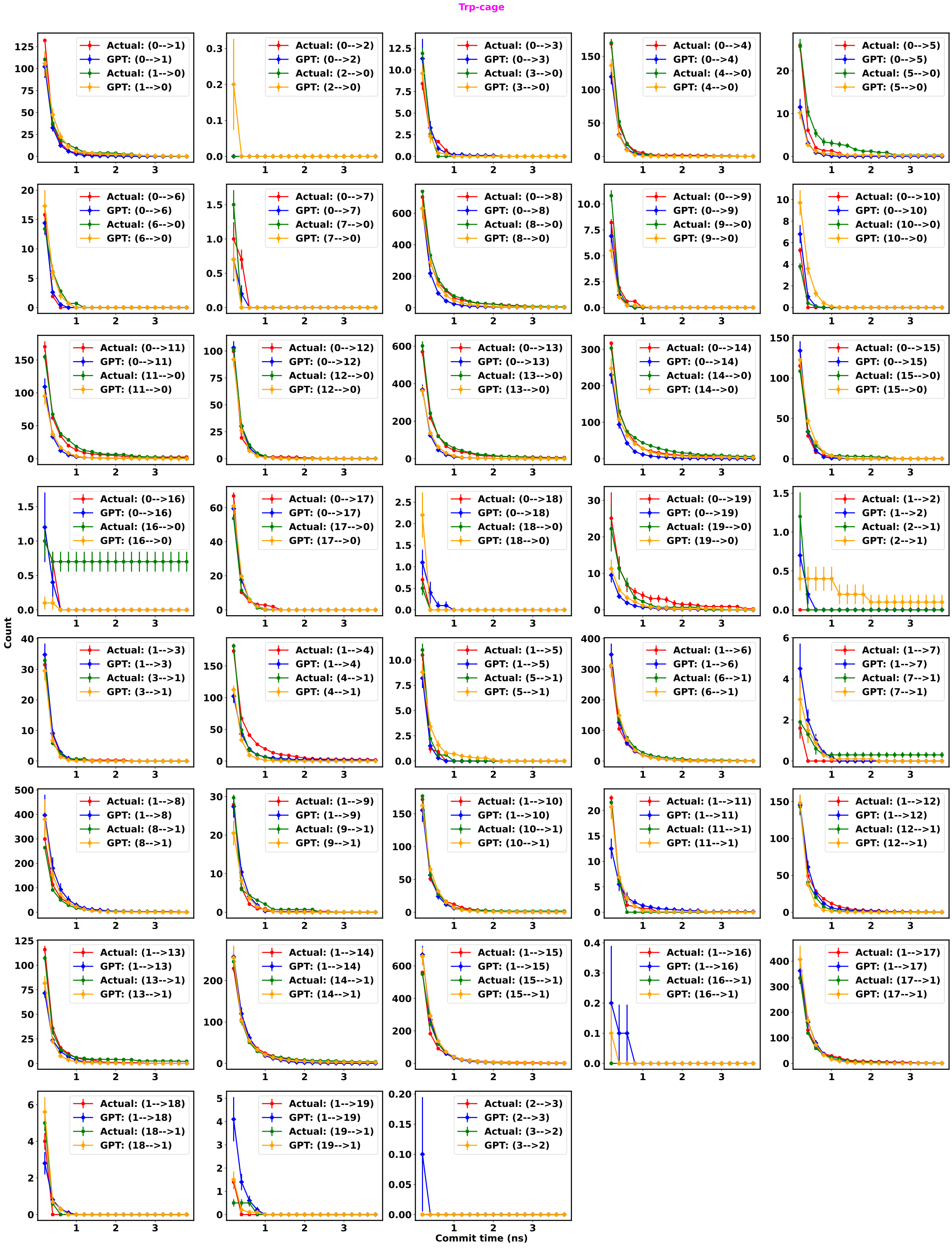

FIG. S28. State to state transition count as a function of commit time for Trp-cage mini protein with GPT model using a large number of cluster.

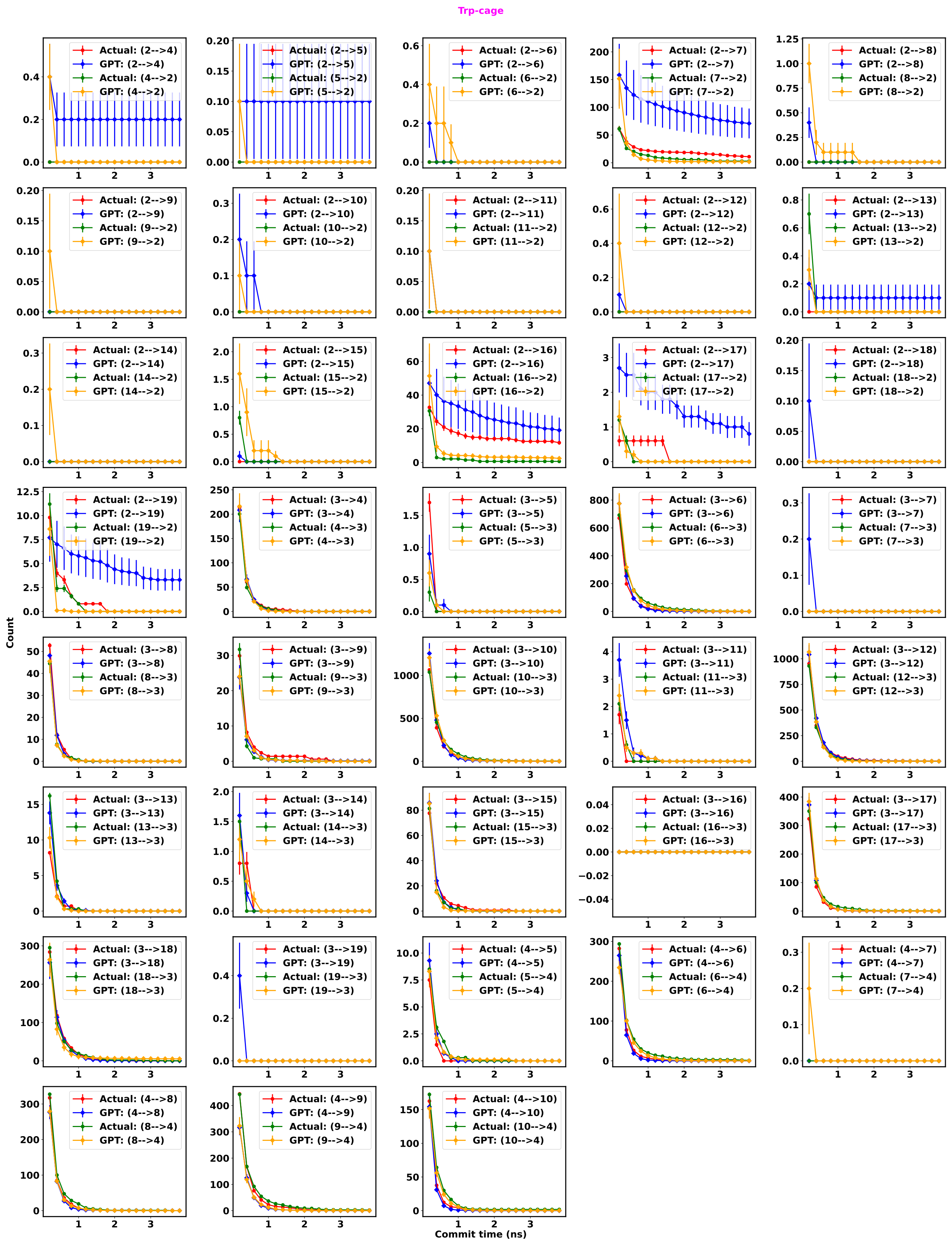

FIG. S29. State to state transition count as a function of commit time for Trp-cage mini protein with GPT model using a large number of cluster.

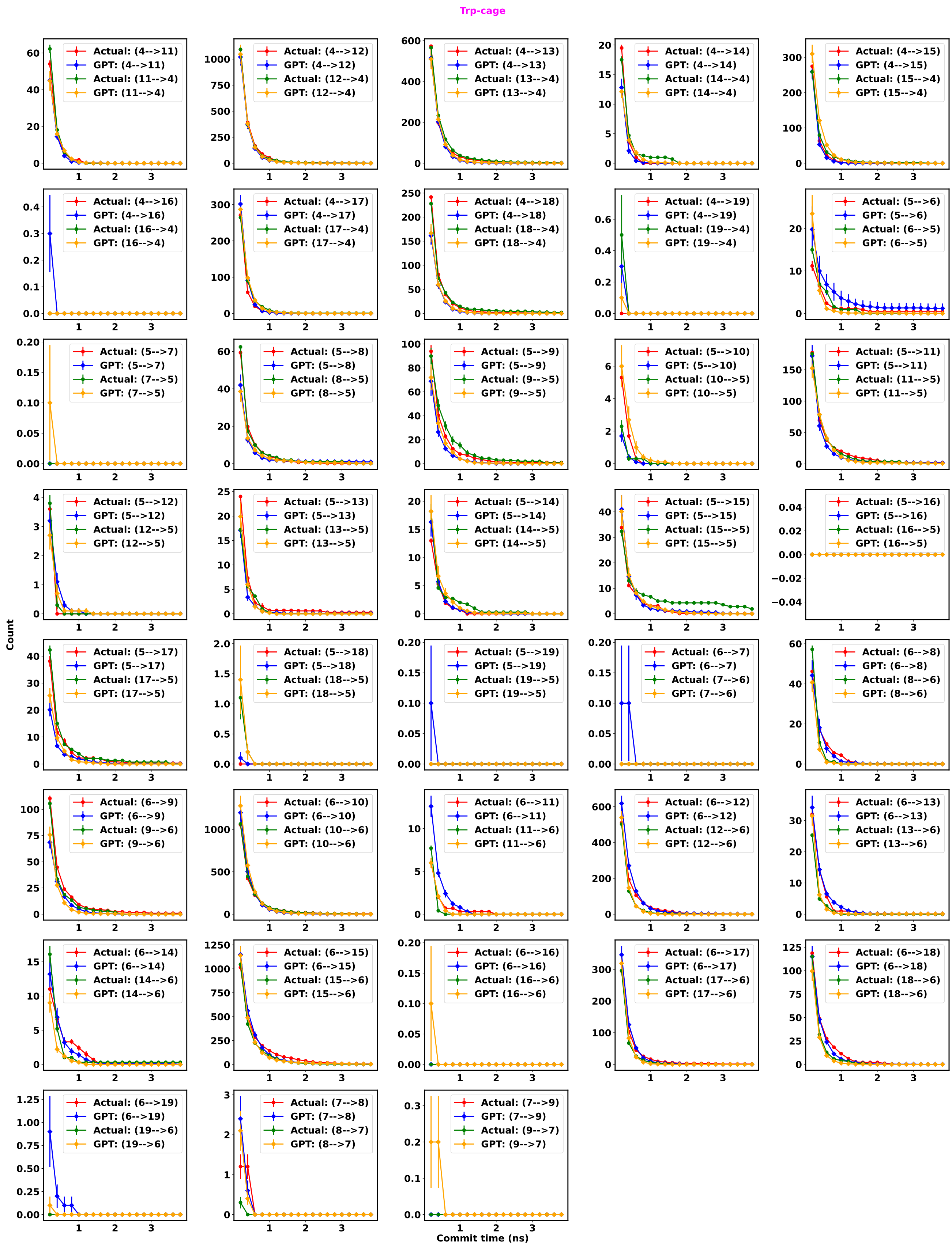

FIG. S30. State to state transition count as a function of commit time for Trp-cage mini protein with GPT model using a large number of cluster.

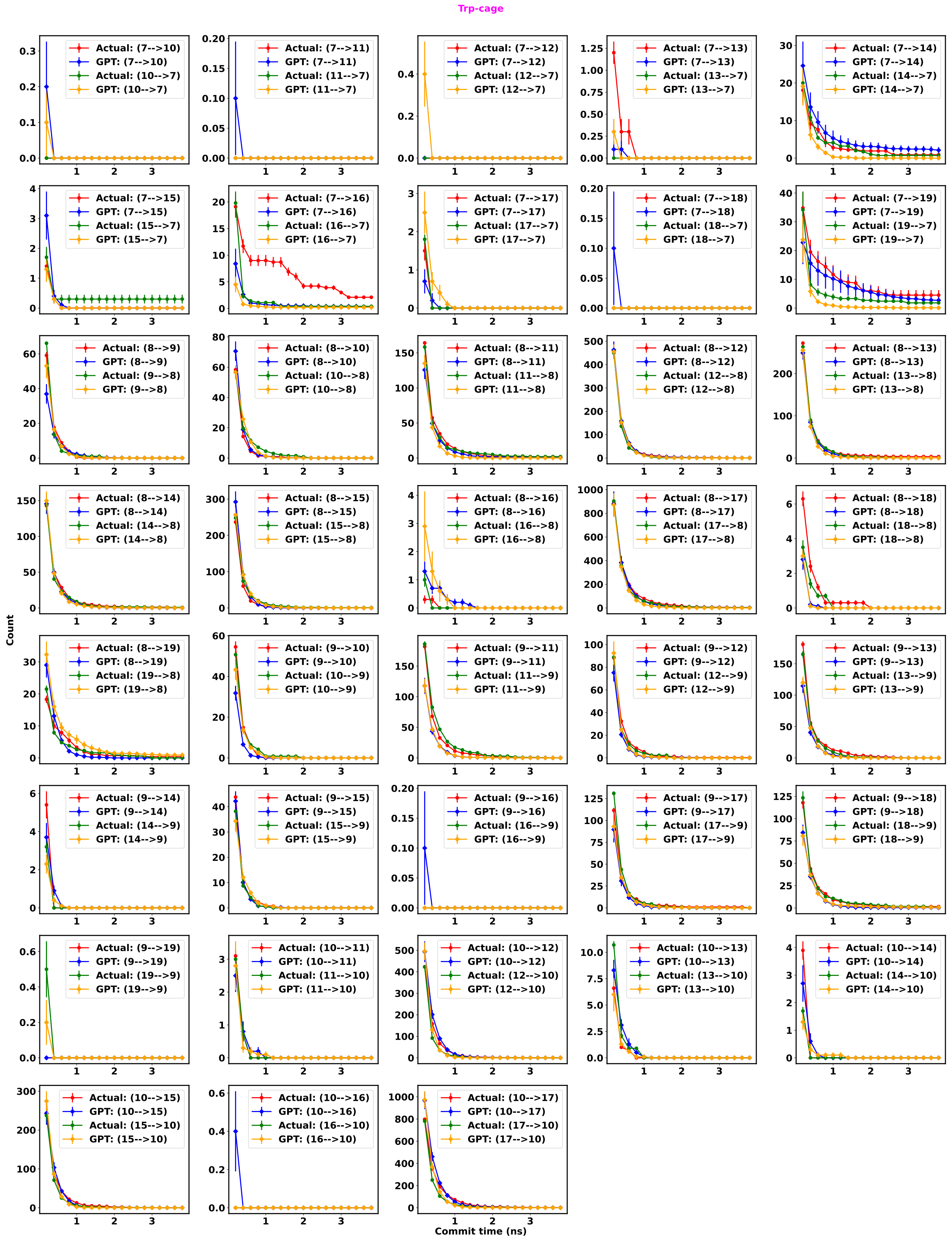

FIG. S31. State to state transition count as a function of commit time for Trp-cage mini protein with GPT model using a large number of cluster.

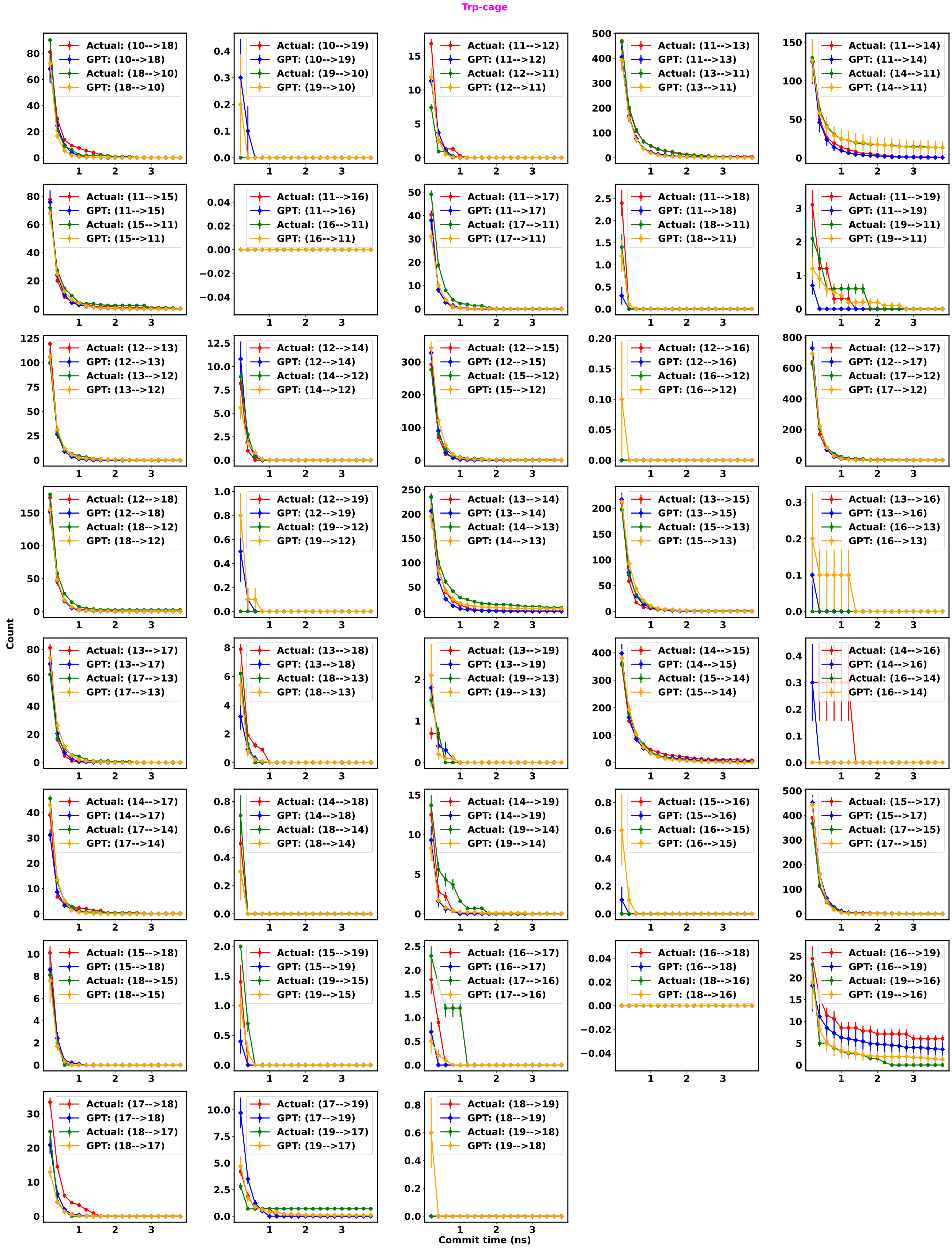

FIG. S32. State to state transition count as a function of commit time for Trp-cage mini protein with GPT model using a large number of cluster.

Trp-cage

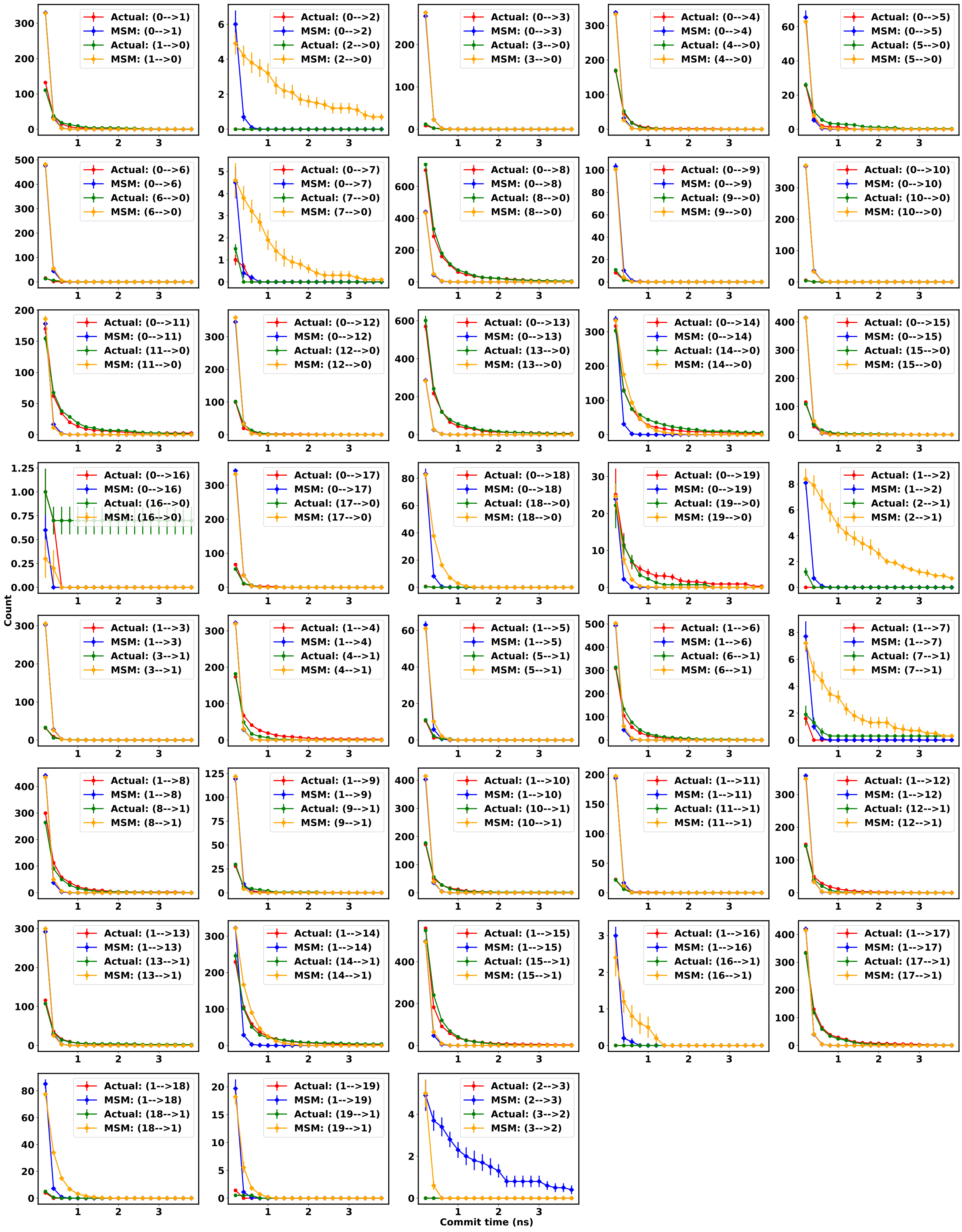

FIG. S33. State to state transition count as a function of commit time for Trp-cage mini protein with MSM using a large number of cluster.

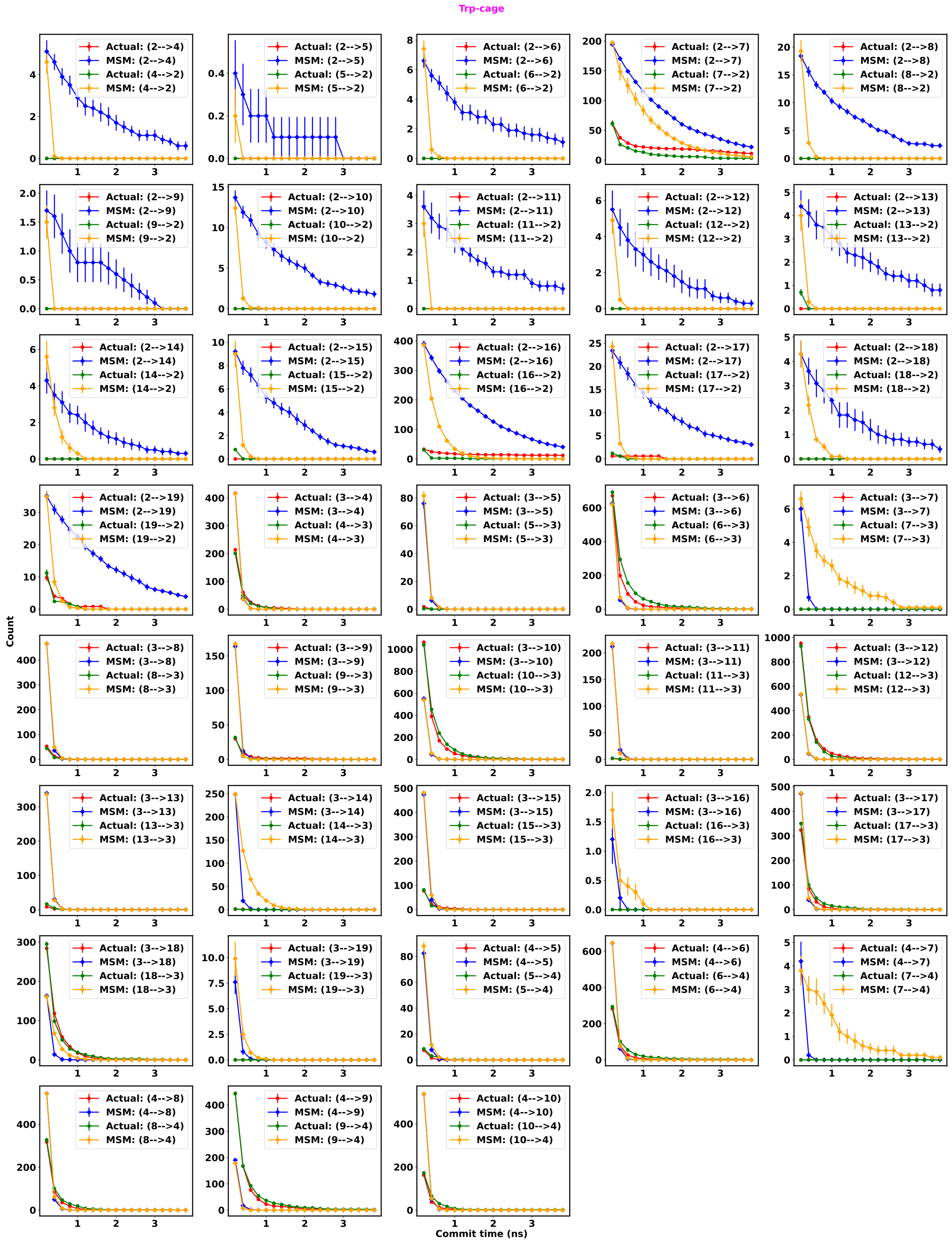

FIG. S34. State to state transition count as a function of commit time for Trp-cage mini protein with MSM using a large number of cluster.

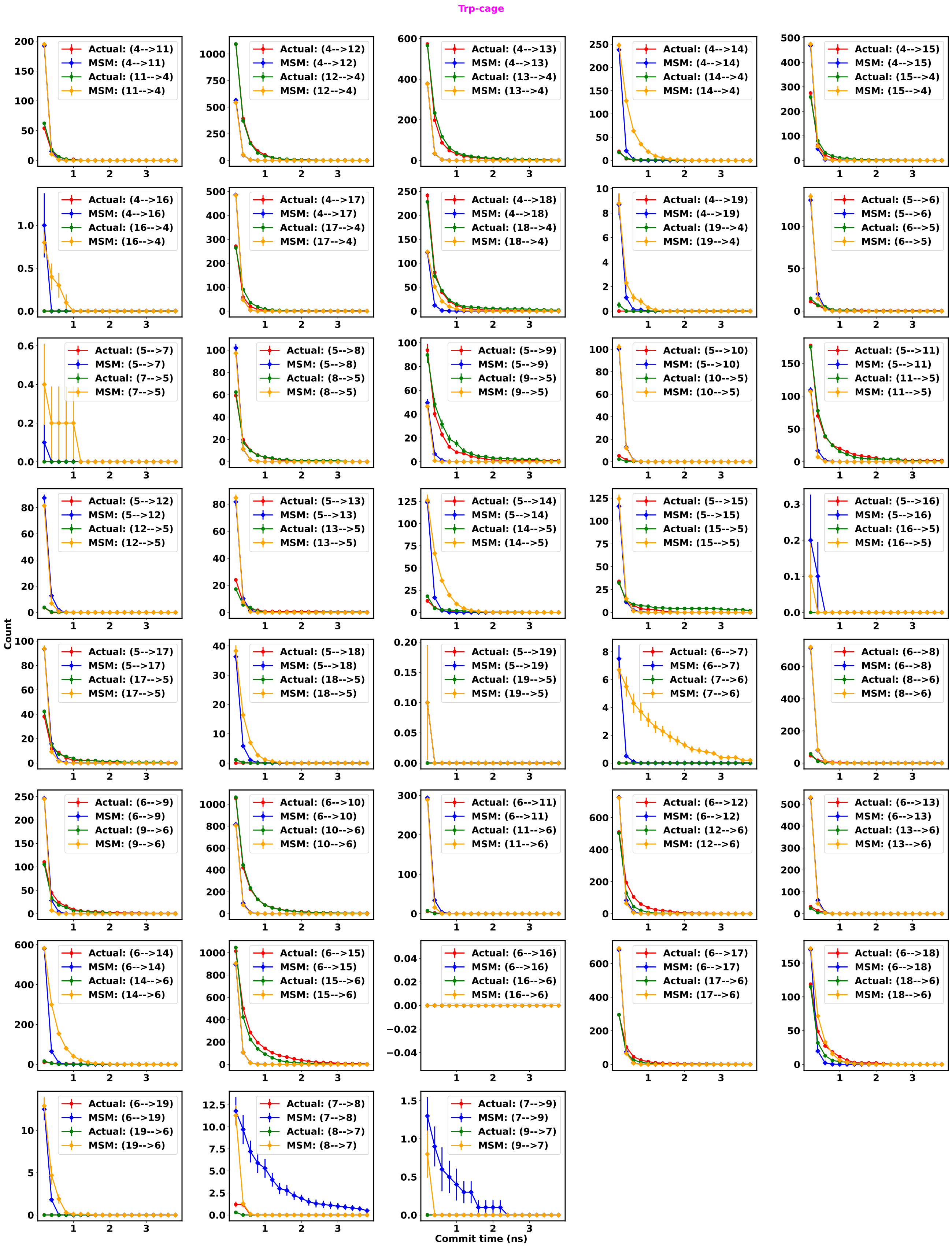

FIG. S35. State to state transition count as a function of commit time for Trp-cage mini protein with MSM using a large number of cluster.

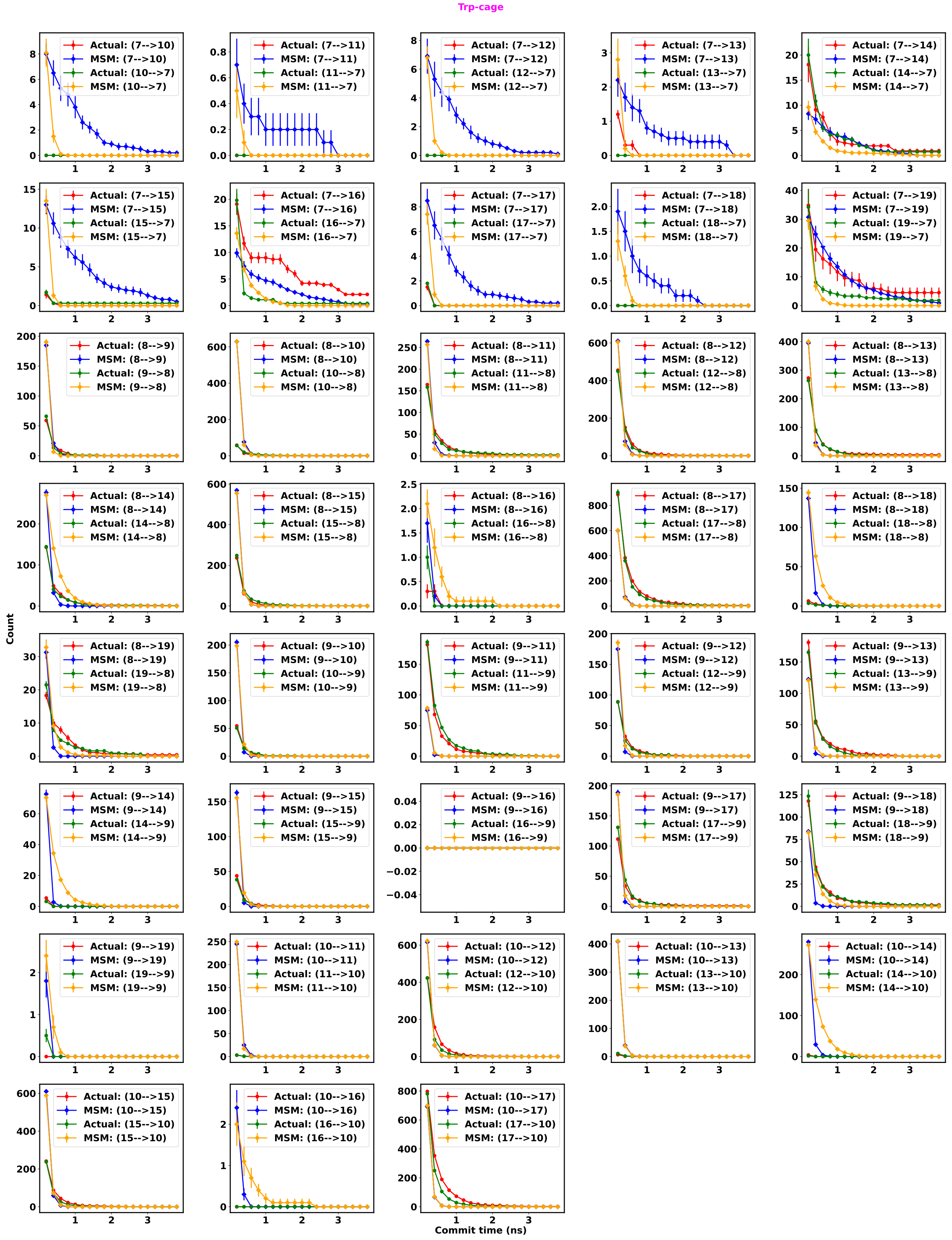

FIG. S36. State to state transition count as a function of commit time for Trp-cage mini protein with MSM using a large number of cluster.

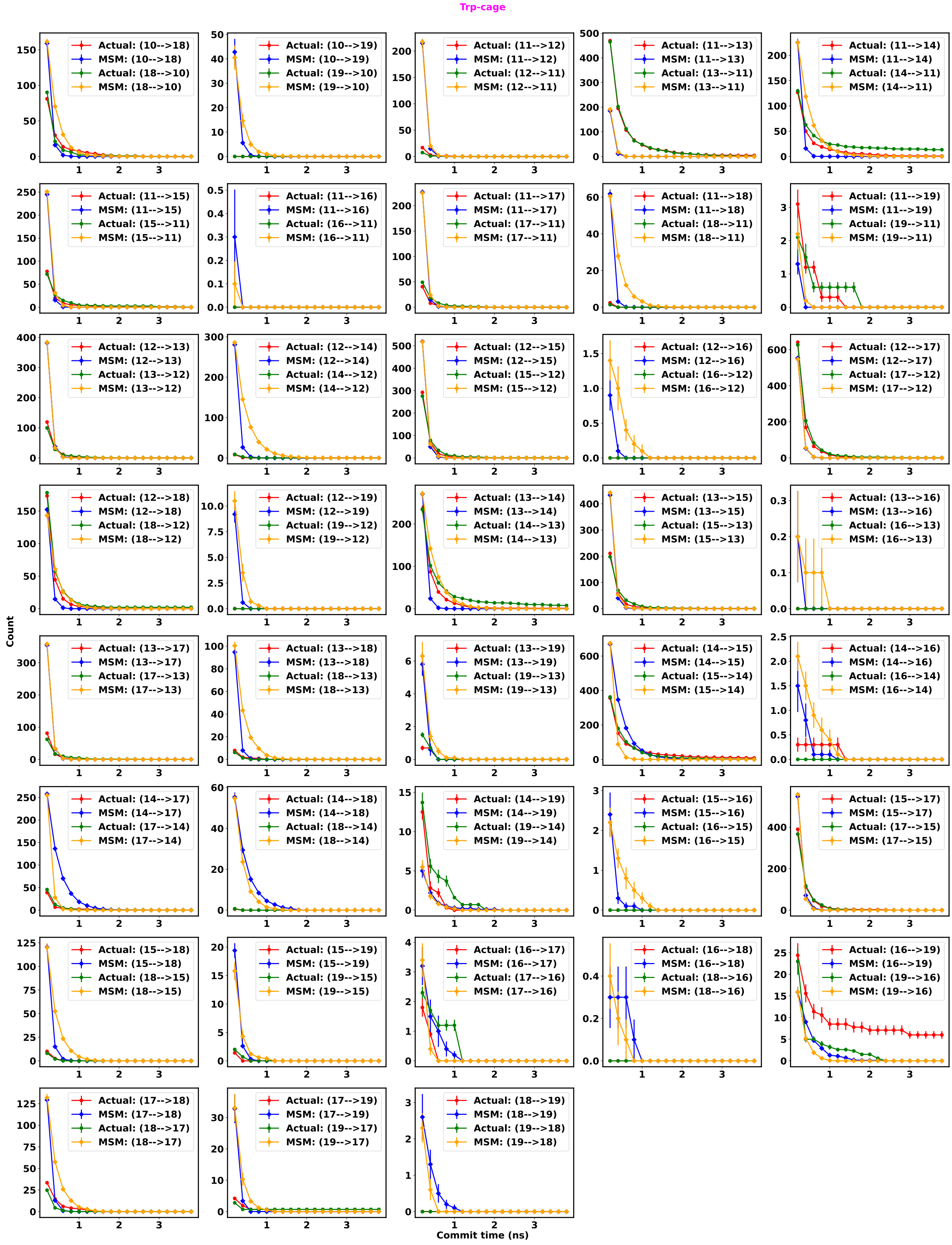

FIG. S37. State to state transition count as a function of commit time for Trp-cage mini protein with MSM using a large number of cluster.

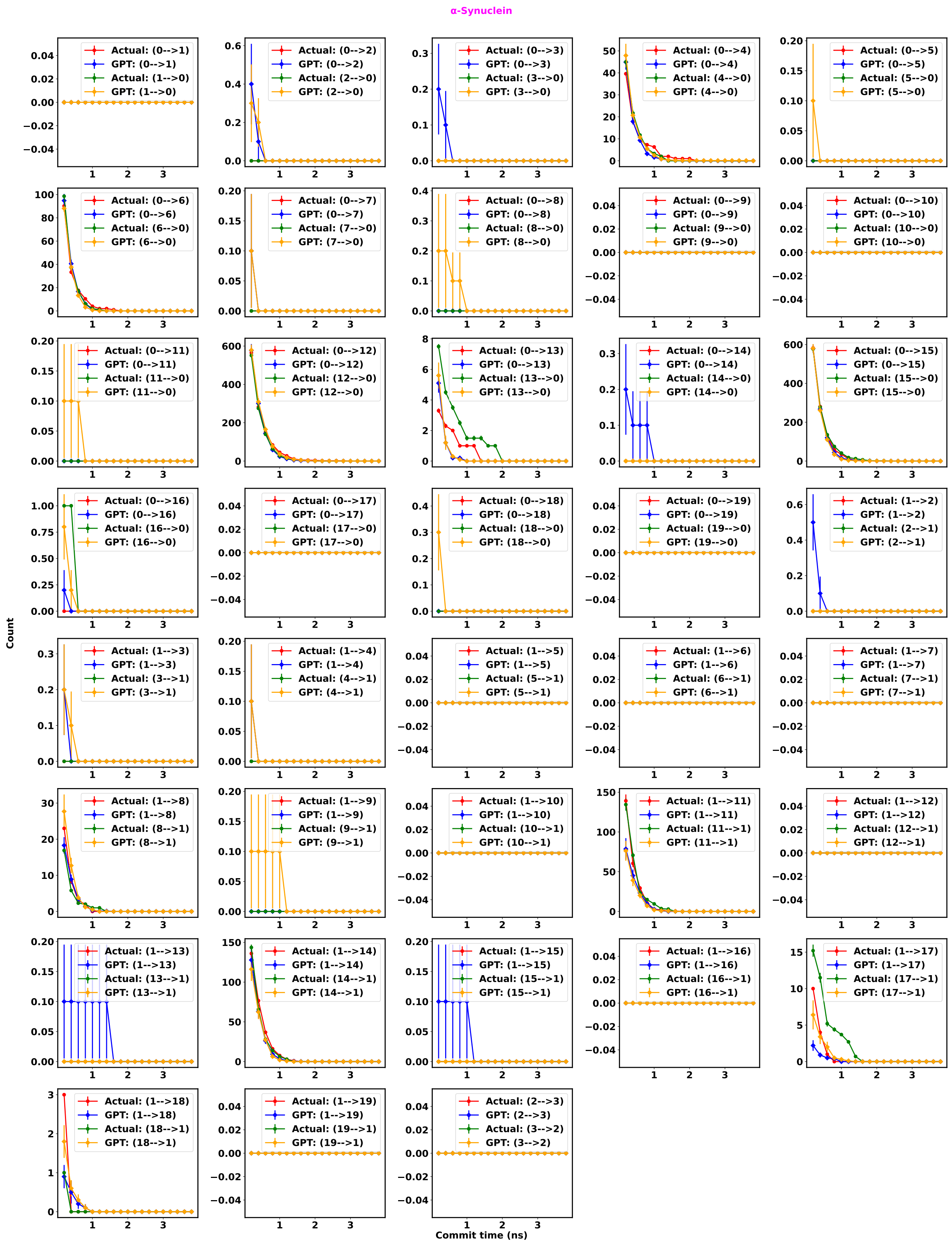

FIG. S38. State to state transition count as a function of commit time for α-Synuclein with GPT model using a large number of cluster.

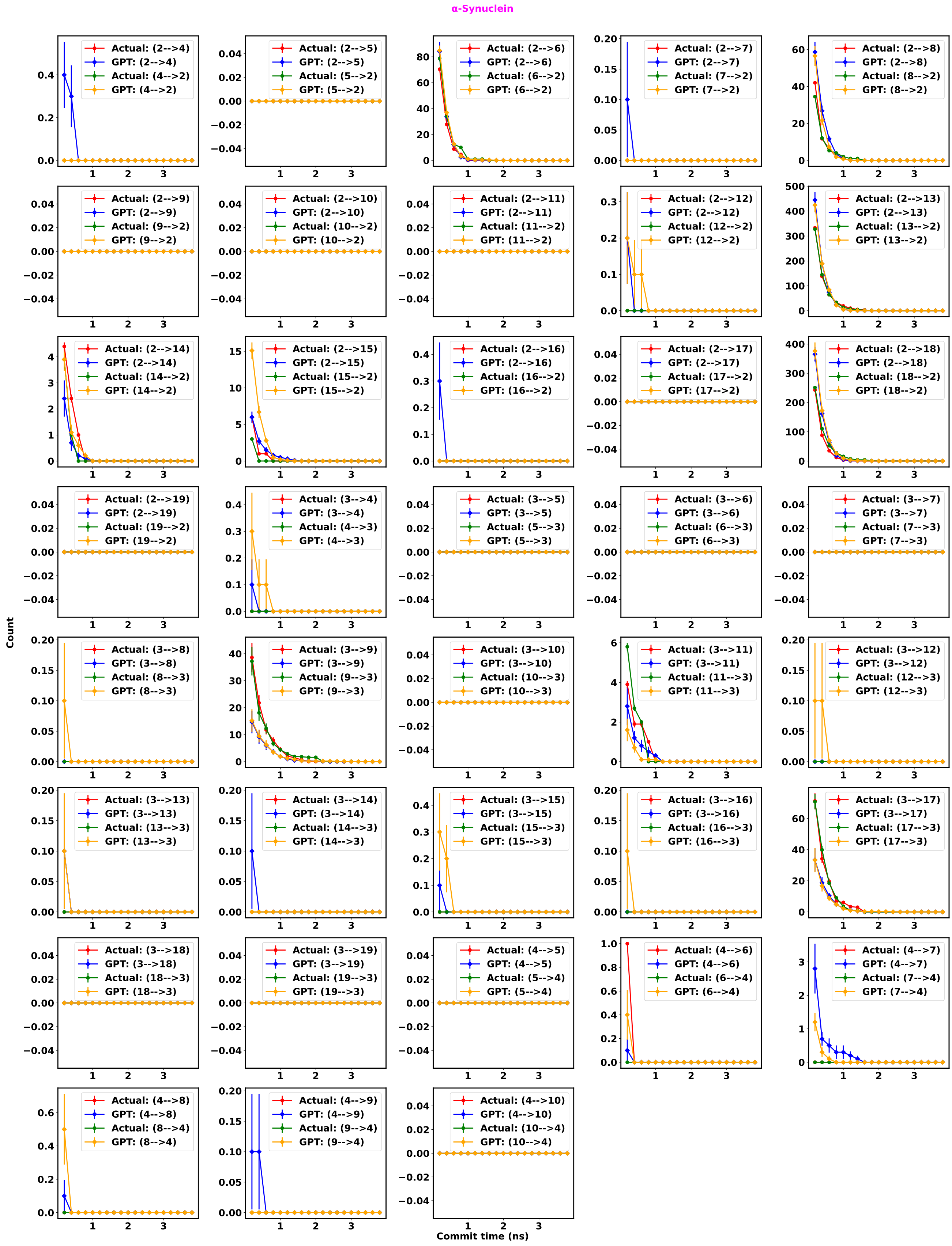

FIG. S39. State to state transition count as a function of commit time for α-Synuclein with GPT model using a large number of cluster.

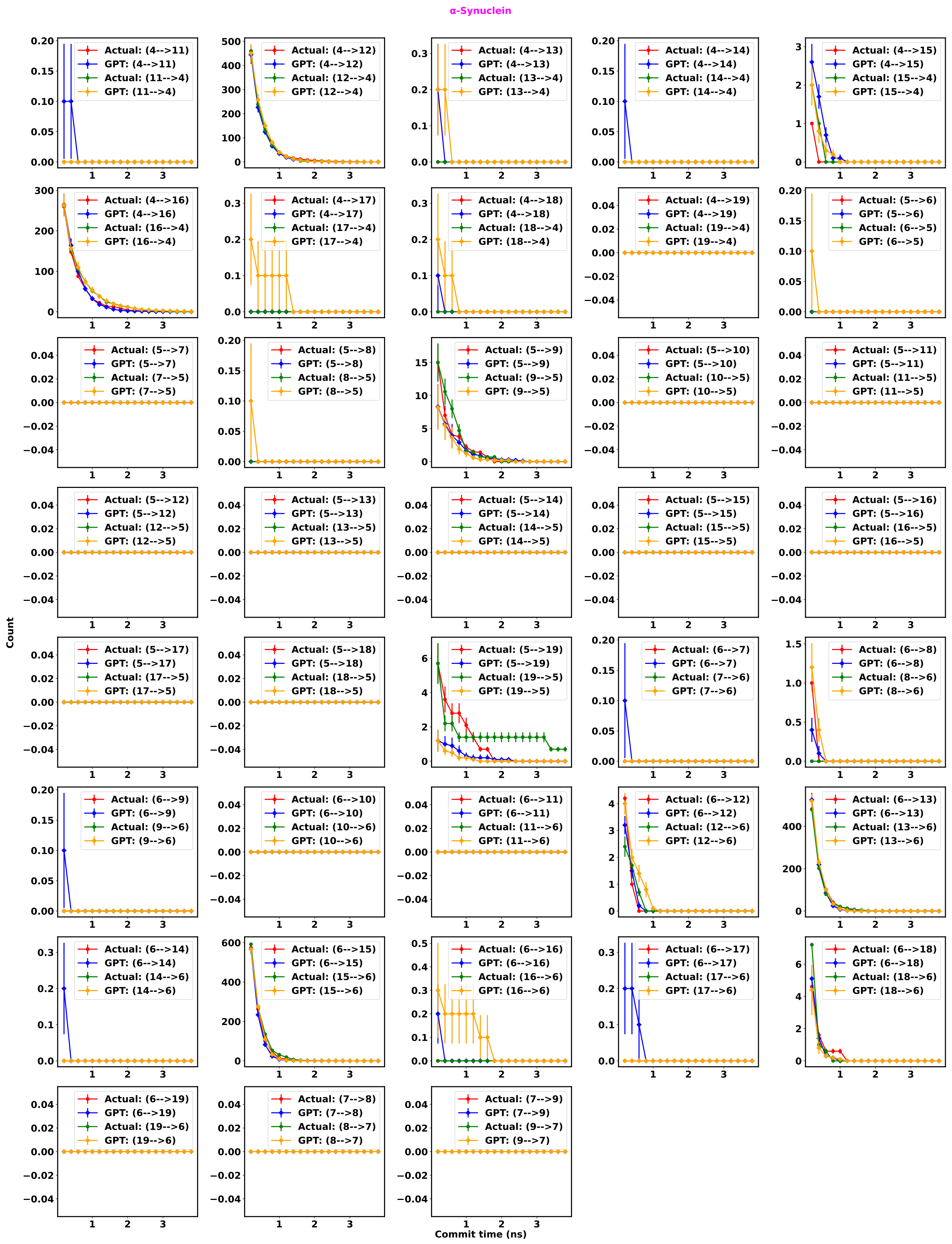

FIG. S40. State to state transition count as a function of commit time for α-Synuclein with GPT model using a large number of cluster.

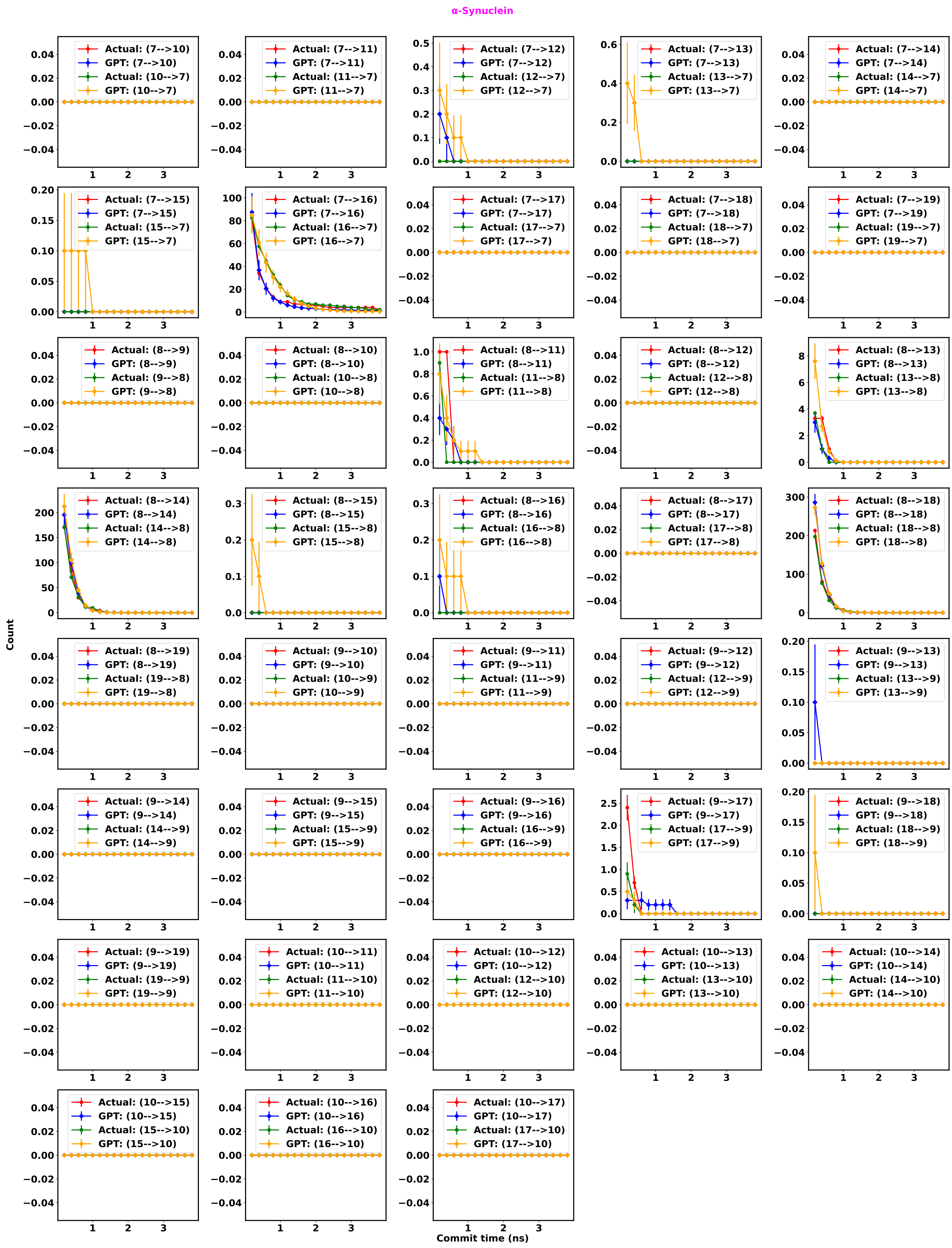

FIG. S41. State to state transition count as a function of commit time for α-Synuclein with GPT model using a large number of cluster.

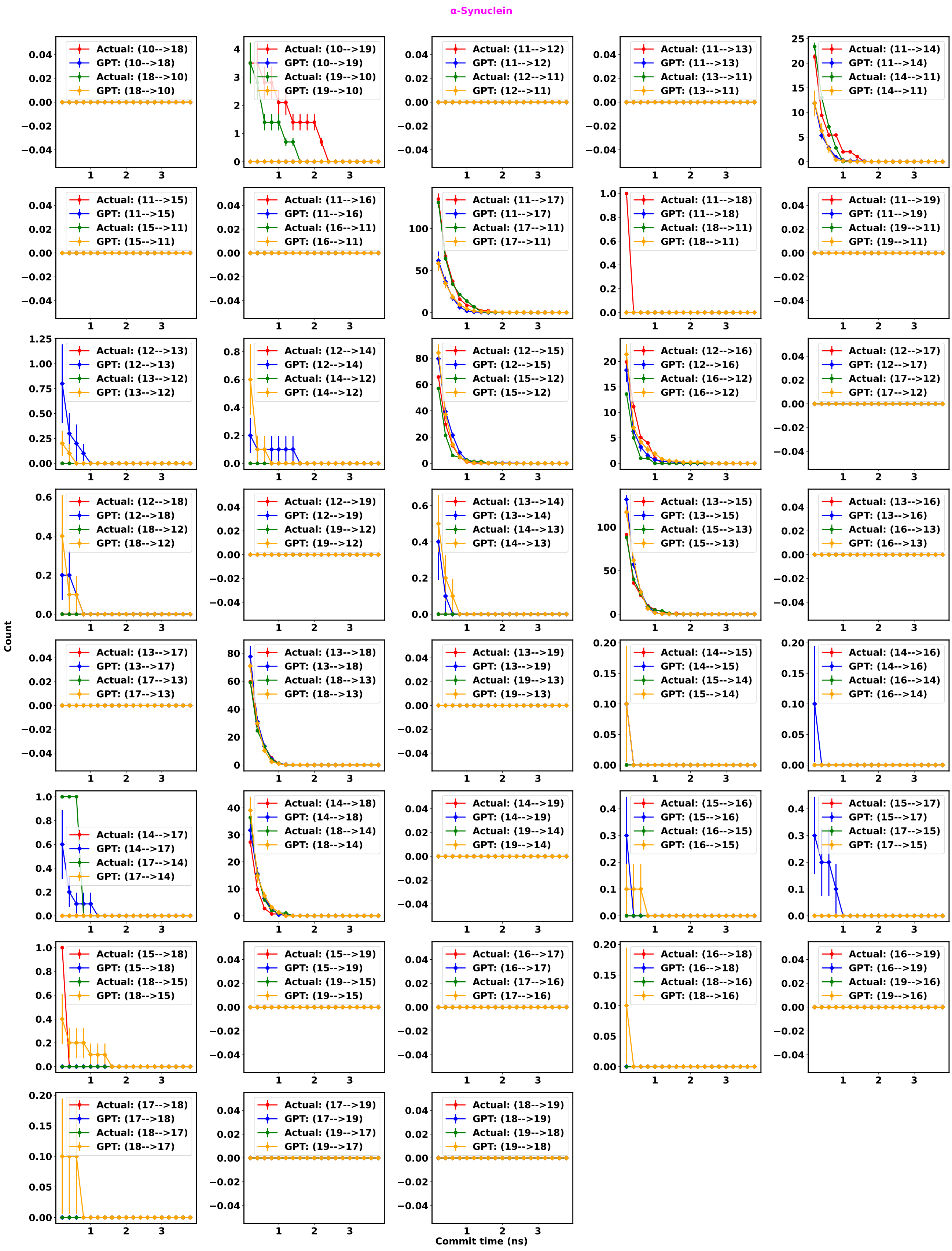

FIG. S42. State to state transition count as a function of commit time for α-Synuclein with GPT model using a large number of cluster.

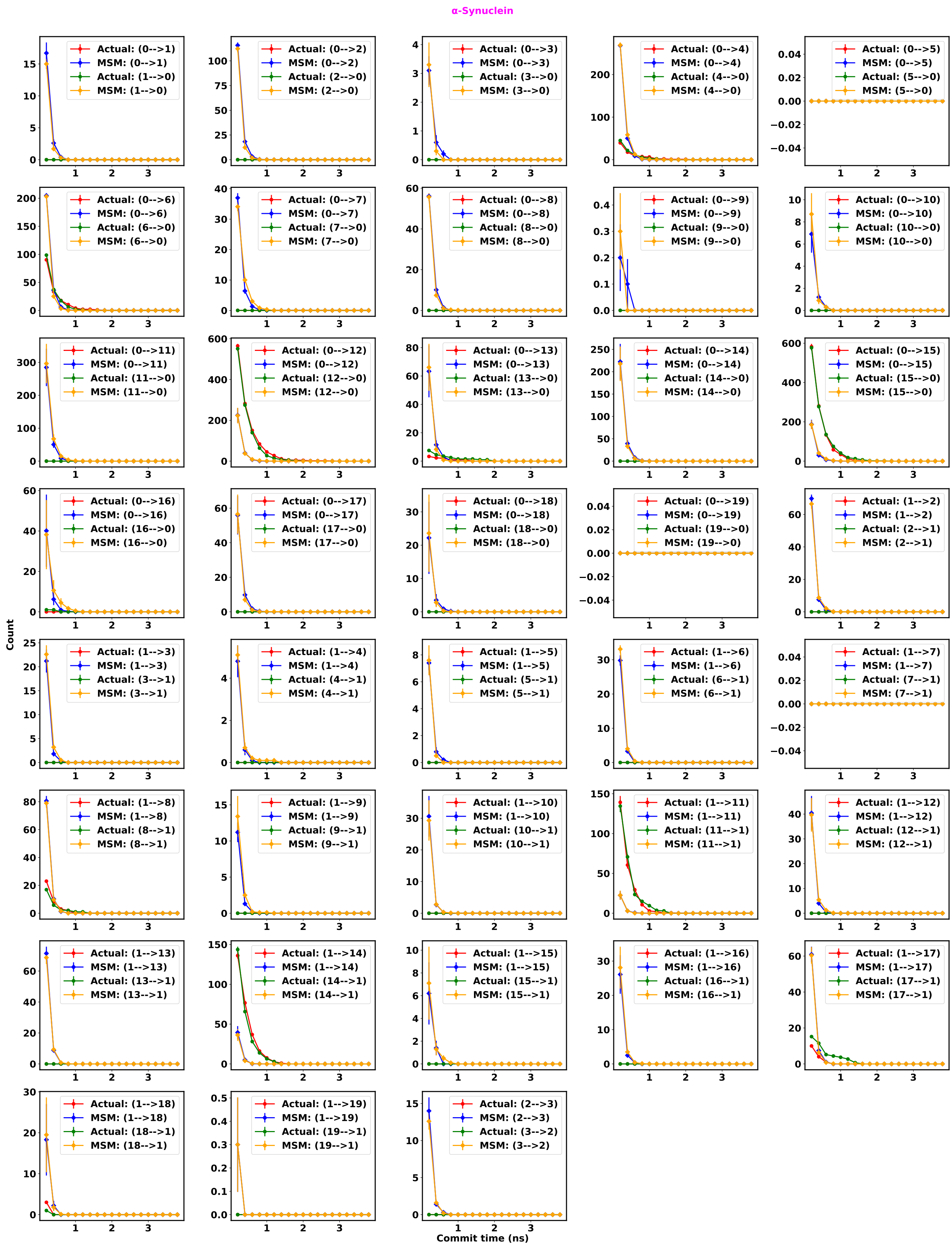

FIG. S43. State to state transition count as a function of commit time for α-Synuclein with MSM using a large number of cluster.

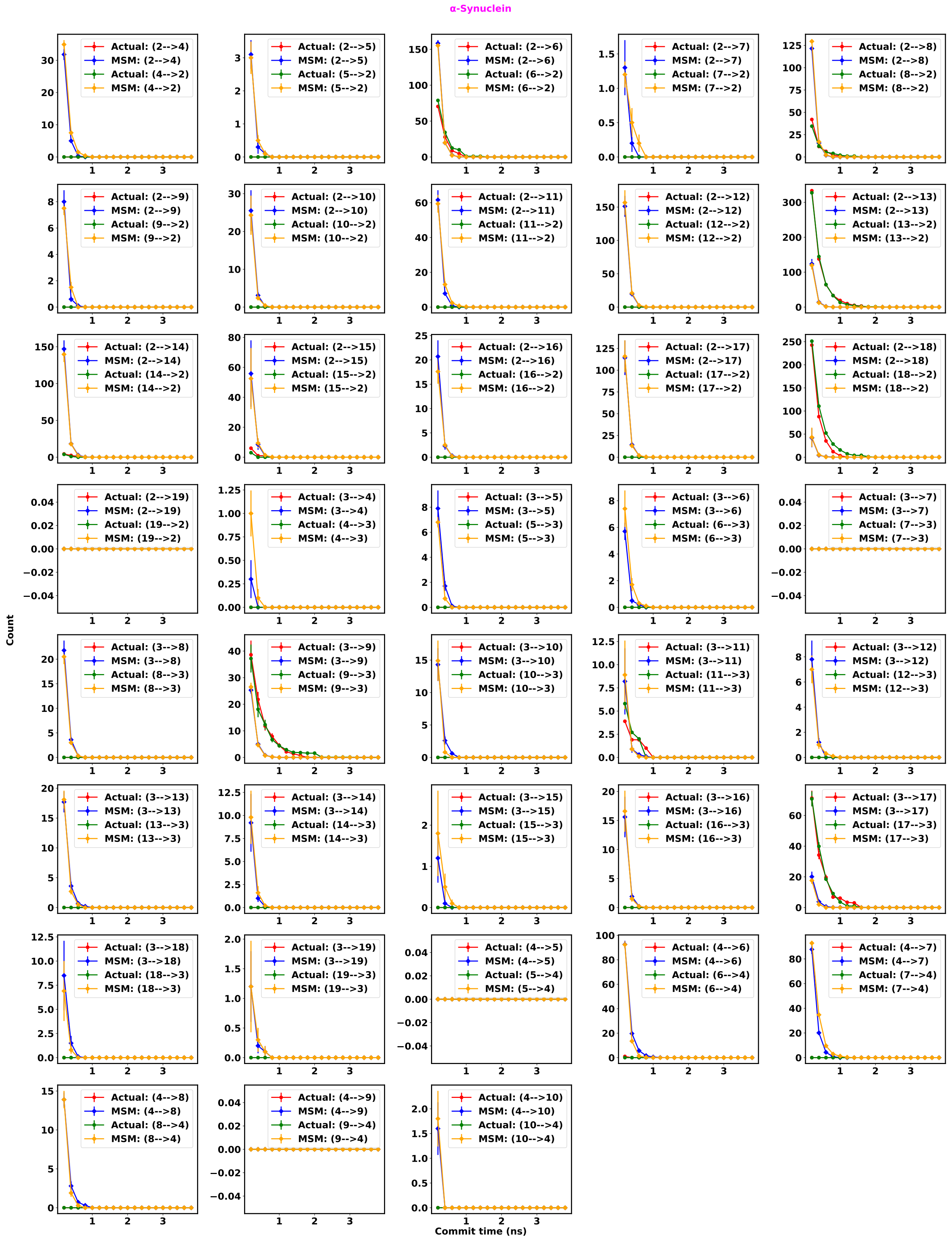

FIG. S44. State to state transition count as a function of commit time for α-Synuclein with MSM using a large number of cluster.

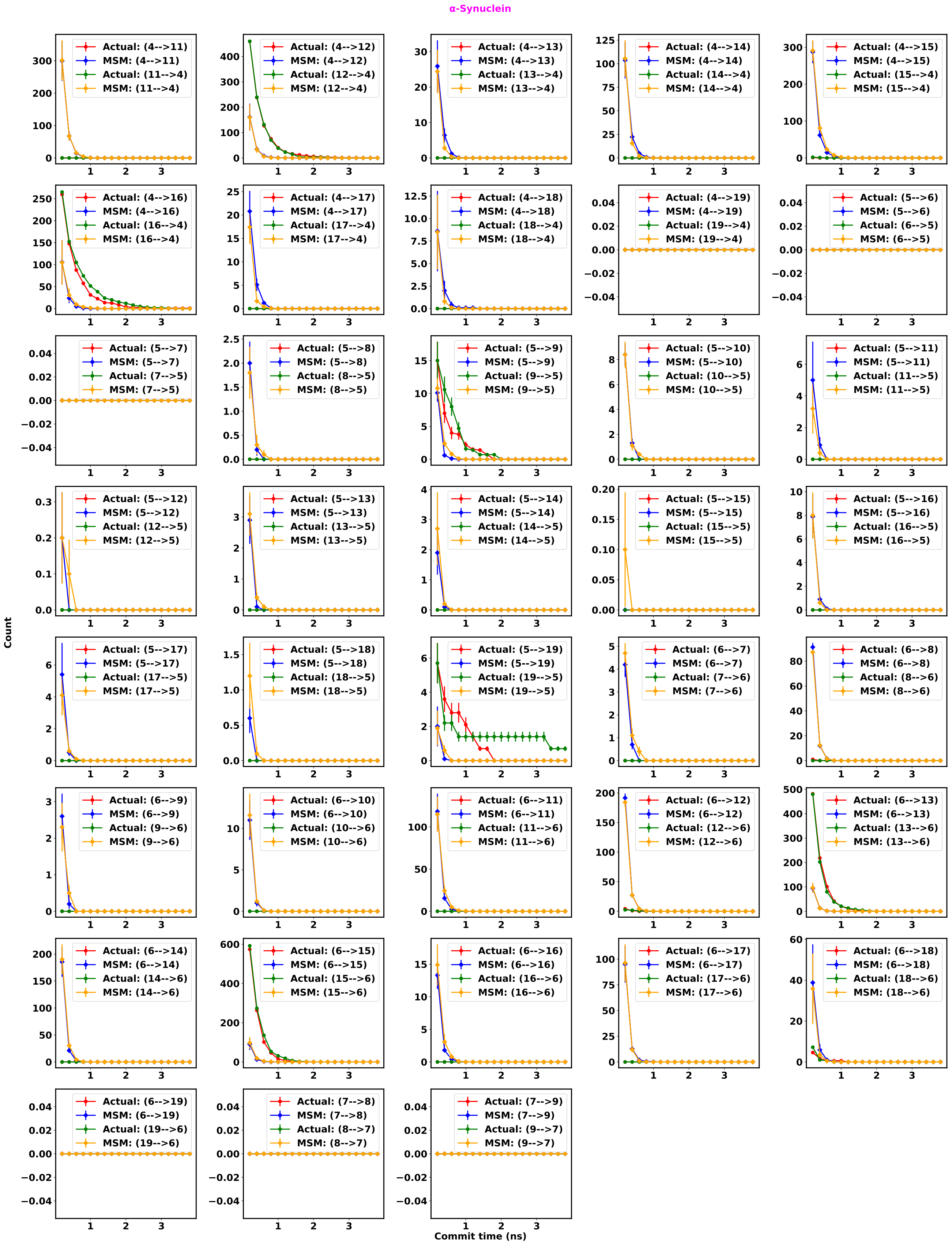

FIG. S45. State to state transition count as a function of commit time for α-Synuclein with MSM using a large number of cluster.

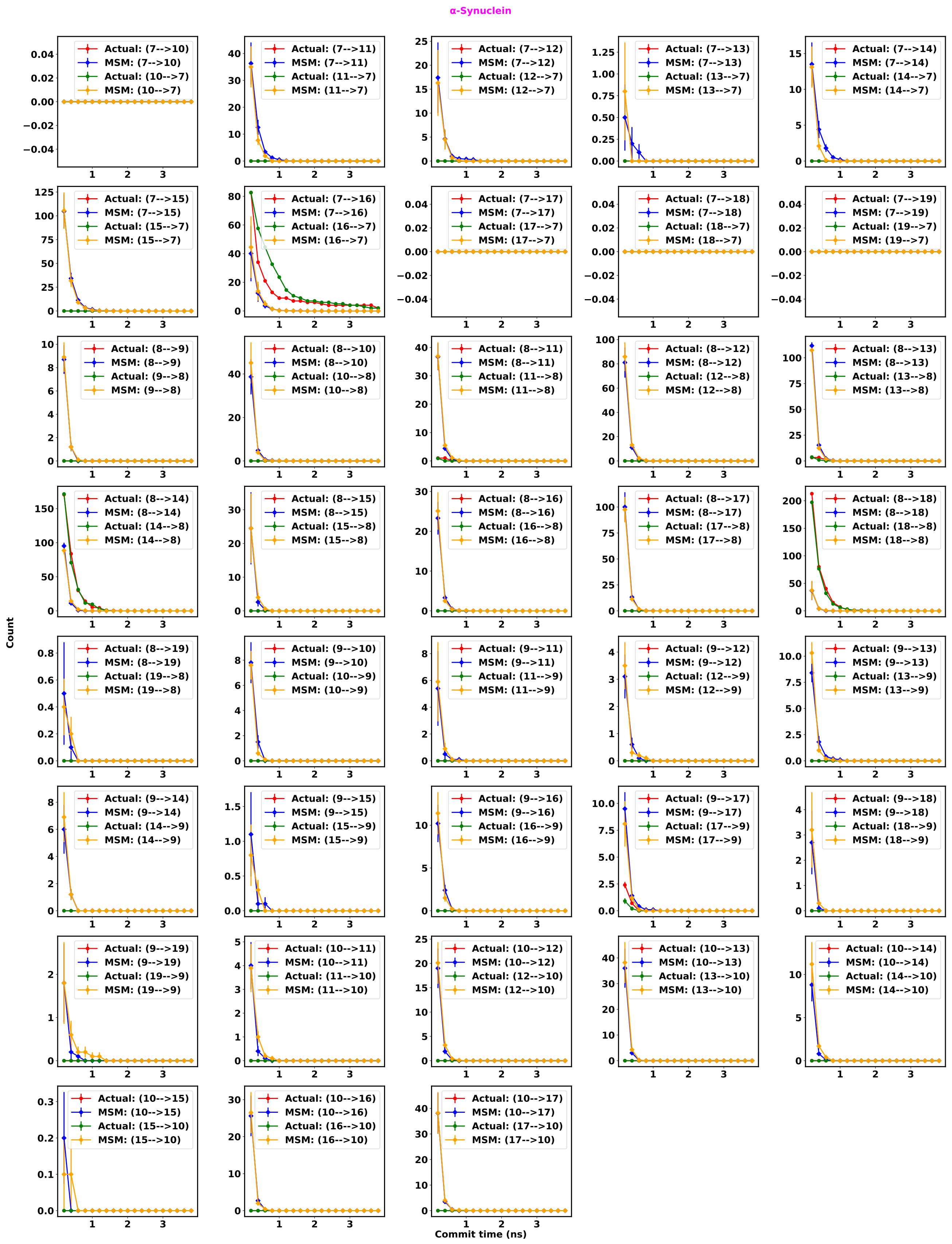

FIG. S46. State to state transition count as a function of commit time for α-Synuclein with MSM using a large number of cluster.

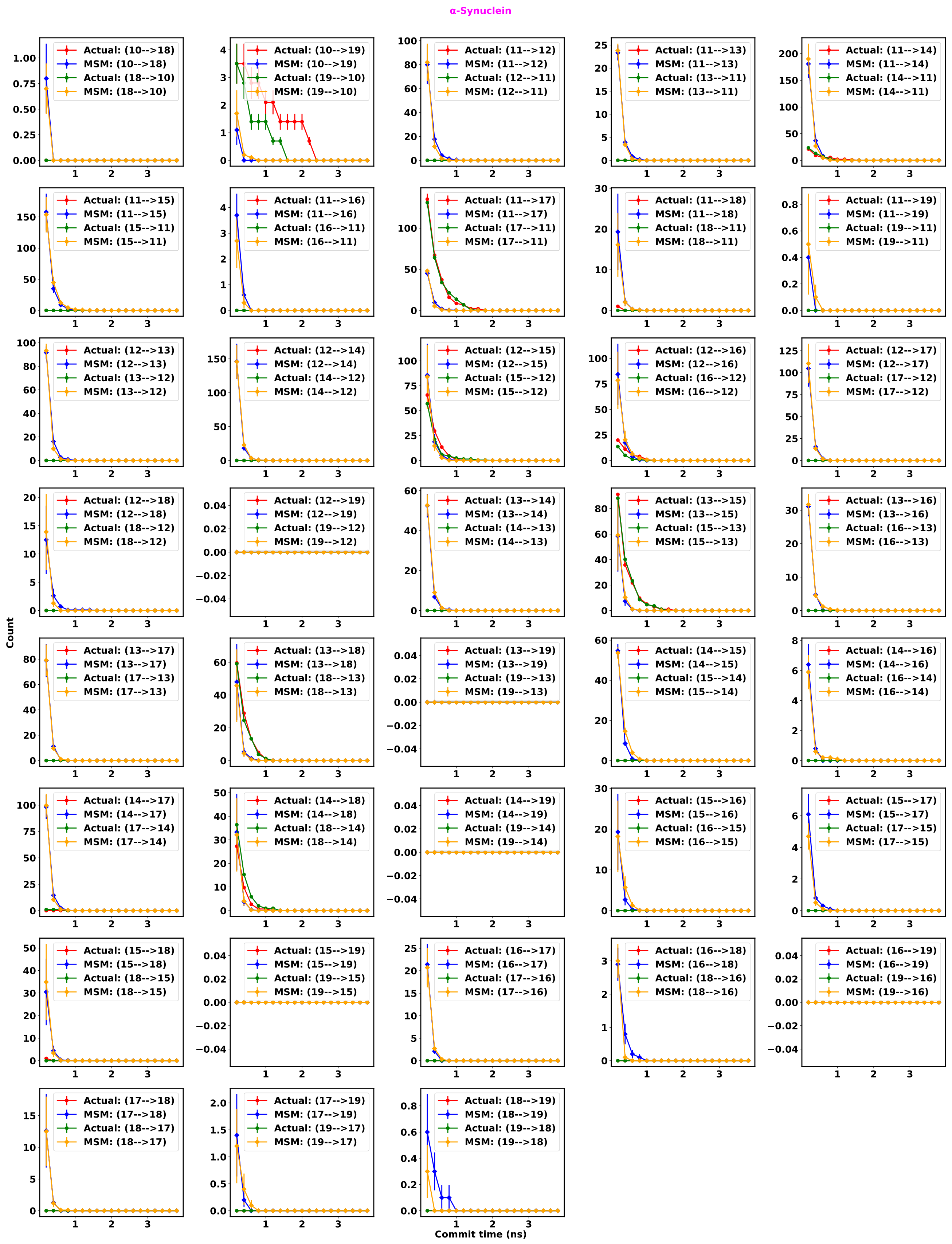

FIG. S47. State to state transition count as a function of commit time for α-Synuclein with MSM using a large number of cluster.

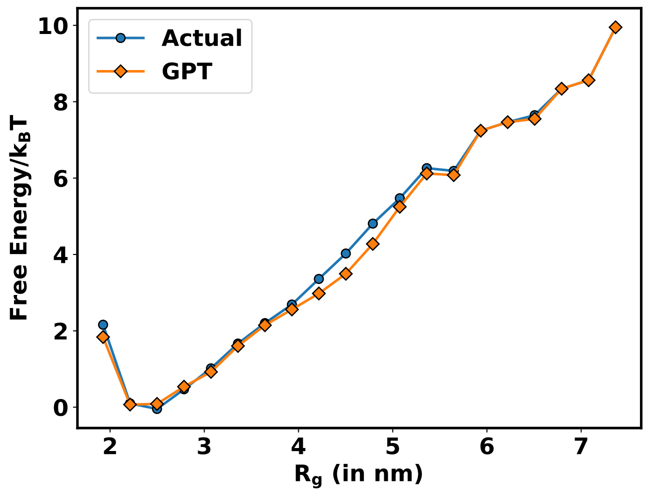

FIG. S48. Comparison between actual and GPT-generated one-dimensional free energy plot along radius of gyration (R_g)

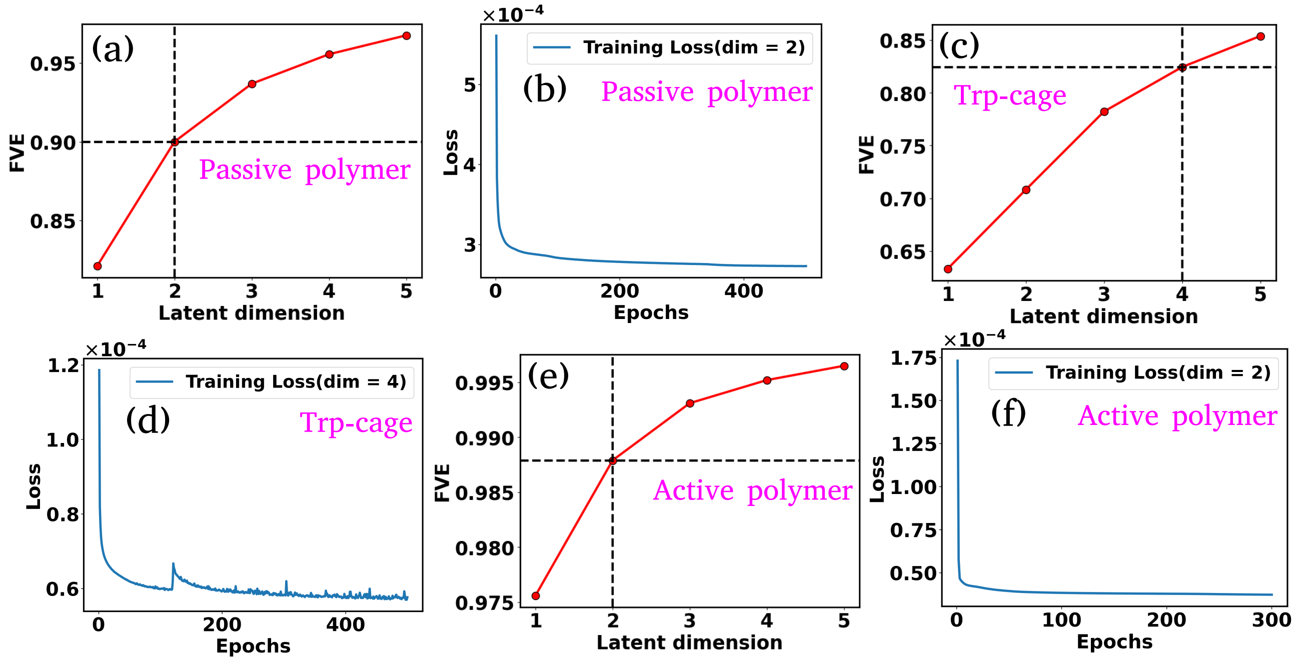

FIG. S49. (a-f) FVE and training loss plots for all systems. We have chosen 2D latent dimensions for active and passive polymer chains and 4D latent dimensions for Trp-cage mini protein.

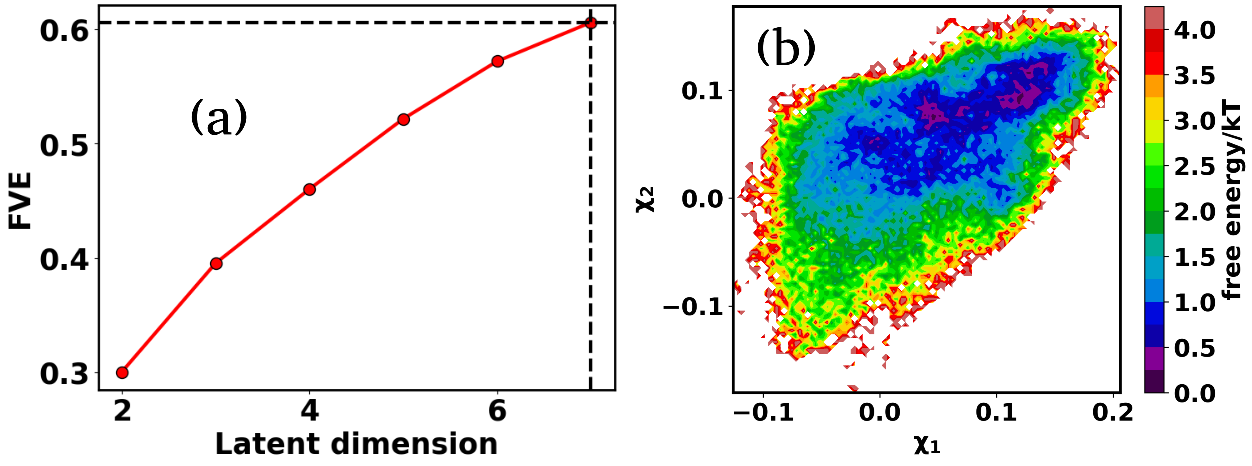

FIG. S50. (a) The FVE score as a function of latent dimension for α-Synuclein. The value of the FVE score is approximately 0.60, even with a high latent dimension of $L_d = 7$. (b) The FES plot along the first two latent dimensions for α-Synuclein reveals no distinct minima. As a result, state decomposition through clustering within this latent space is unfeasible.

TABLE S1. The amount of data used for training, validation and testing

System	Total data	Training	Validation	Prompt	Testing or generation
3-state	50,00000	18,00000	2,00000	128	2,00000
4-state	50,00000	18,00000	2,00000	128	2,00000
Passive polymer	57,47973	18,00000	2,00000	128	2,00000
Active polymer	50,00000	18,00000	2,00000	128	2,00000
Trp-cage	5,00000	2,70000	30,000	128	1,00000
α-Synuclein	73,124	50,000	5,000	128	20,000

TABLE S2. The hyperparameters used for training the GPT model.

System	batch size	block size	epochs	embeddings(d)	head(N_h)	blocks(N_b)	learning rate	optimizer
3-state	128	128	10000	256	8	8	10^{-4}	AdamW [1]
4-state	128	128	10000	256	8	8	10^{-4}	AdamW
Passive polymer	128	128	10000	256	8	8	10^{-4}	AdamW
Active polymer	128	128	10000	256	8	8	10^{-4}	AdamW
Trp-cage	128	128	10000	256	8	8	10^{-4}	AdamW
α-Synuclein	128	384	10000	256	8	8	10^{-4}	AdamW

TABLE S3. The hyperparameters used for training the Autoencoder. Here L_d represents the latent dimensions

System	batch size	epochs	Activation	learning rate	optimizer	loss function	architecture
Passive polymer	100	500	Elu	5×10^{-4}	Adam [2]	MSE	28-12-L_d-12-28
Active polymer	100	300	Elu	5×10^{-4}	Adam	MSE	28-12-L_d-12-28
Trp-cage	64	500	Elu	5×10^{-4}	Adam	MSE	190-72-36-12-L_d -12-36-72-190
α-Synuclein	64	100	Tanh	10^{-4}	Adam	MSE	9730-4096-1024-512-128-16-L_d -16-128-512-1024-4096-9730

TABLE S4. CPU and GPU memory usage for both the MD simulation and the GPT model.

System	Process	CPU (GB)	GPU (MB)
Trp-cage	MD	$\sim$ 0.20	$\sim$ 296.4
Trp-cage	GPT	$\sim$ 0.70	$\sim$ 428.0
α-Synuclein	MD	$\sim$ 1.10	$\sim$482.4
α-Synuclein	GPT	$\sim$ 0.70	$\sim$702.4

TABLE S5. The hyperparameters used for training the LSTM model.

System	batch size	block size	epochs	embeddings(d)	LSTM unit	learning rate	optimizer
3-state	128	128	100	256	32	10^{-3}	Adam [2]
Active polymer	128	128	500	256	32	10^{-3}	Adam
Trp-cage	128	128	100	256	32	10^{-3}	Adam
α-Synuclein	128	384	100	256	32	10^{-3}	Adam

[1] Ilya Loshchilov and Frank Hutter. Decoupled weight decay regularization. *arXiv preprint arXiv:1711.05101*, 2017.

[2] Diederik P Kingma and Jimmy Ba. Adam: A method for stochastic optimization. *arXiv preprint arXiv:1412.6980*, 2014.
